# Supplementary figures and images for: LATS1/2 inactivation in the mammary epithelium drives the evolution of a tumor-associated niche
Source: EMBO Rep. 2025 Feb 14;26(6):1472–503. doi: 10.1038/s44319-025-00370-3 (PMC11933708; doi:10.1038/s44319-025-00370-3)

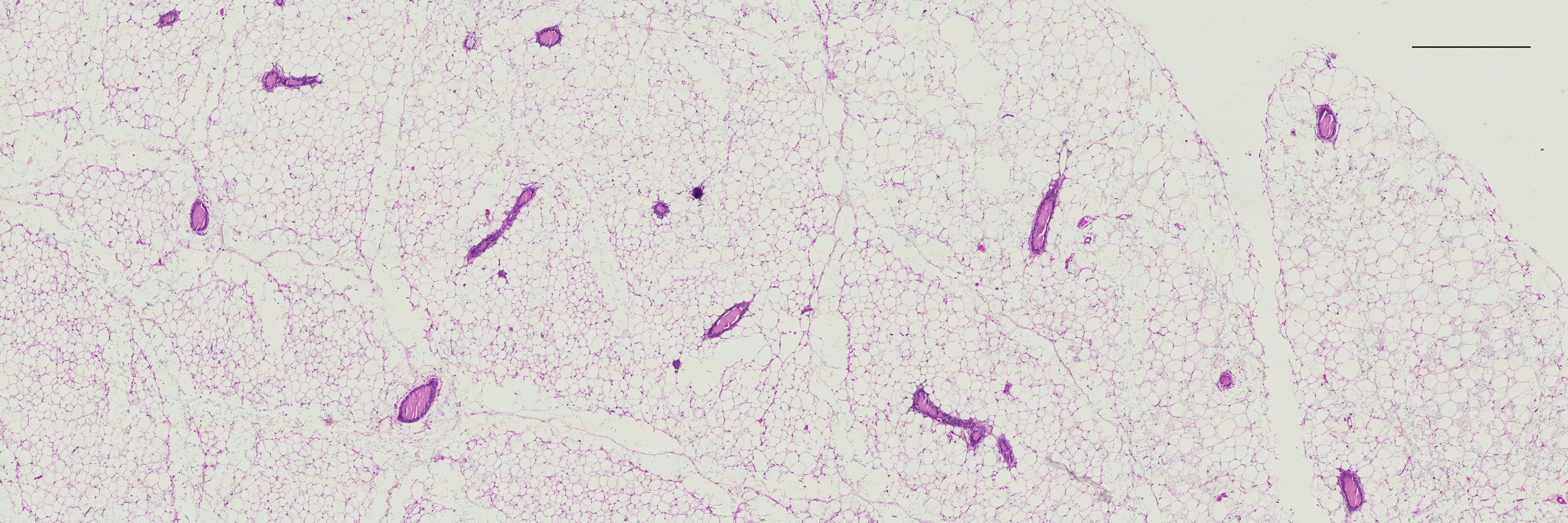

Supplement: Supplementary file 4 — Source data Fig. 1 [file 44319_2025_370_MOESM4_ESM.zip › Source Data Fig 1/1A/H&E Control Large.tif]

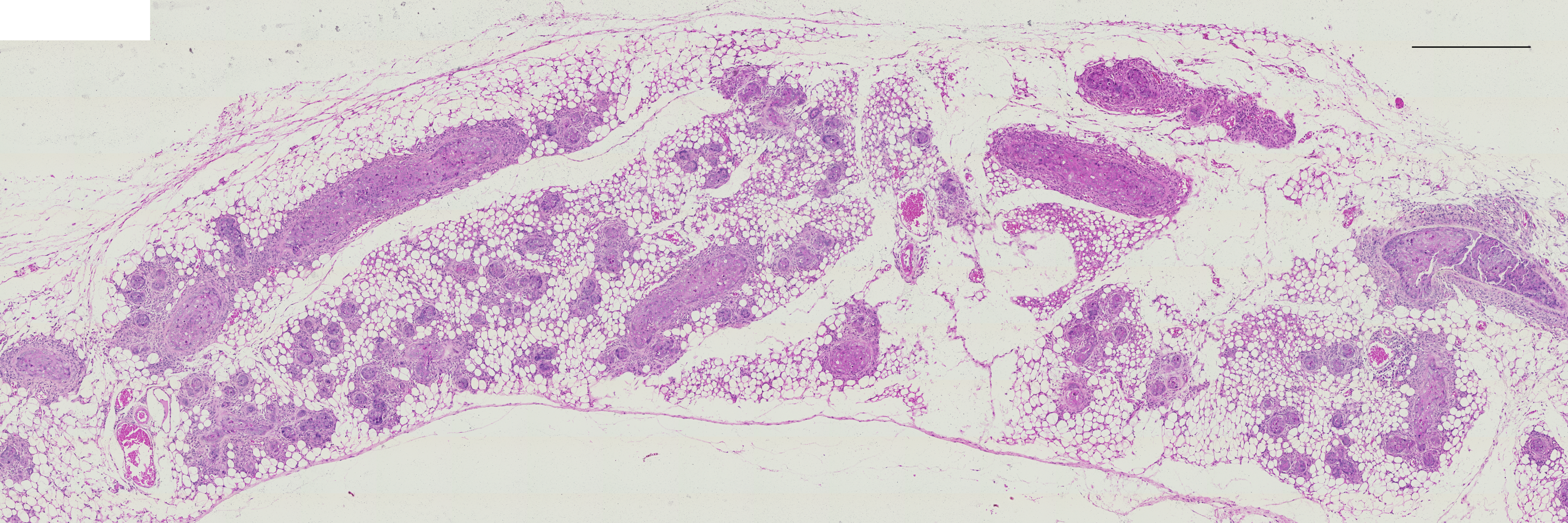

Supplement: Supplementary file 4 — Source data Fig. 1 [file 44319_2025_370_MOESM4_ESM.zip › Source Data Fig 1/1A/H&E L12KO Large.tif]

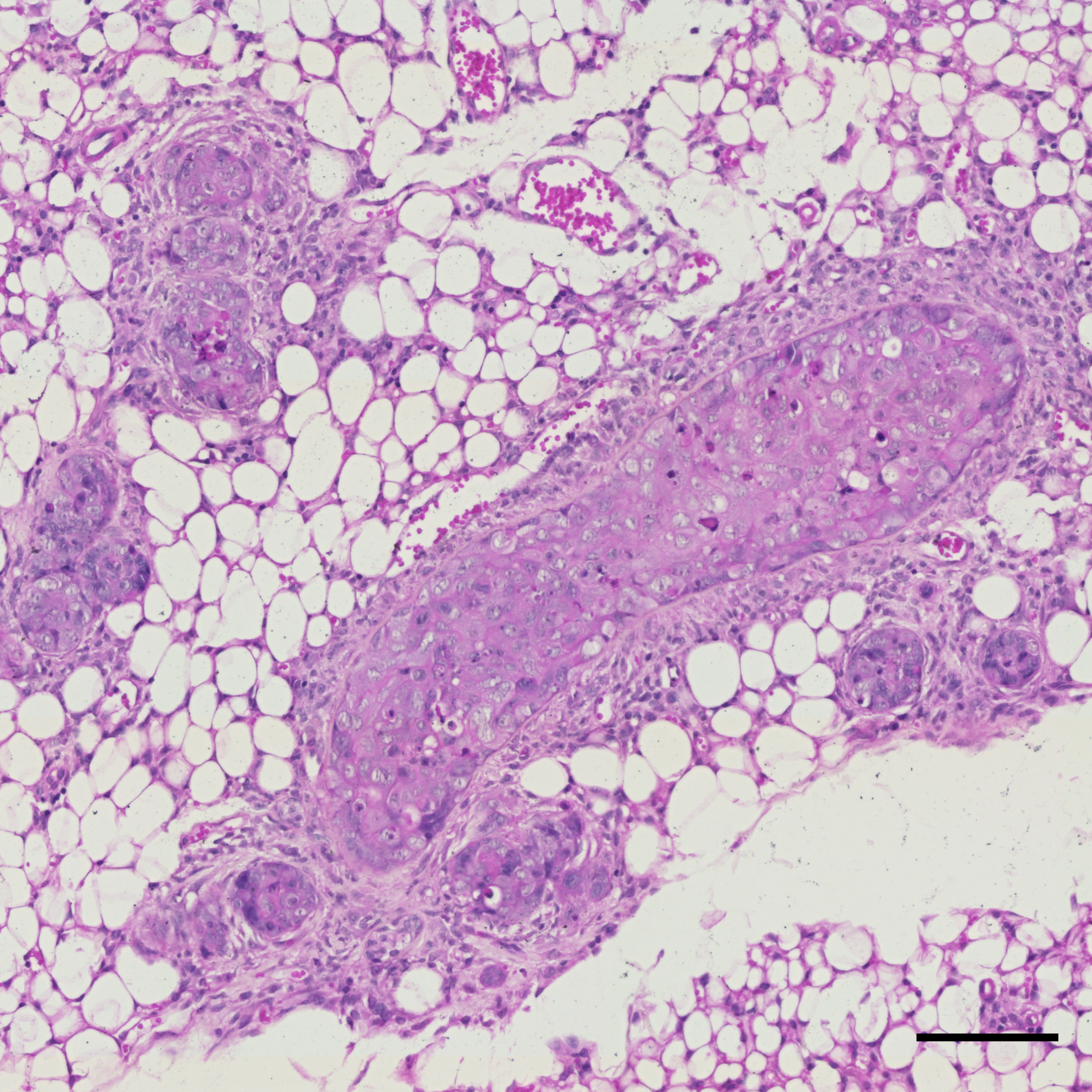

Supplement: Supplementary file 4 — Source data Fig. 1 [file 44319_2025_370_MOESM4_ESM.zip › Source Data Fig 1/1A/H&E L12KO Small.tif]

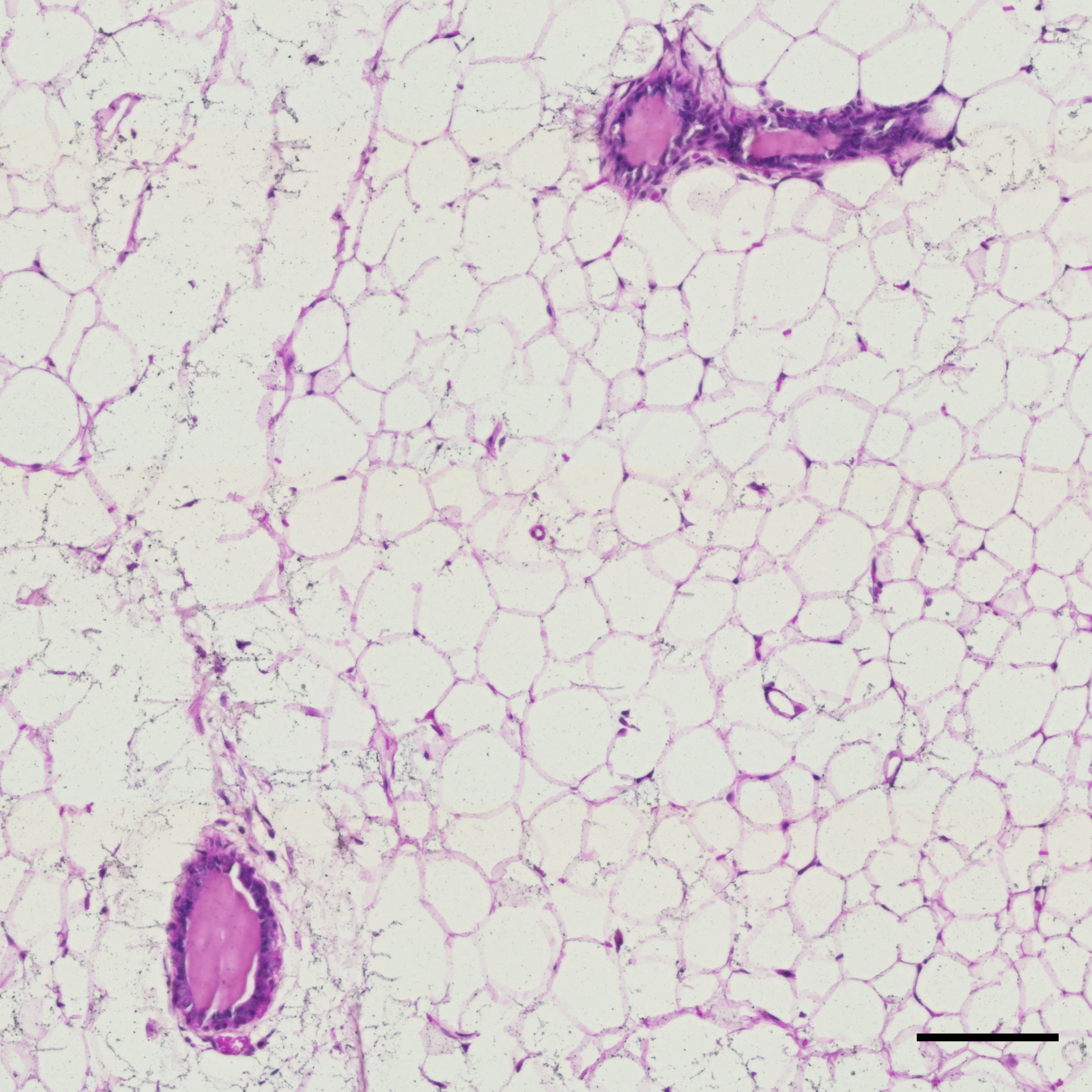

Supplement: Supplementary file 4 — Source data Fig. 1 [file 44319_2025_370_MOESM4_ESM.zip › Source Data Fig 1/1A/H&E Control Small.tif]

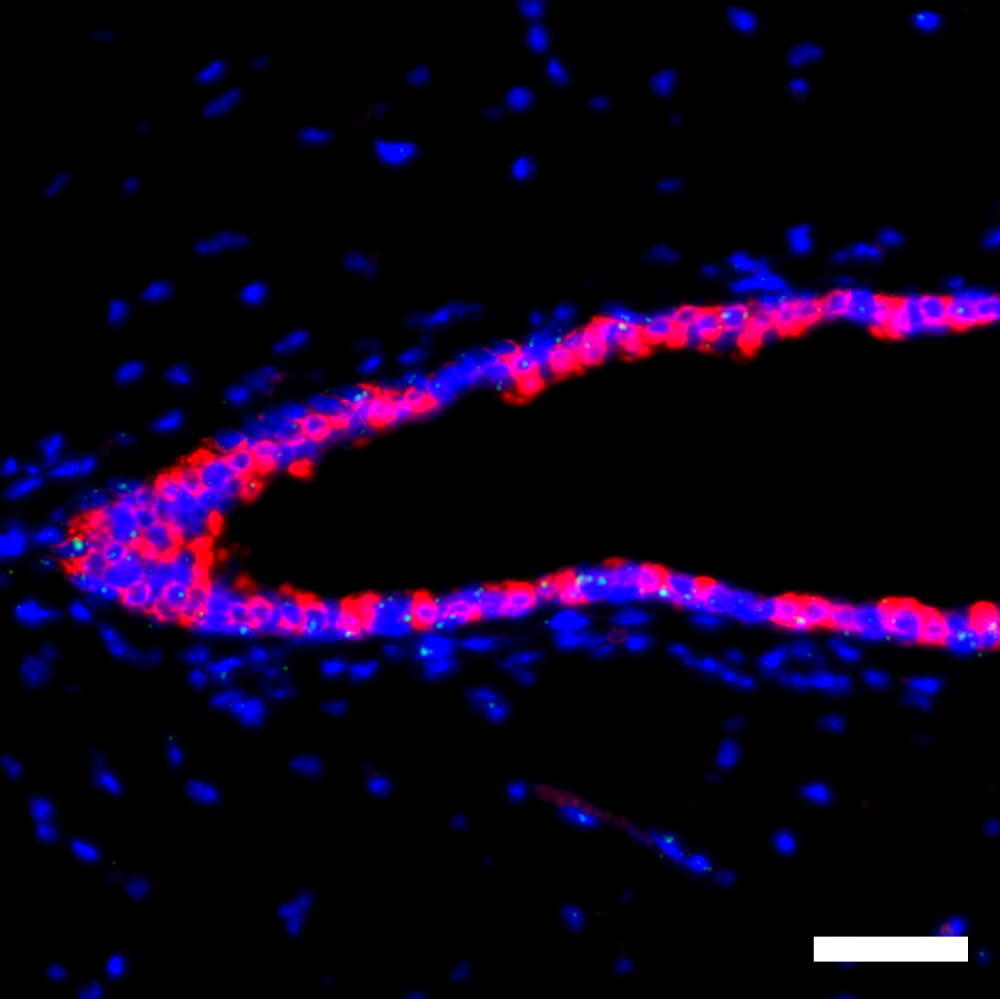

Supplement: Supplementary file 5 — Source data Fig. 3 [file 44319_2025_370_MOESM5_ESM.zip › Source Data Fig 3/3K/CTL K8 Pdgfarna.tif]

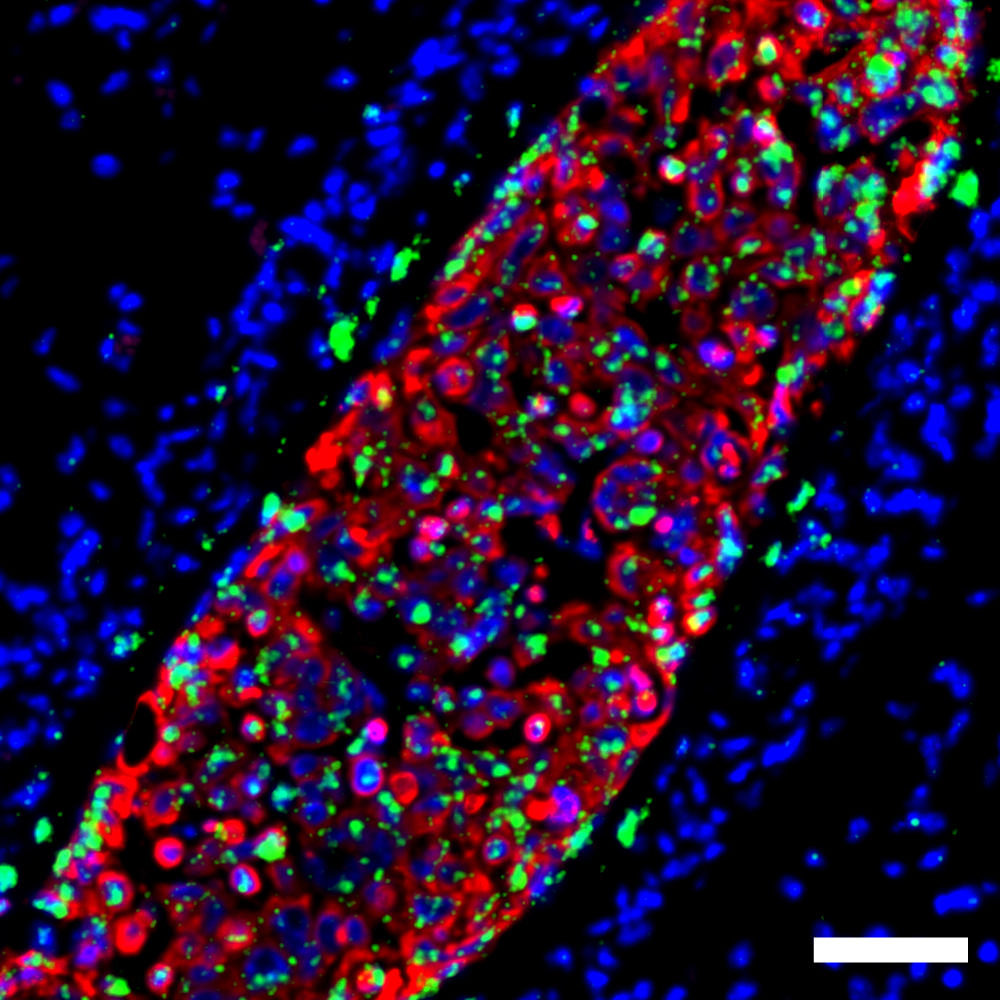

Supplement: Supplementary file 5 — Source data Fig. 3 [file 44319_2025_370_MOESM5_ESM.zip › Source Data Fig 3/3K/L12KO K8 Pdgfarna.tif]

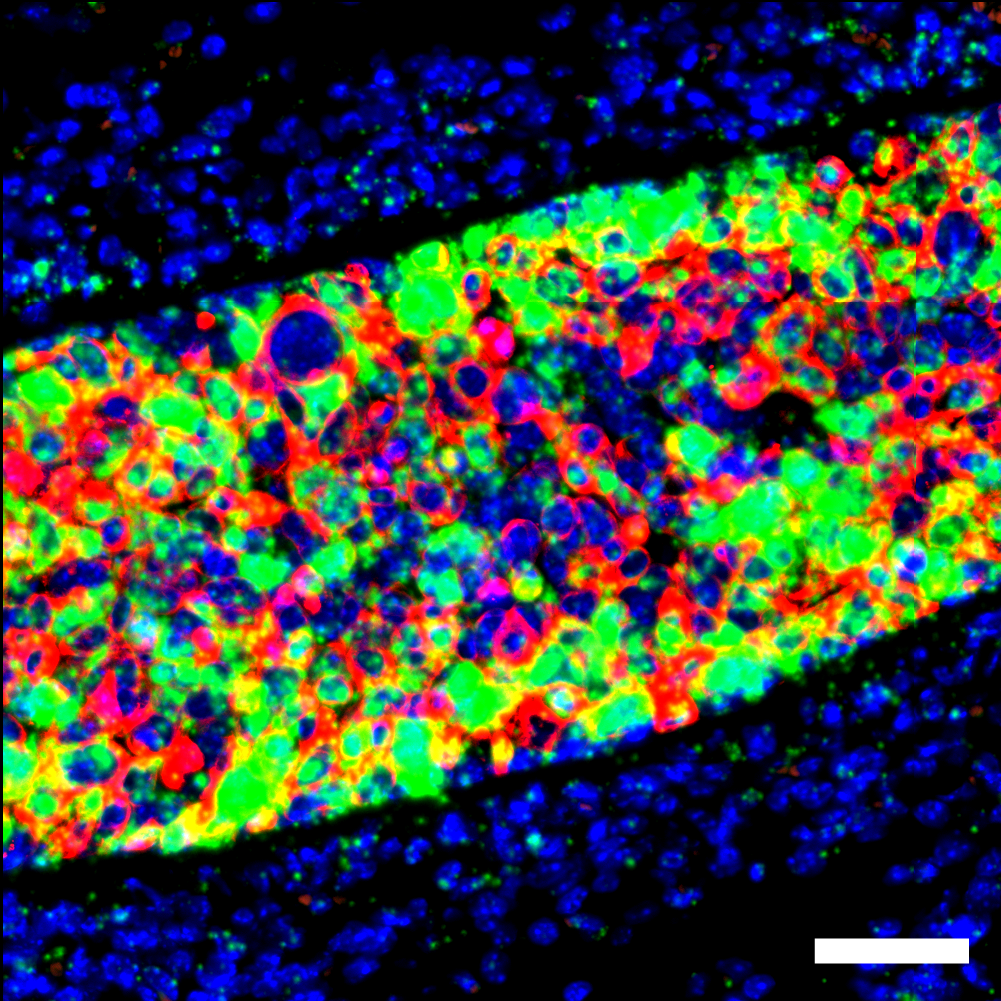

Supplement: Supplementary file 5 — Source data Fig. 3 [file 44319_2025_370_MOESM5_ESM.zip › Source Data Fig 3/3L/L12KO K8 Pdgfbrna.tif]

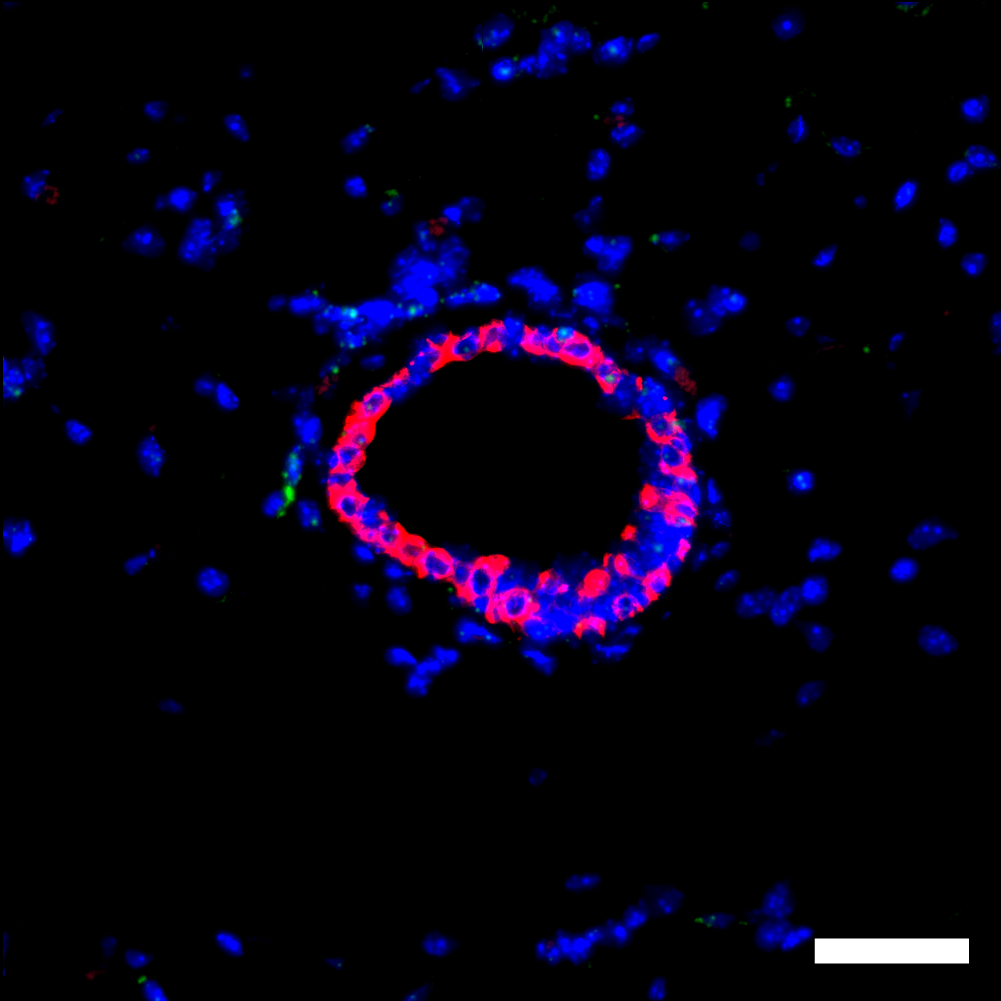

Supplement: Supplementary file 5 — Source data Fig. 3 [file 44319_2025_370_MOESM5_ESM.zip › Source Data Fig 3/3L/CTL K8 Pdgfbrna.tif]

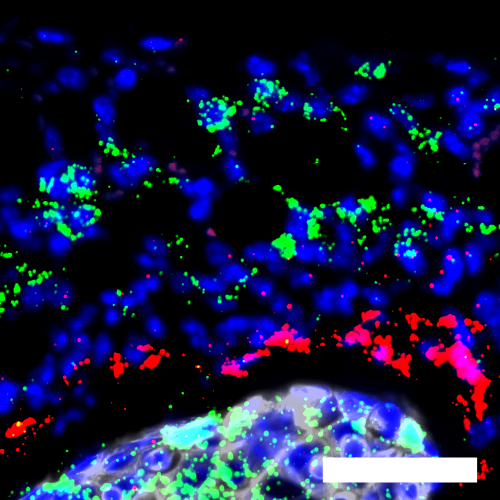

Supplement: Supplementary file 5 — Source data Fig. 3 [file 44319_2025_370_MOESM5_ESM.zip › Source Data Fig 3/3E/L12KO Subset K8 Lrrc15rna Scara5rna.tif]

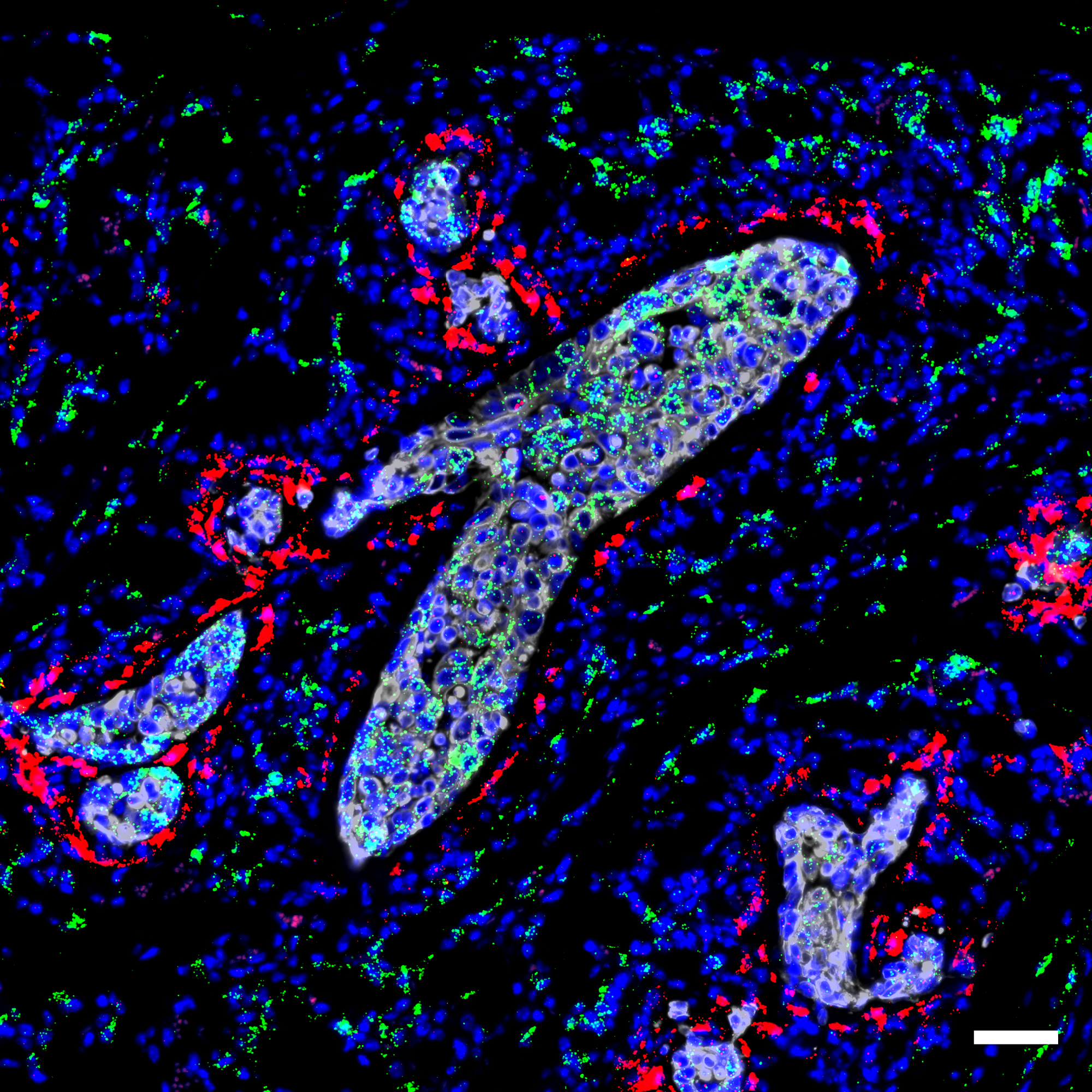

Supplement: Supplementary file 5 — Source data Fig. 3 [file 44319_2025_370_MOESM5_ESM.zip › Source Data Fig 3/3E/L12KO K8 Lrrc15rna Scara5rna.tif]

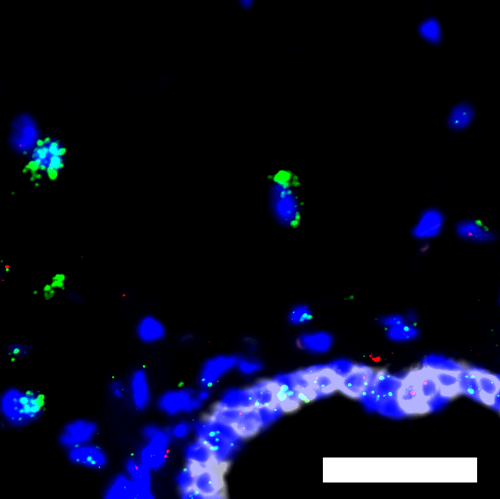

Supplement: Supplementary file 5 — Source data Fig. 3 [file 44319_2025_370_MOESM5_ESM.zip › Source Data Fig 3/3E/CTL Subset K8 Lrrc15rna Scara5rna.tif]

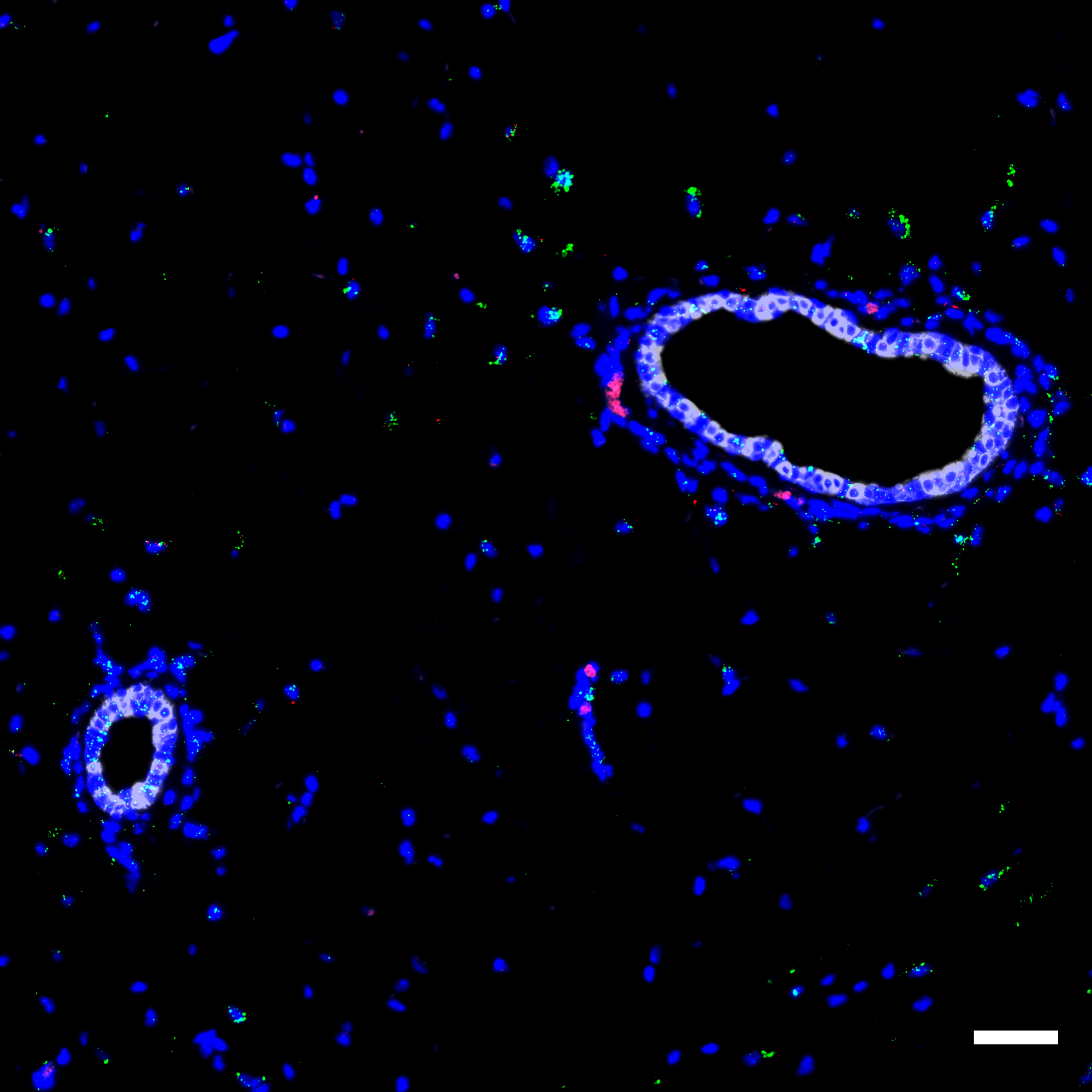

Supplement: Supplementary file 5 — Source data Fig. 3 [file 44319_2025_370_MOESM5_ESM.zip › Source Data Fig 3/3E/CTL K8 Lrrc15rna Scara5rna.tif]

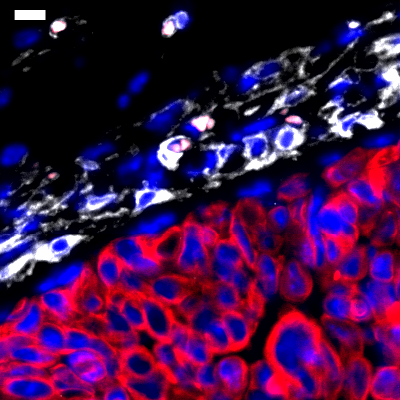

Supplement: Supplementary file 5 — Source data Fig. 3 [file 44319_2025_370_MOESM5_ESM.zip › Source Data Fig 3/3B/LKO Subset K8 PDGFRB.tif]

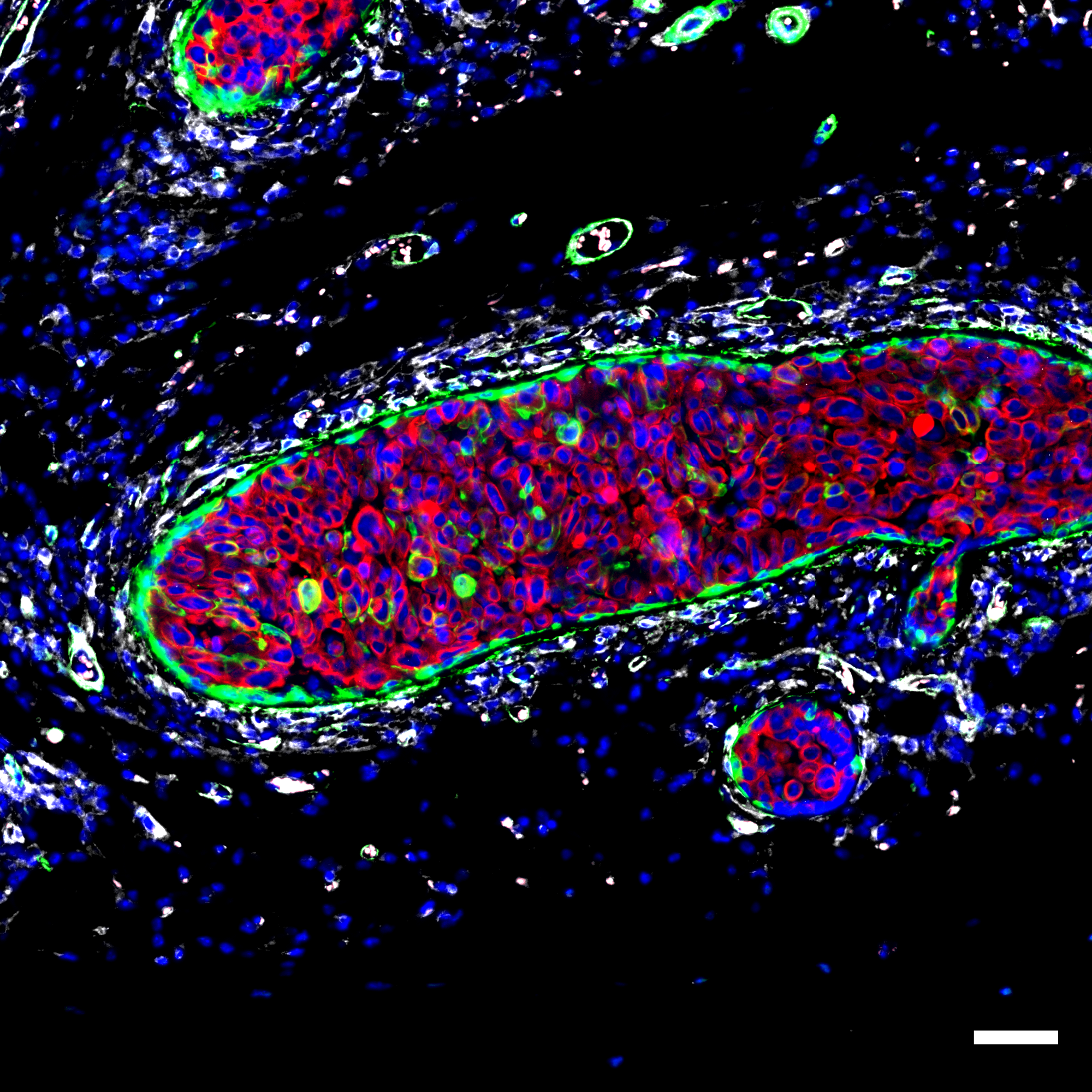

Supplement: Supplementary file 5 — Source data Fig. 3 [file 44319_2025_370_MOESM5_ESM.zip › Source Data Fig 3/3B/LKO K8 aSMA PDGFRB.tif]

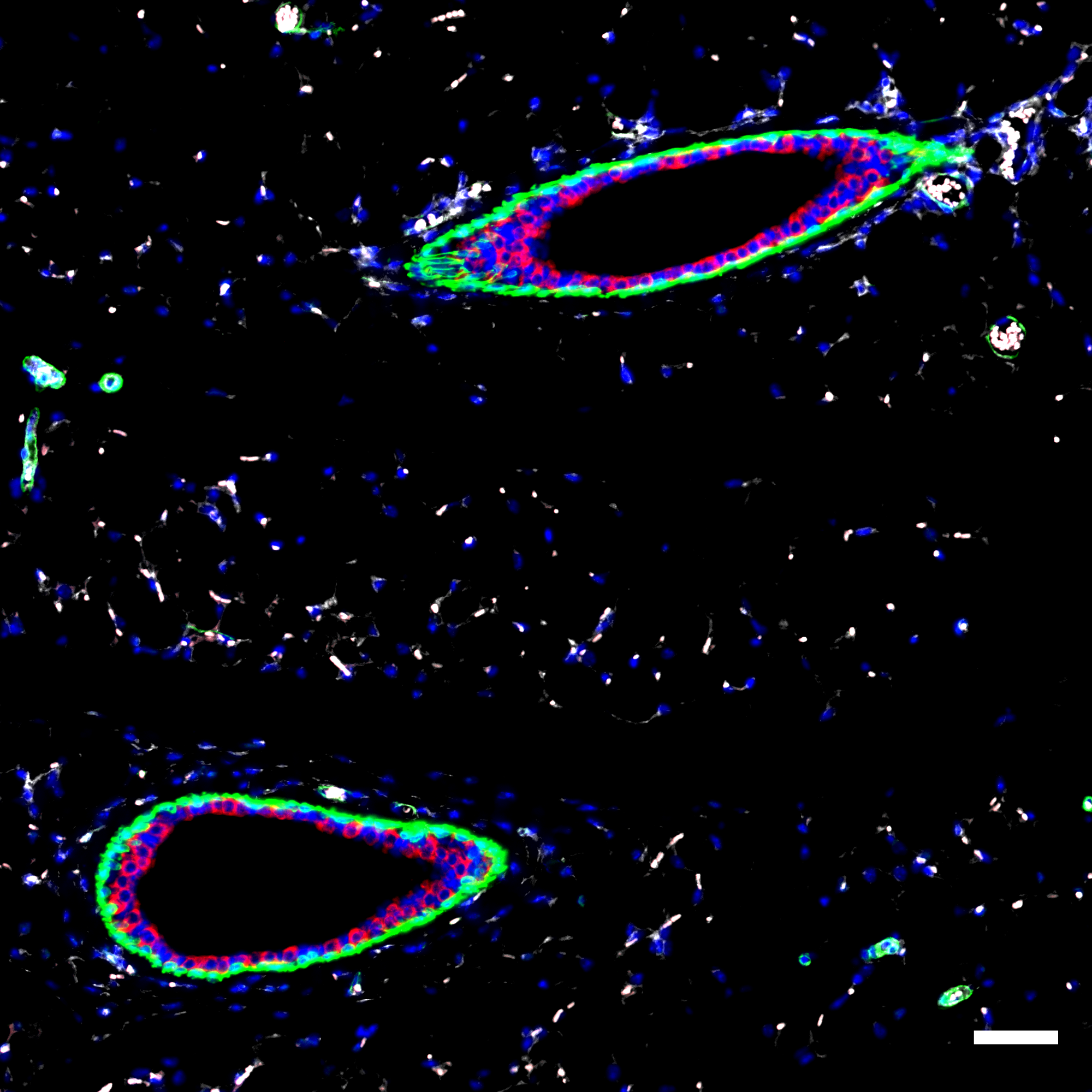

Supplement: Supplementary file 5 — Source data Fig. 3 [file 44319_2025_370_MOESM5_ESM.zip › Source Data Fig 3/3B/CTL K8 aSMA PDGFRB.tif]

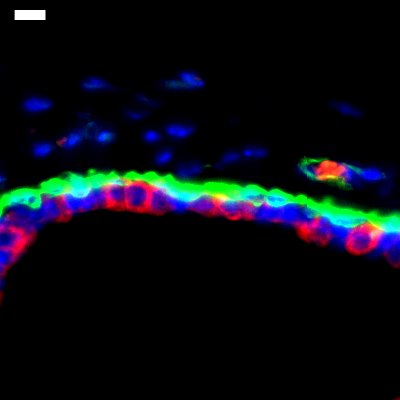

Supplement: Supplementary file 5 — Source data Fig. 3 [file 44319_2025_370_MOESM5_ESM.zip › Source Data Fig 3/3B/CTL Subset K8 aSMA.tif]

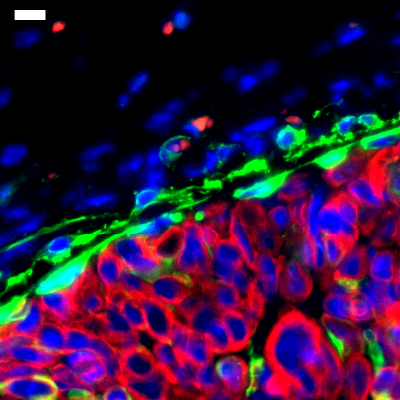

Supplement: Supplementary file 5 — Source data Fig. 3 [file 44319_2025_370_MOESM5_ESM.zip › Source Data Fig 3/3B/LKO Subset K8 aSMA.tif]

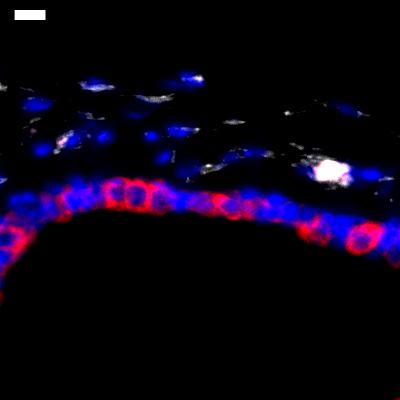

Supplement: Supplementary file 5 — Source data Fig. 3 [file 44319_2025_370_MOESM5_ESM.zip › Source Data Fig 3/3B/CTL Subset K8 PDGFRB.tif]

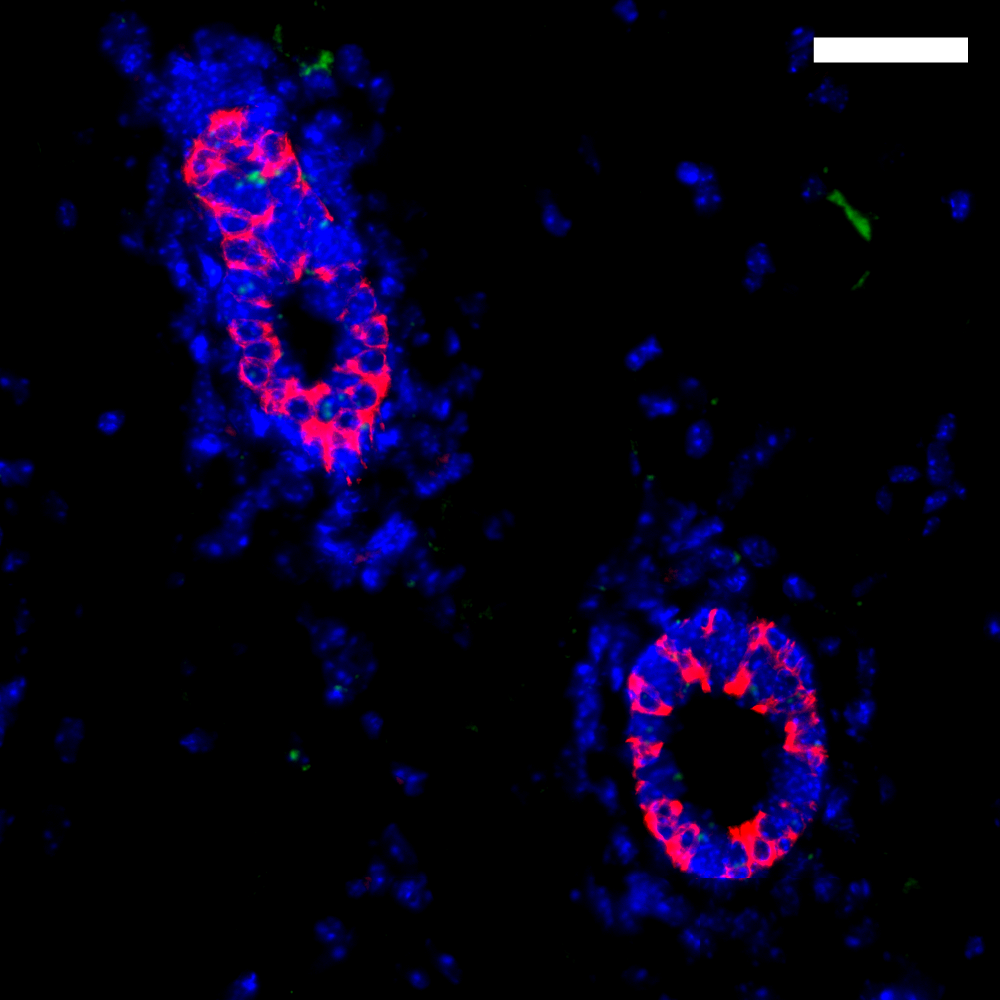

Supplement: Supplementary file 5 — Source data Fig. 3 [file 44319_2025_370_MOESM5_ESM.zip › Source Data Fig 3/3J/CTL K8 Tgfb2rna.tif]

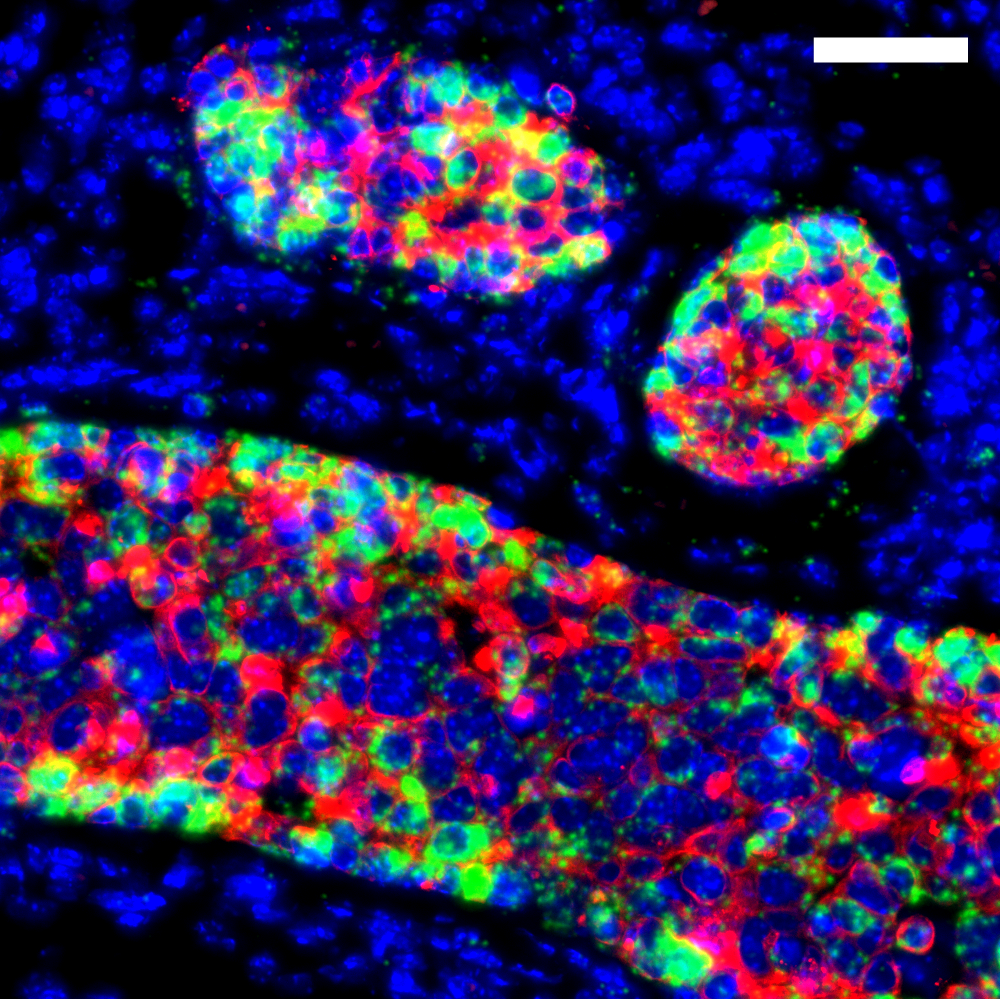

Supplement: Supplementary file 5 — Source data Fig. 3 [file 44319_2025_370_MOESM5_ESM.zip › Source Data Fig 3/3J/L12KO K8 Tgfb2rna.tif]

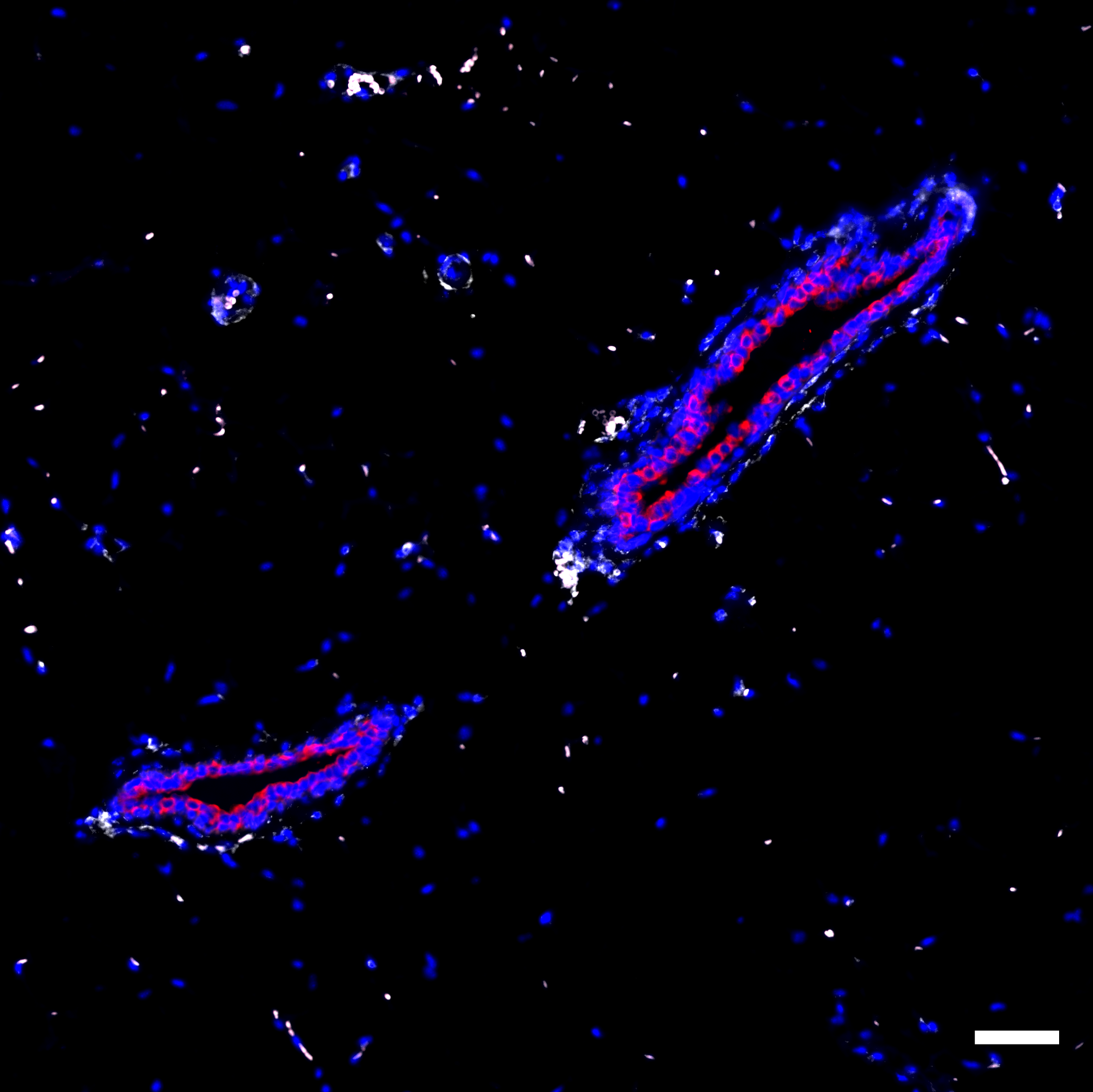

Supplement: Supplementary file 5 — Source data Fig. 3 [file 44319_2025_370_MOESM5_ESM.zip › Source Data Fig 3/3A/CTL K14 K8 PDGFRB.tif]

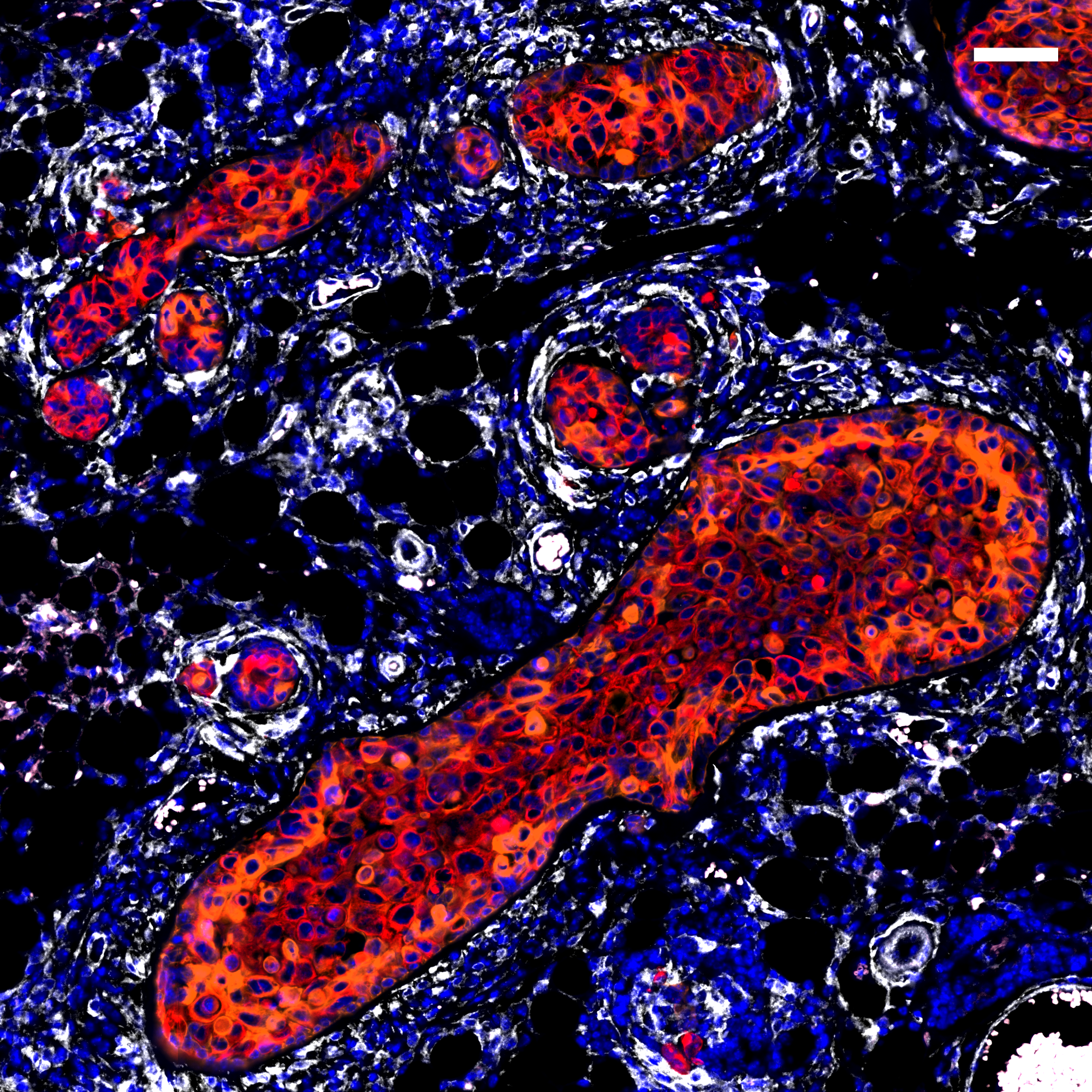

Supplement: Supplementary file 5 — Source data Fig. 3 [file 44319_2025_370_MOESM5_ESM.zip › Source Data Fig 3/3A/L12KO K14 K8 PDGFRB.tif]

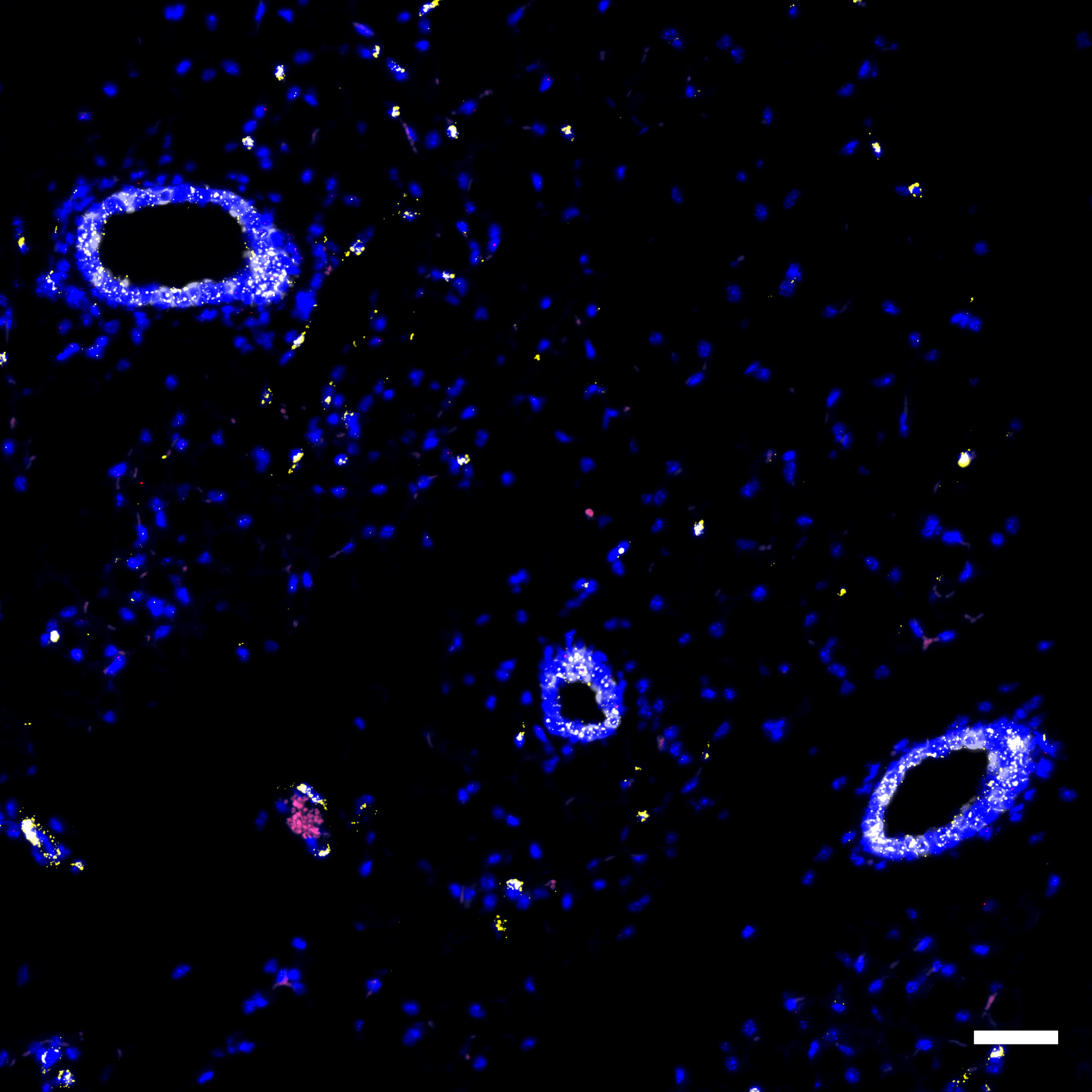

Supplement: Supplementary file 5 — Source data Fig. 3 [file 44319_2025_370_MOESM5_ESM.zip › Source Data Fig 3/3F/CTL K8 Lrrc15rna Notch3rna.tif]

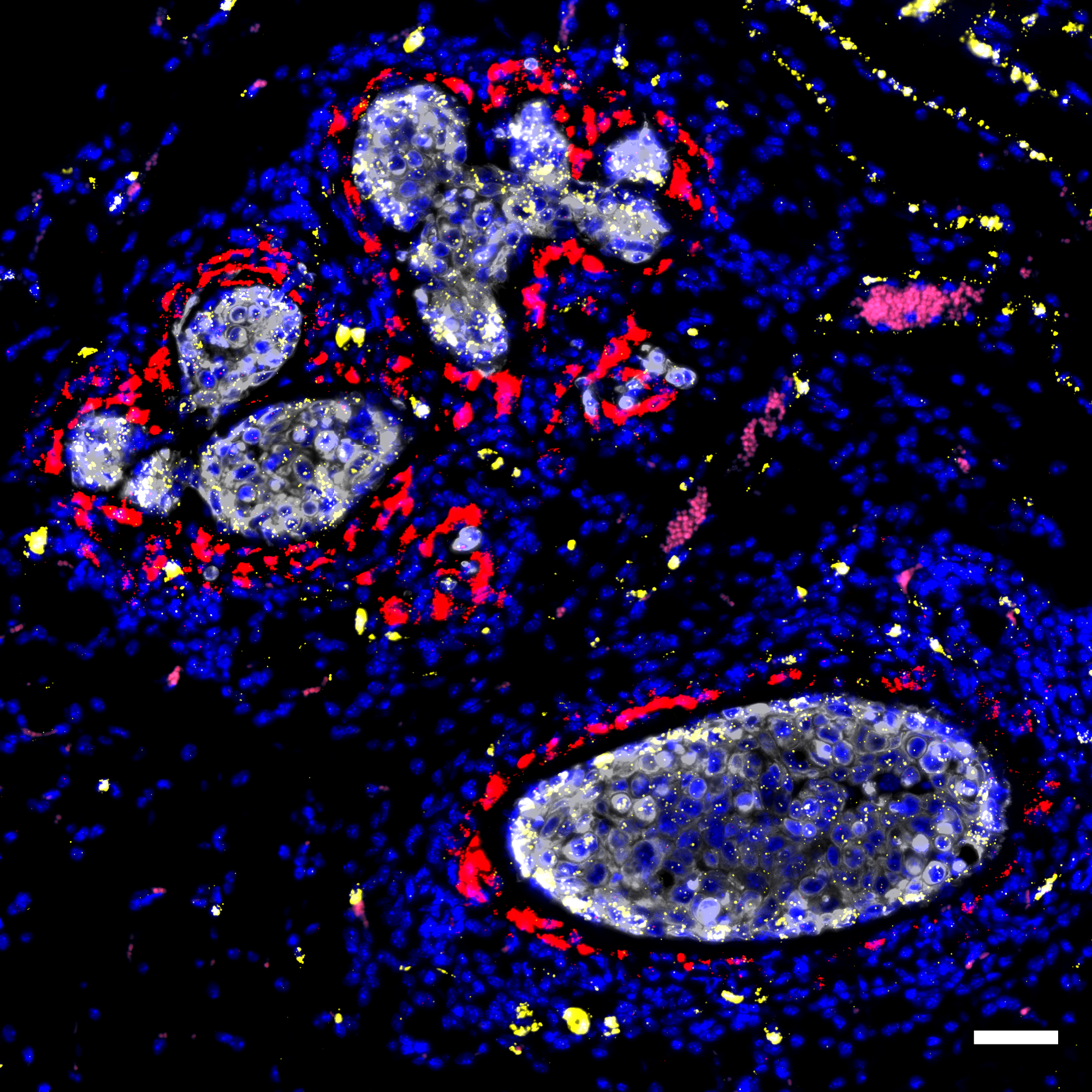

Supplement: Supplementary file 5 — Source data Fig. 3 [file 44319_2025_370_MOESM5_ESM.zip › Source Data Fig 3/3F/L12KO K8 Lrrc15rna Notch3rna.tif]

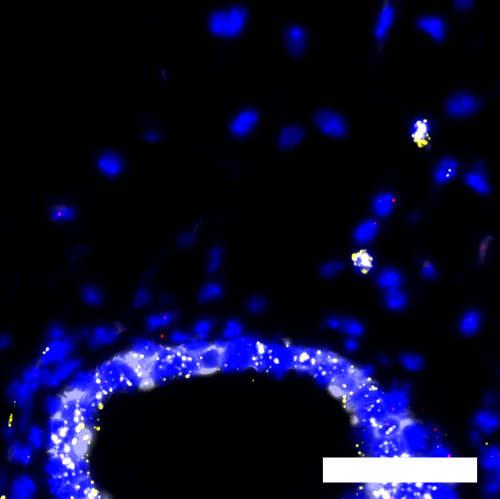

Supplement: Supplementary file 5 — Source data Fig. 3 [file 44319_2025_370_MOESM5_ESM.zip › Source Data Fig 3/3F/CTL Subset K8 Lrrc15rna Notch3rna.tif]

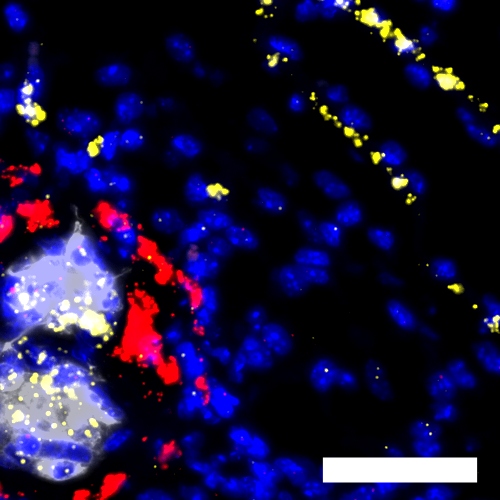

Supplement: Supplementary file 5 — Source data Fig. 3 [file 44319_2025_370_MOESM5_ESM.zip › Source Data Fig 3/3F/L12KO Subset K8 Lrrc15rna Notch3rna.tif]

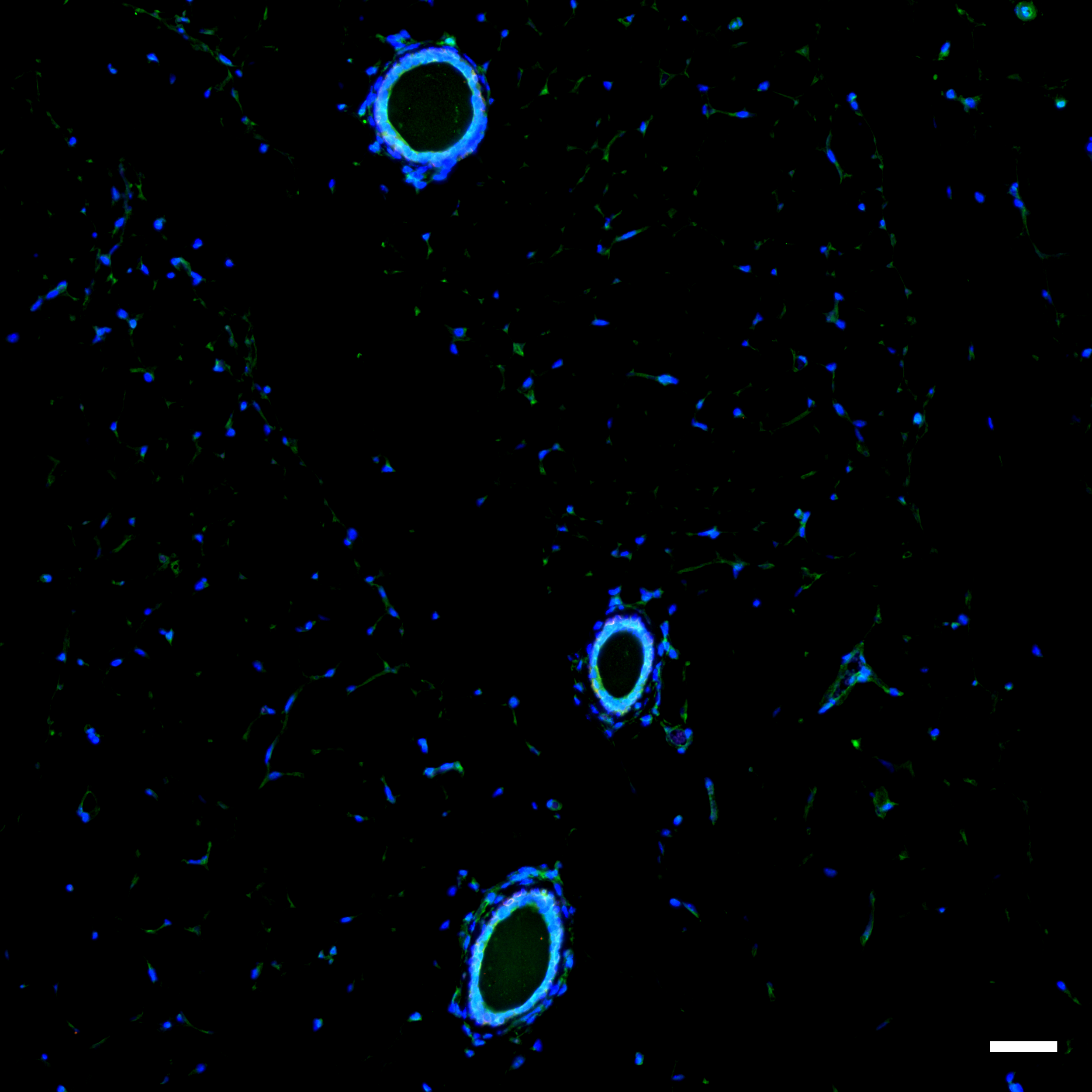

Supplement: Supplementary file 6 — Source data Fig. 4 [file 44319_2025_370_MOESM6_ESM.zip › Source Data Fig 4/4K/CTL K14 K8 FN.tif]

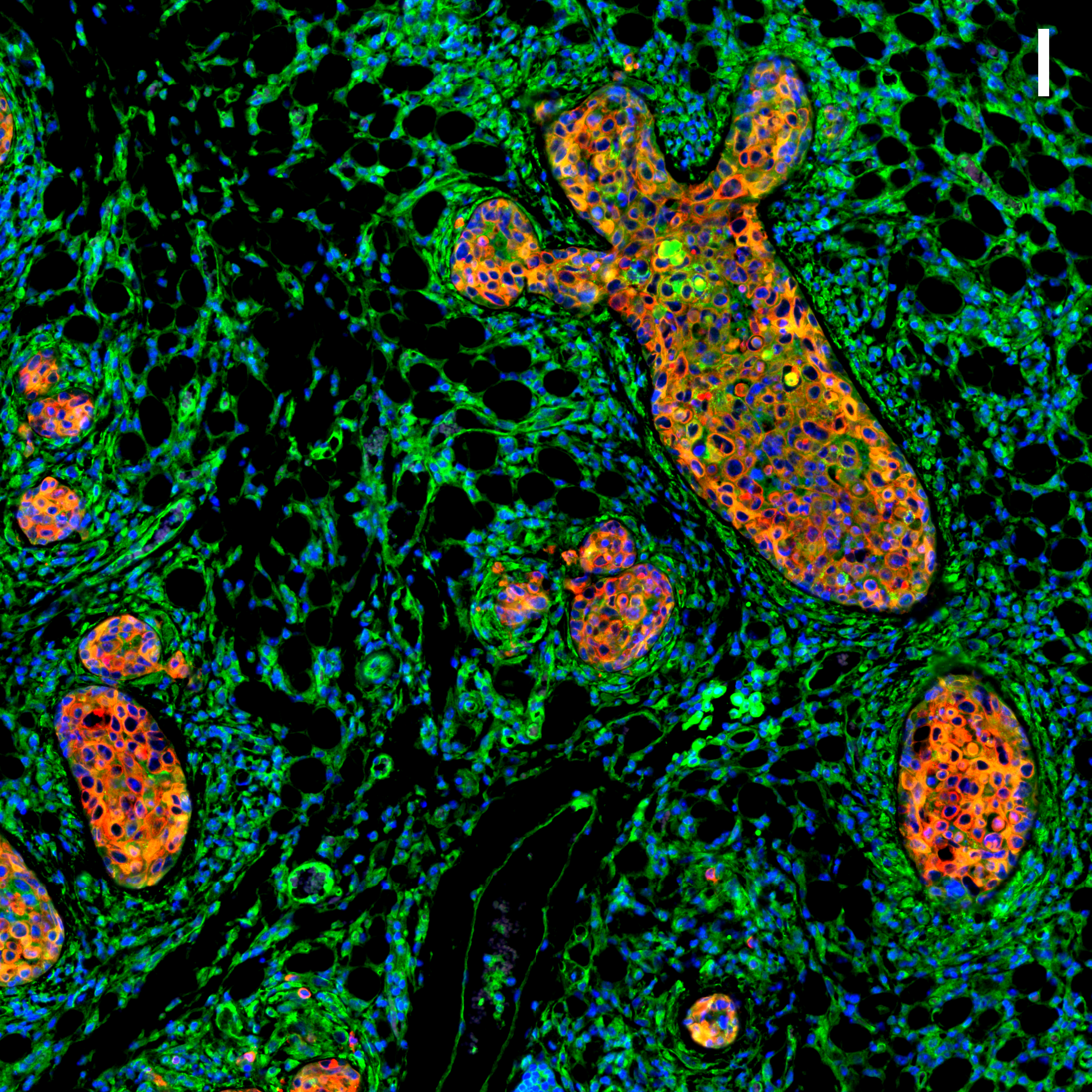

Supplement: Supplementary file 6 — Source data Fig. 4 [file 44319_2025_370_MOESM6_ESM.zip › Source Data Fig 4/4K/L12KO K14 K8 FN.tif]

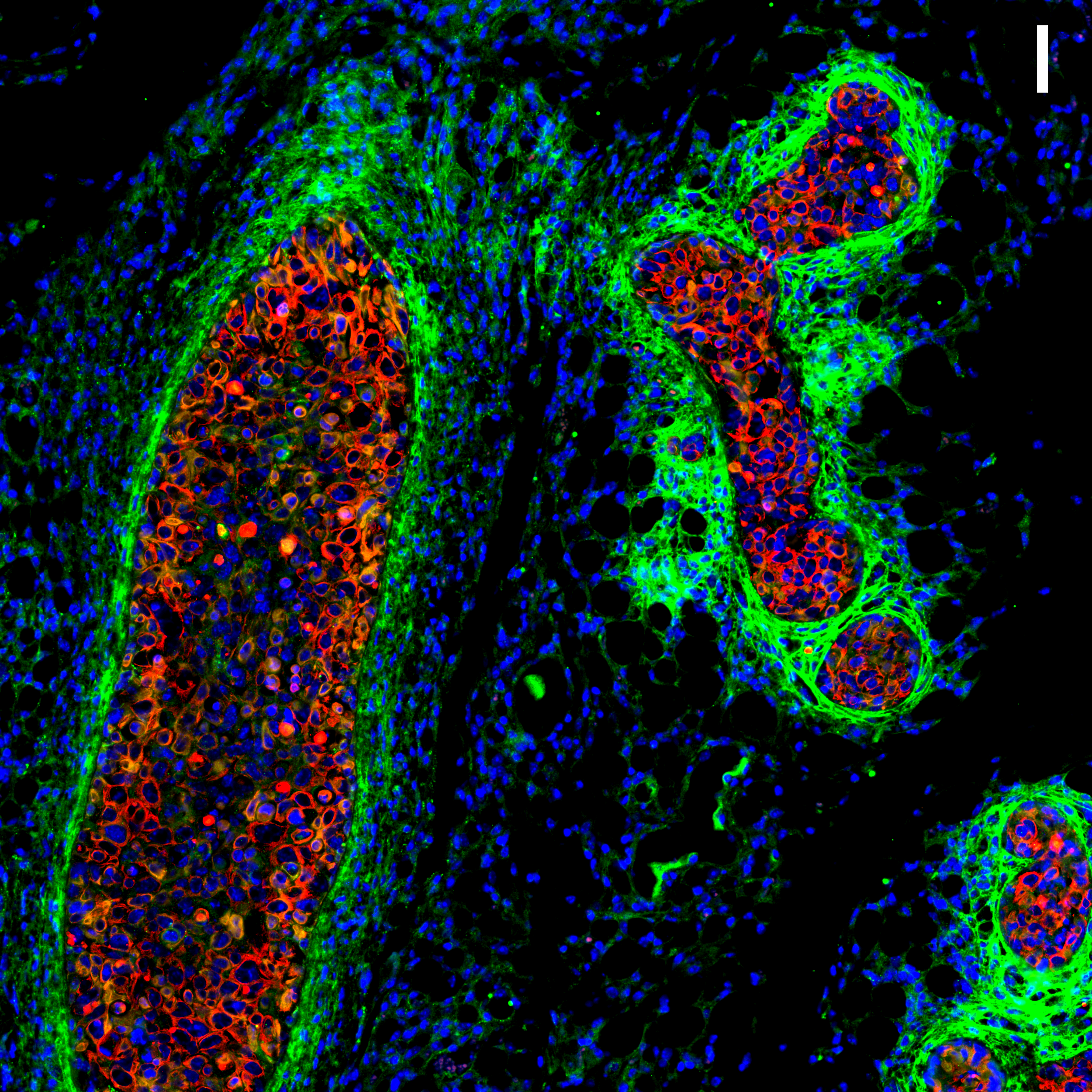

Supplement: Supplementary file 6 — Source data Fig. 4 [file 44319_2025_370_MOESM6_ESM.zip › Source Data Fig 4/4L/L12KO K14 K8 POSTN.tif]

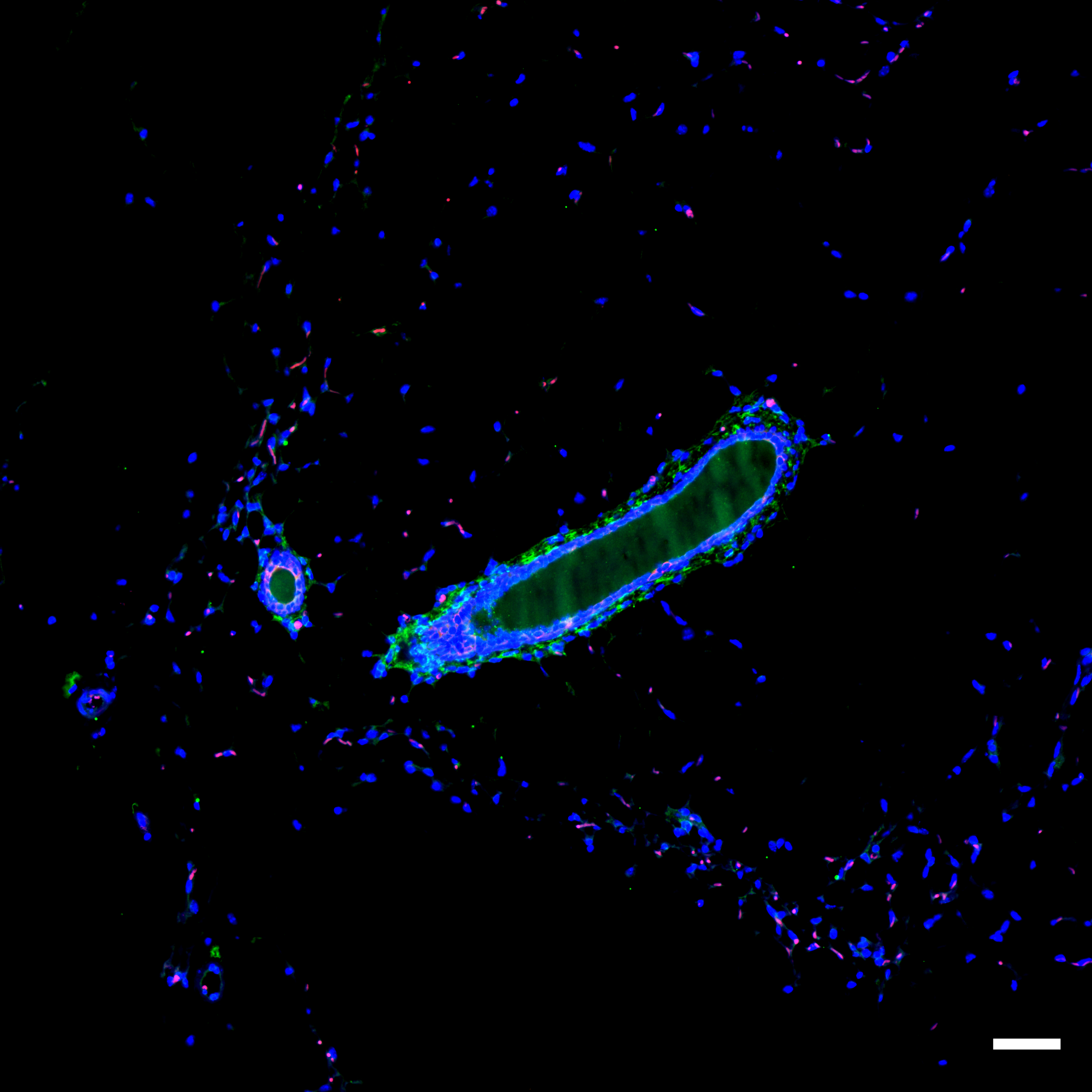

Supplement: Supplementary file 6 — Source data Fig. 4 [file 44319_2025_370_MOESM6_ESM.zip › Source Data Fig 4/4L/CTL K14 K8 POSTN.tif]

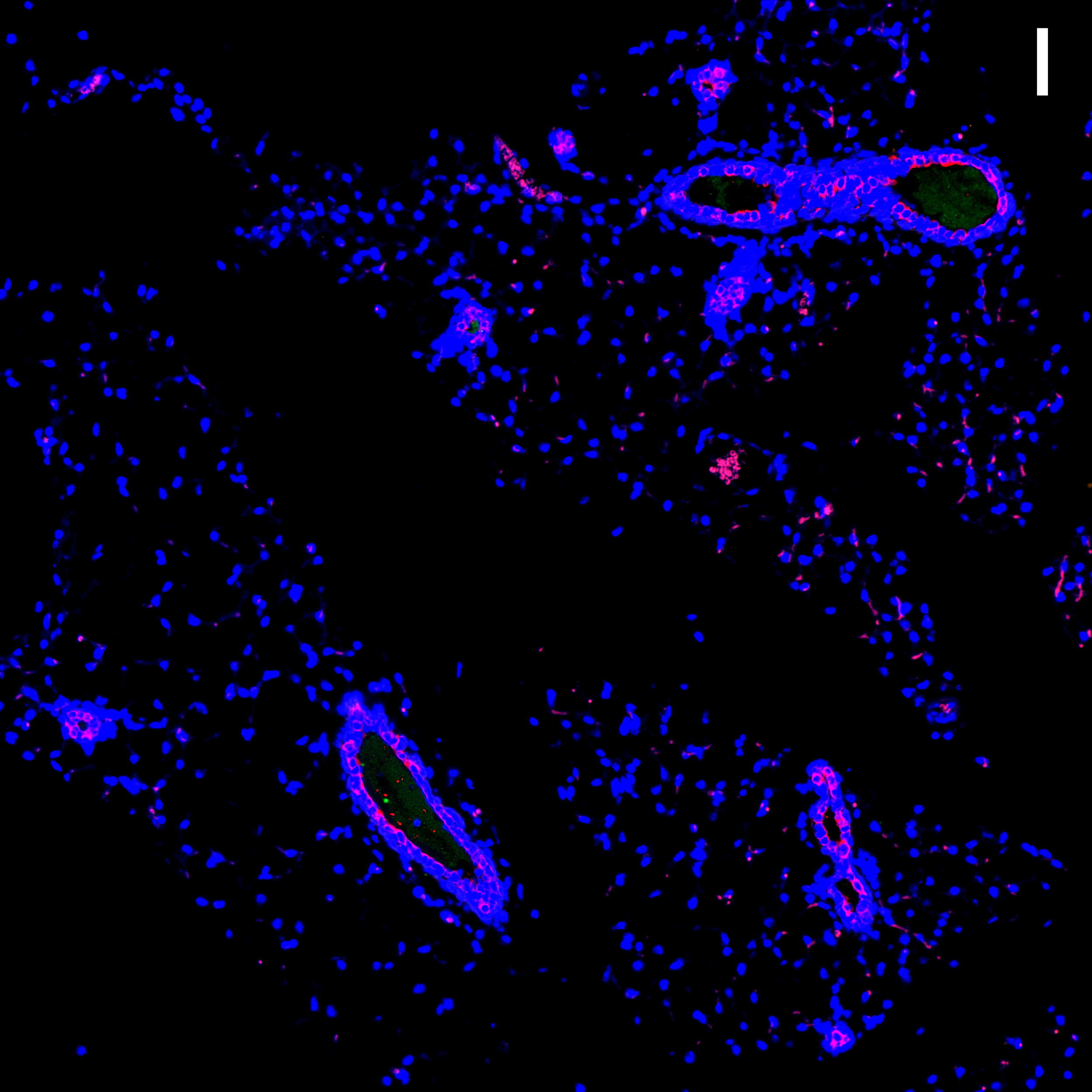

Supplement: Supplementary file 6 — Source data Fig. 4 [file 44319_2025_370_MOESM6_ESM.zip › Source Data Fig 4/4M/CTL K14 K8 TNC.tif]

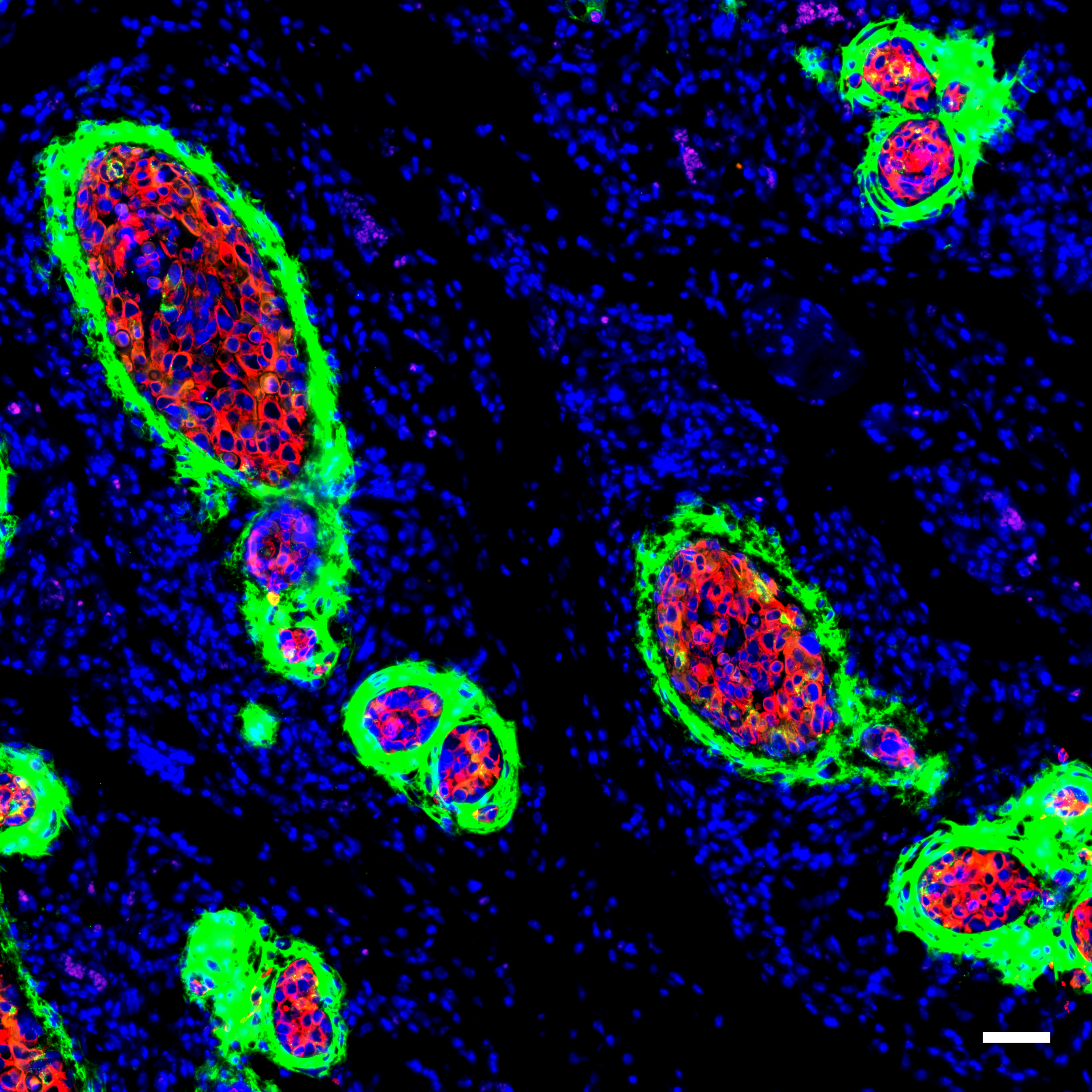

Supplement: Supplementary file 6 — Source data Fig. 4 [file 44319_2025_370_MOESM6_ESM.zip › Source Data Fig 4/4M/L12KO K14 K8 TNC.tif]

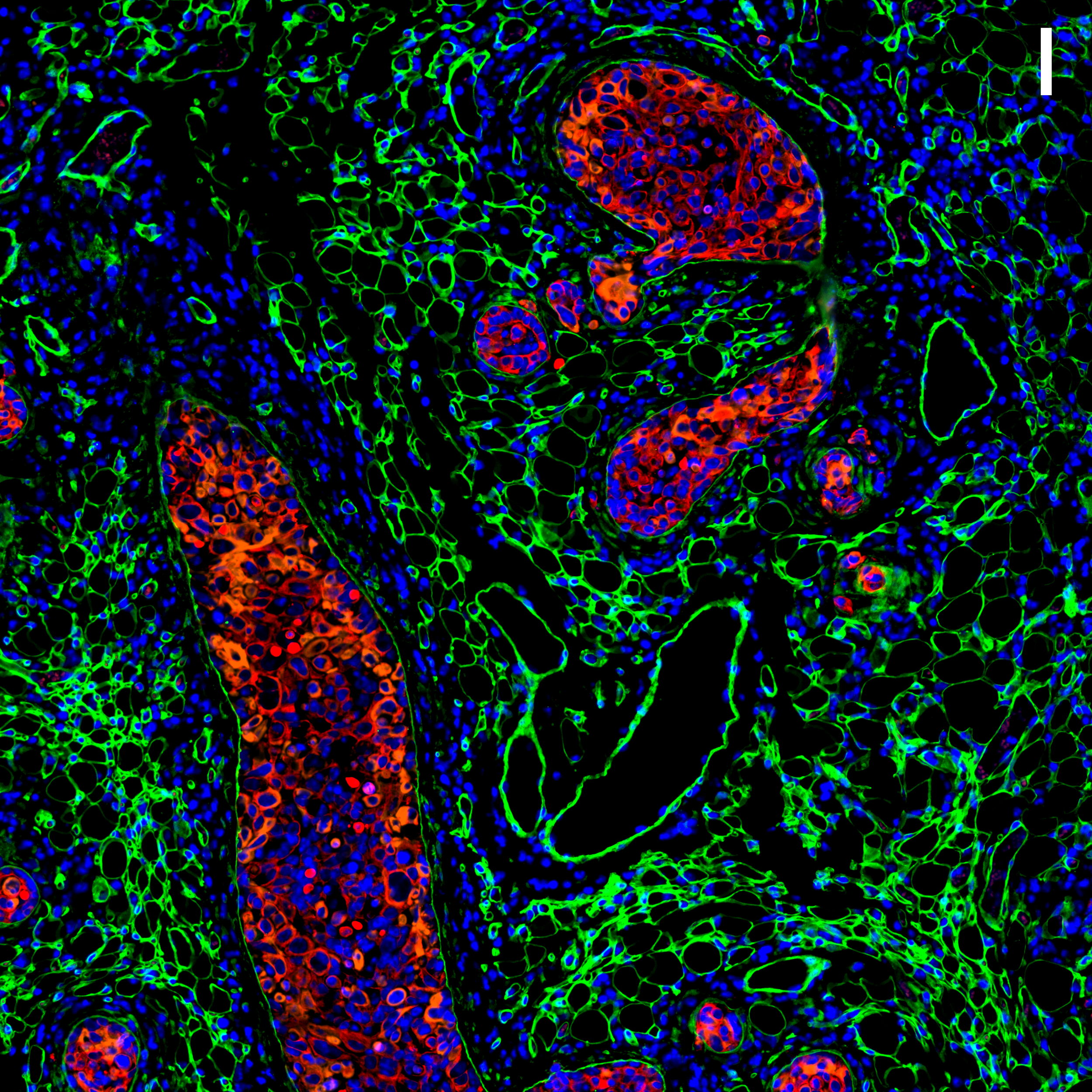

Supplement: Supplementary file 6 — Source data Fig. 4 [file 44319_2025_370_MOESM6_ESM.zip › Source Data Fig 4/4J/L12KO K14 K8 LAMC1.tif]

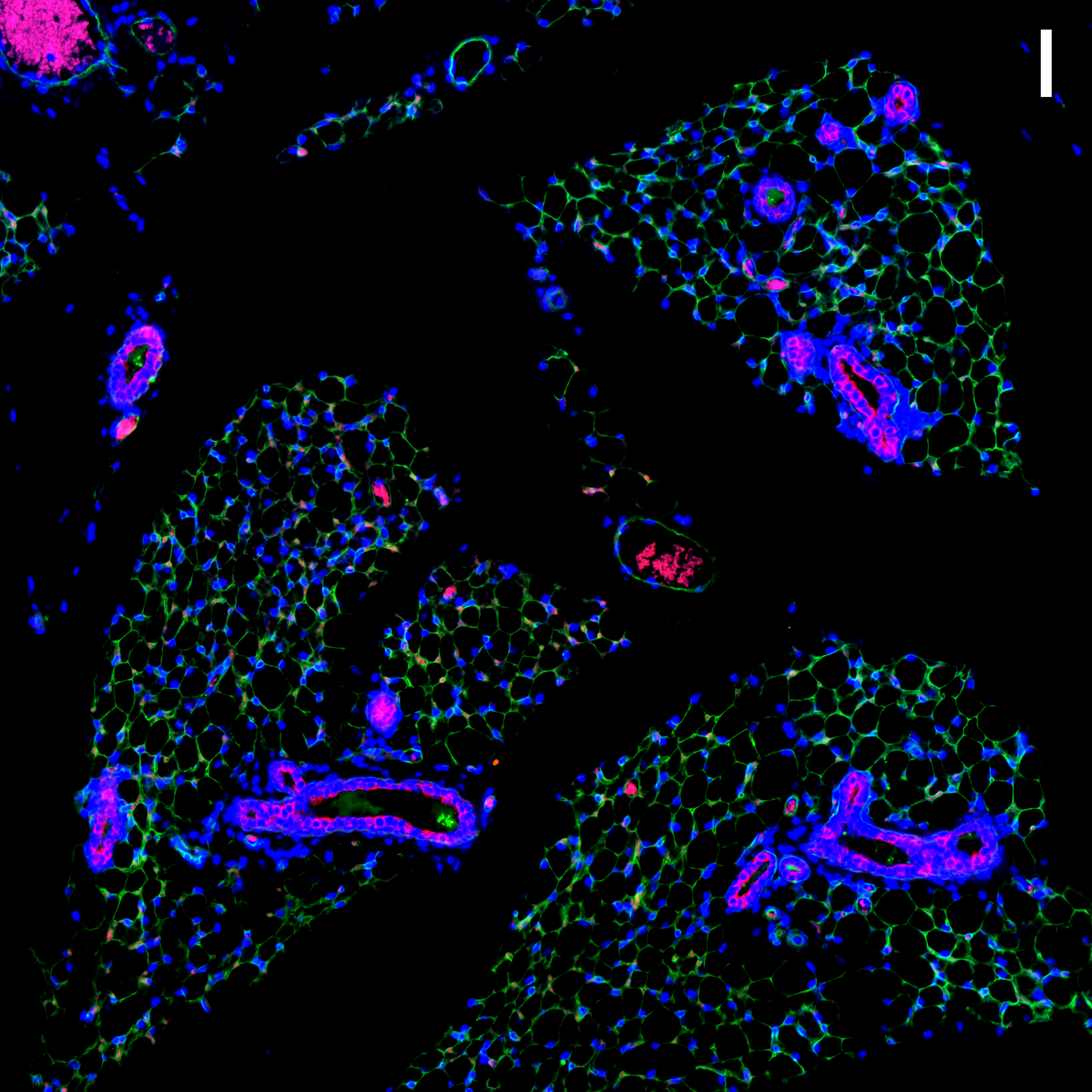

Supplement: Supplementary file 6 — Source data Fig. 4 [file 44319_2025_370_MOESM6_ESM.zip › Source Data Fig 4/4J/CTL K14 K8 LAMC1.tif]

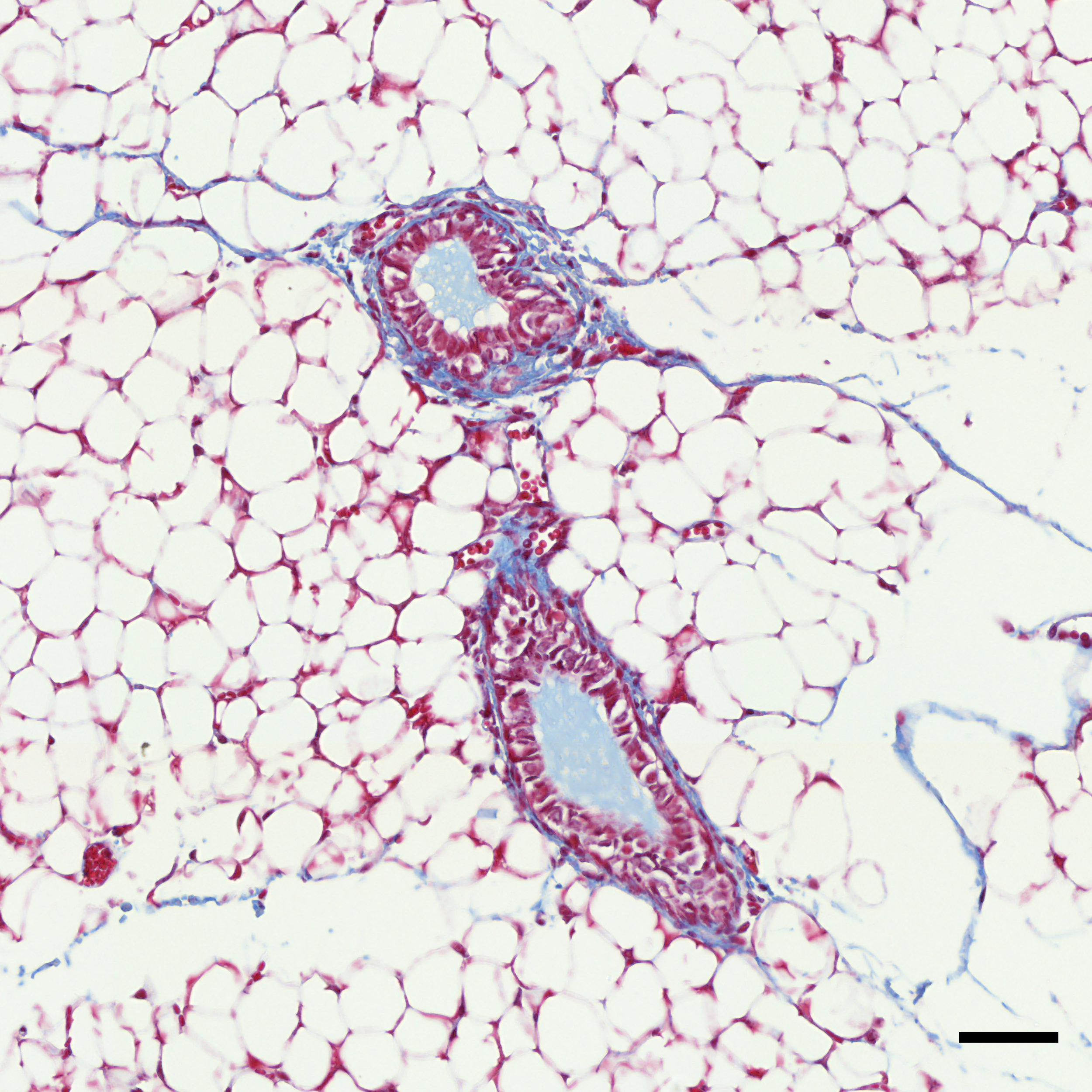

Supplement: Supplementary file 6 — Source data Fig. 4 [file 44319_2025_370_MOESM6_ESM.zip › Source Data Fig 4/4F/CTL Masson-Trichrome.tif]

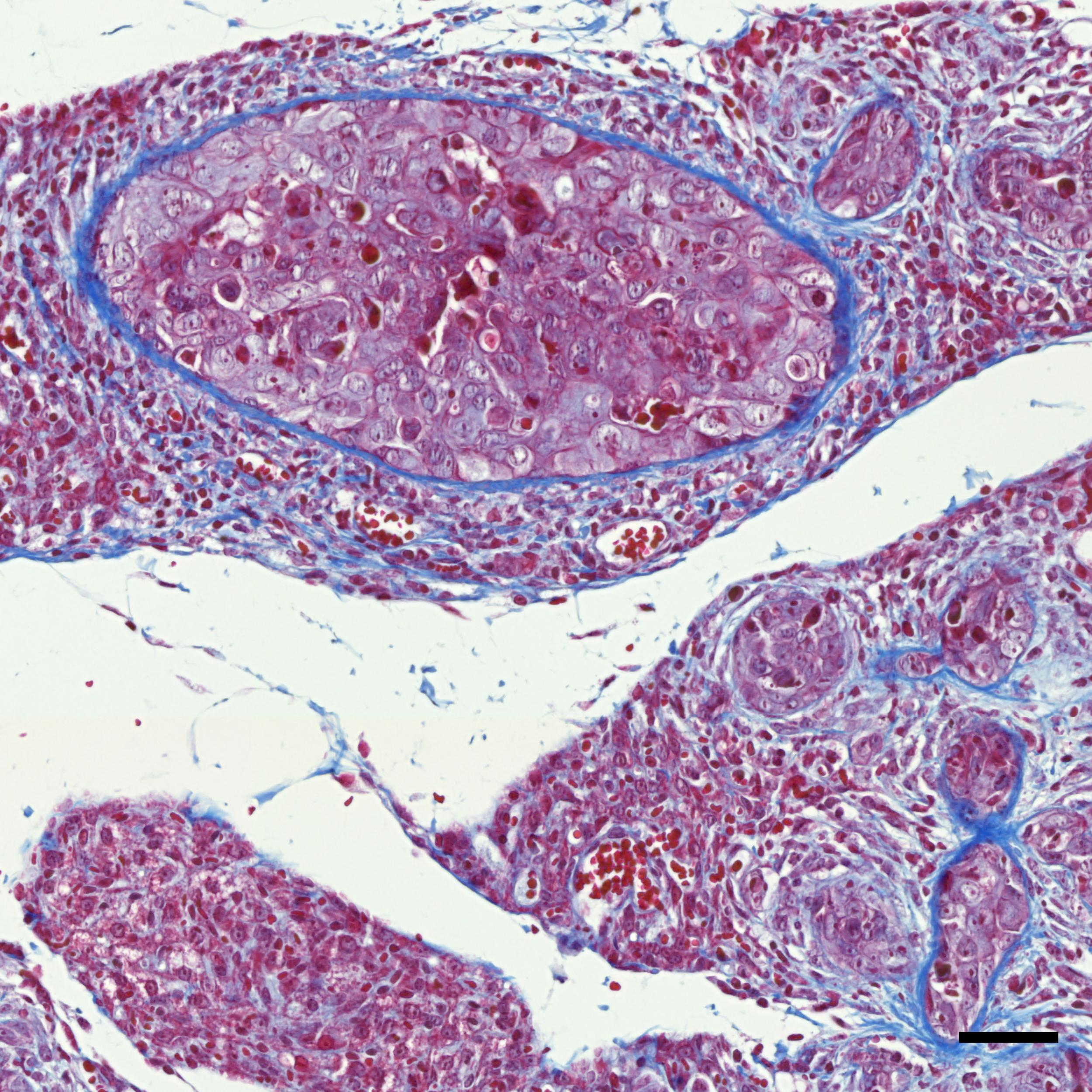

Supplement: Supplementary file 6 — Source data Fig. 4 [file 44319_2025_370_MOESM6_ESM.zip › Source Data Fig 4/4F/L12KO Masson-Trichrome.tif]

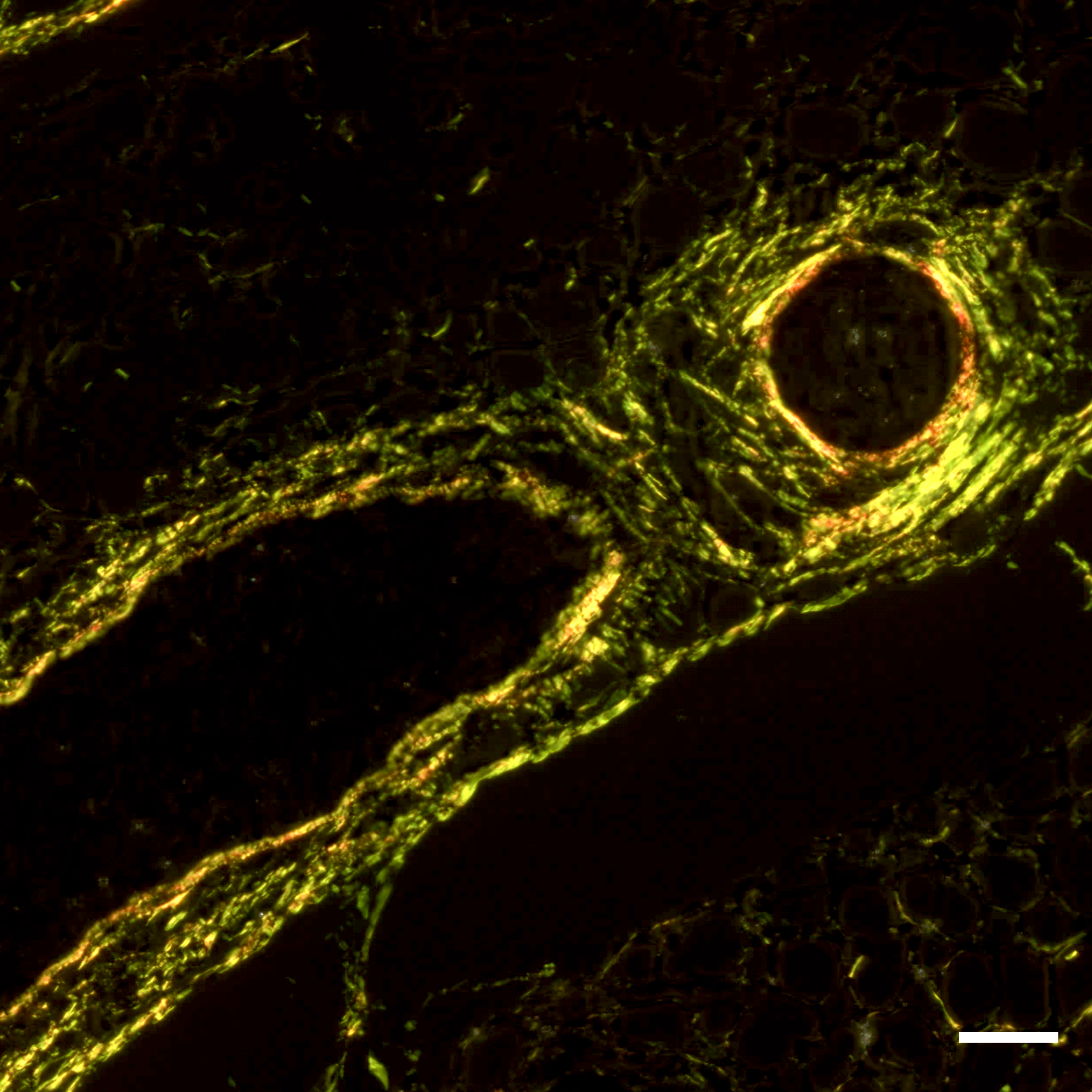

Supplement: Supplementary file 6 — Source data Fig. 4 [file 44319_2025_370_MOESM6_ESM.zip › Source Data Fig 4/4H/L12KO Picrosirius Polarized.tif]

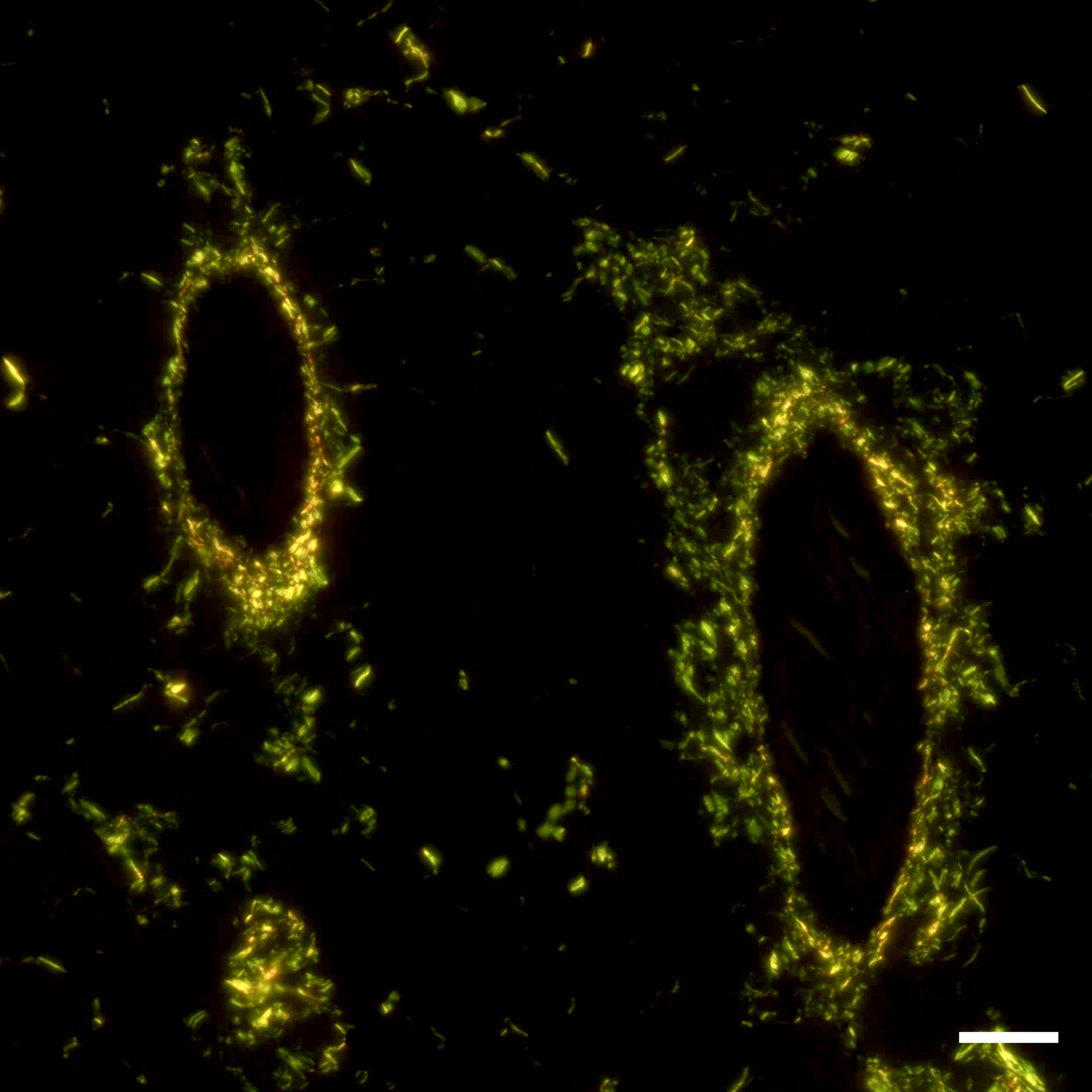

Supplement: Supplementary file 6 — Source data Fig. 4 [file 44319_2025_370_MOESM6_ESM.zip › Source Data Fig 4/4H/CTL Picrosirius Polarized.tif]

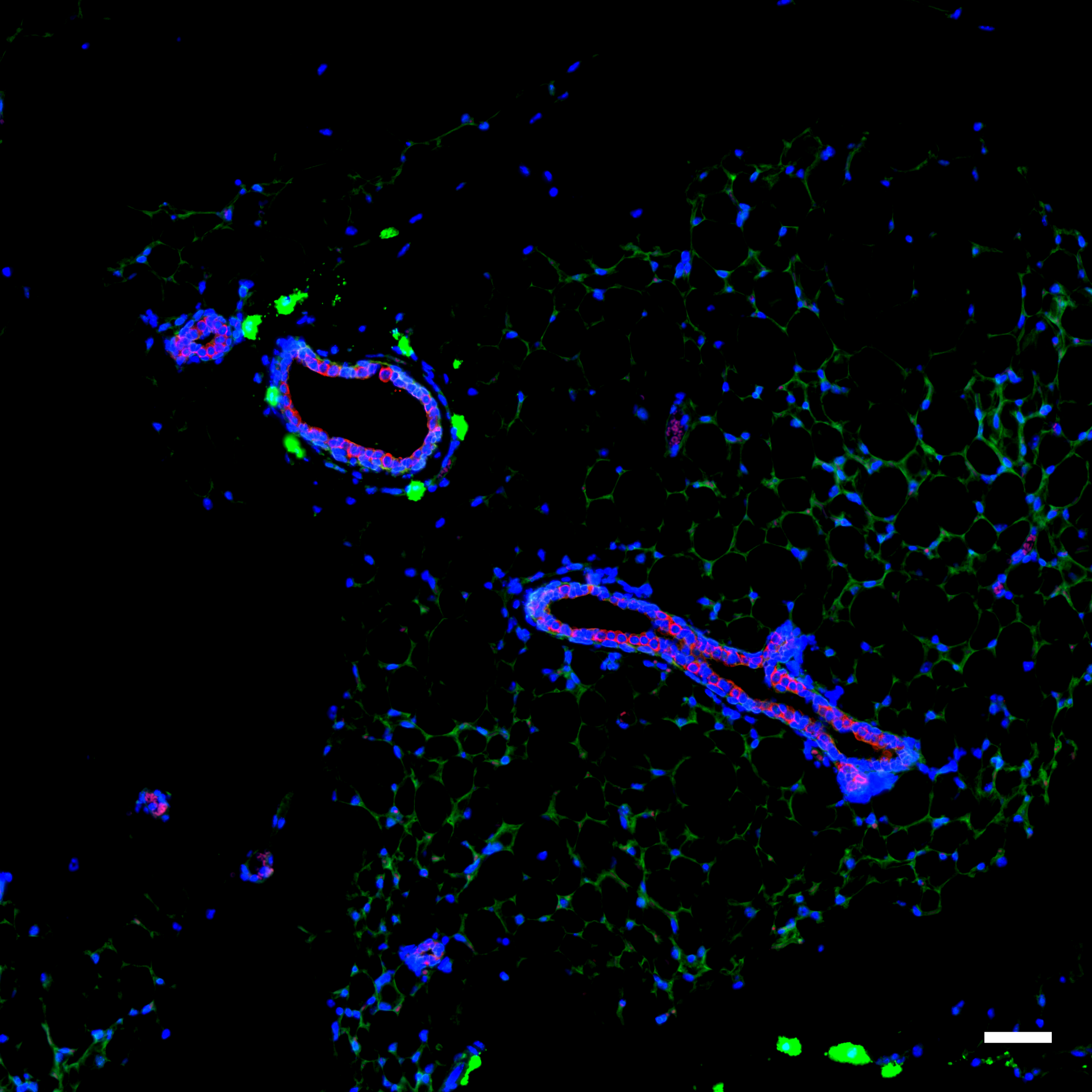

Supplement: Supplementary file 6 — Source data Fig. 4 [file 44319_2025_370_MOESM6_ESM.zip › Source Data Fig 4/4I/CTL K14 K8 LAMB1.tif]

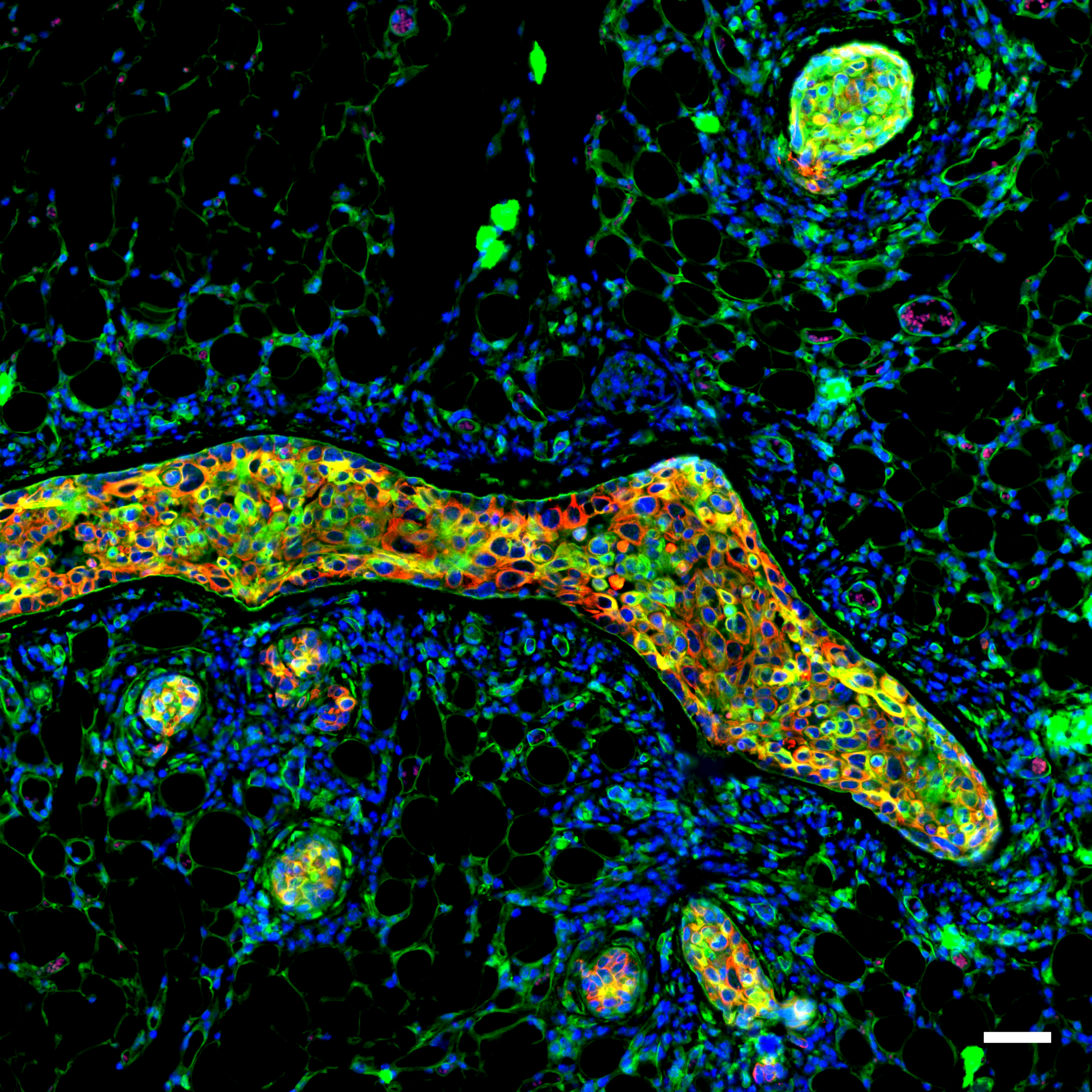

Supplement: Supplementary file 6 — Source data Fig. 4 [file 44319_2025_370_MOESM6_ESM.zip › Source Data Fig 4/4I/L12KO K14 K8 LAMB1.tif]

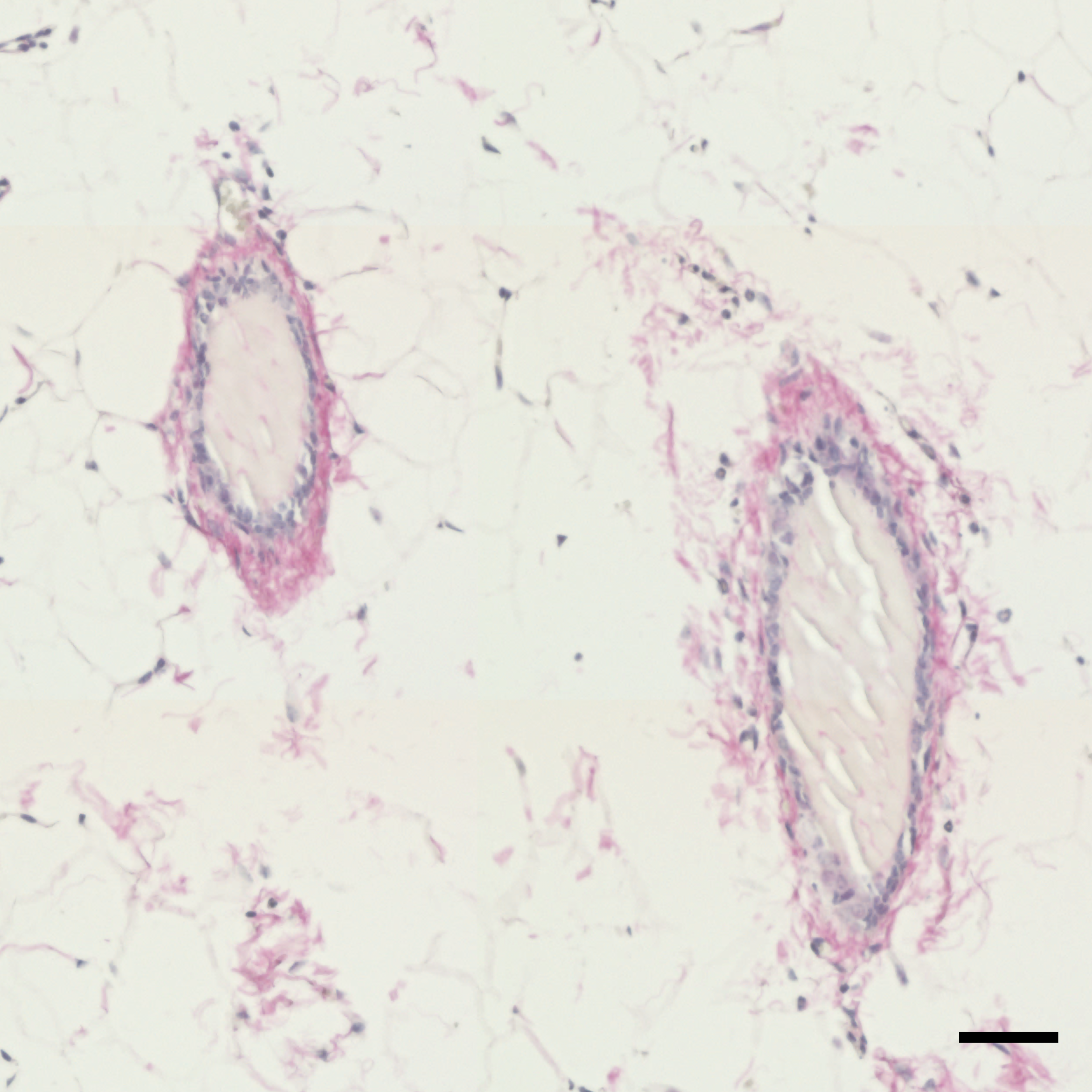

Supplement: Supplementary file 6 — Source data Fig. 4 [file 44319_2025_370_MOESM6_ESM.zip › Source Data Fig 4/4G/CTL Picrosirius Red.tif]

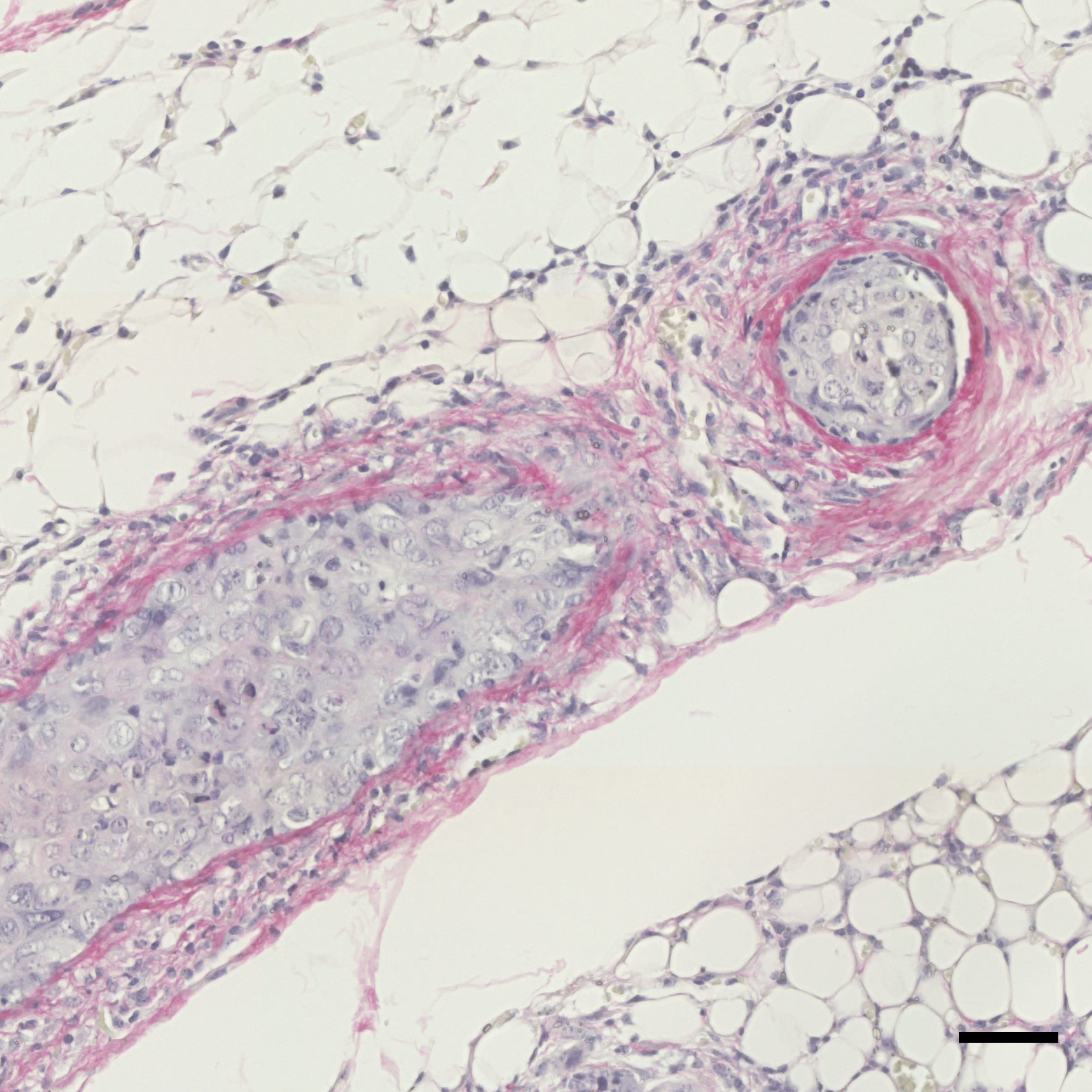

Supplement: Supplementary file 6 — Source data Fig. 4 [file 44319_2025_370_MOESM6_ESM.zip › Source Data Fig 4/4G/L12KO Picrosirius Red.tif]

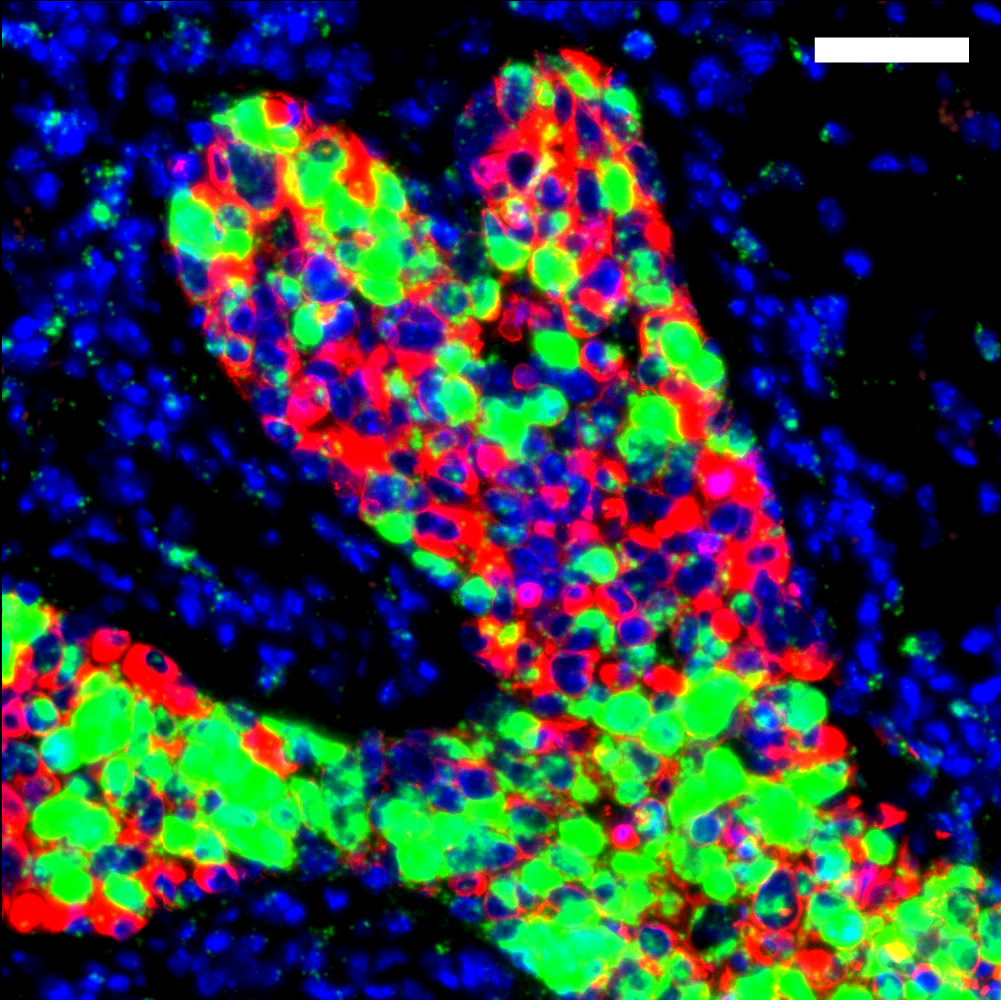

Supplement: Supplementary file 7 — Source data Fig. 5 [file 44319_2025_370_MOESM7_ESM.zip › Source Data Fig 5/5I/L12KO K8 Csf1rna.tif]

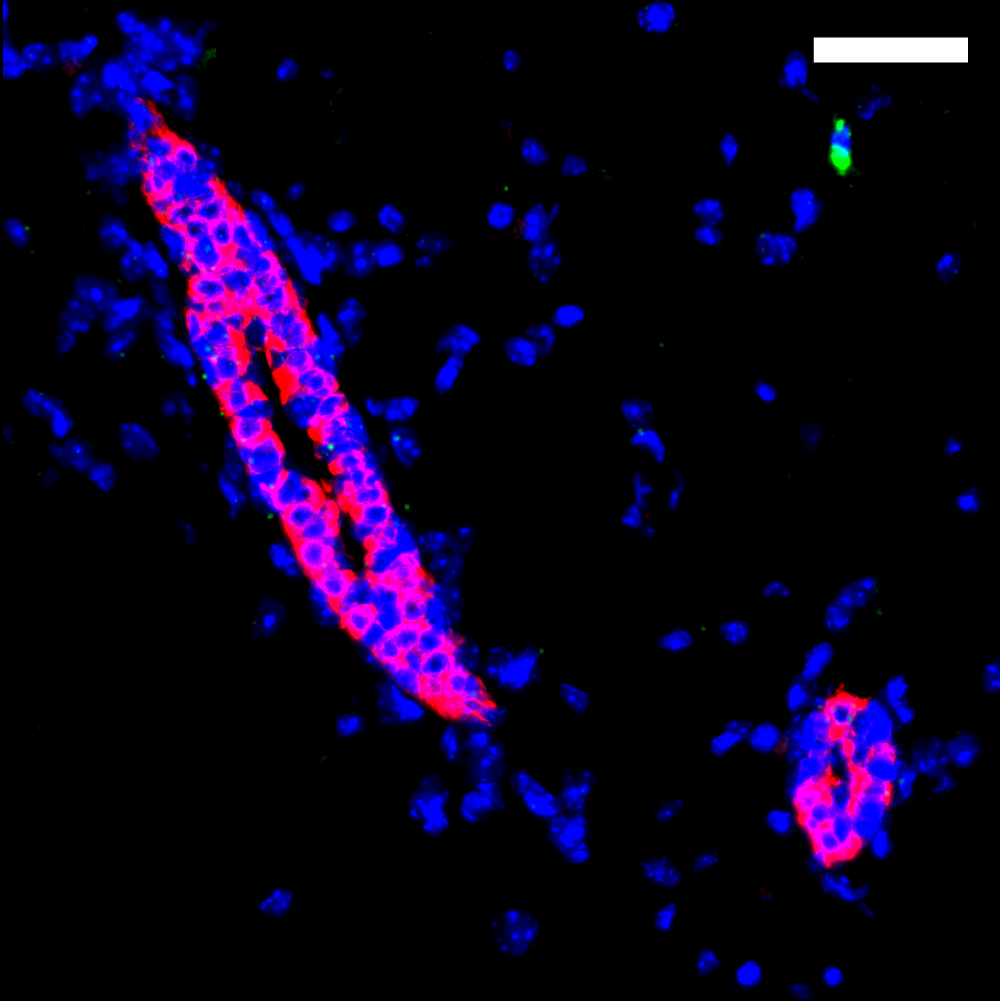

Supplement: Supplementary file 7 — Source data Fig. 5 [file 44319_2025_370_MOESM7_ESM.zip › Source Data Fig 5/5I/CTL K8 Csf1rna.tif]

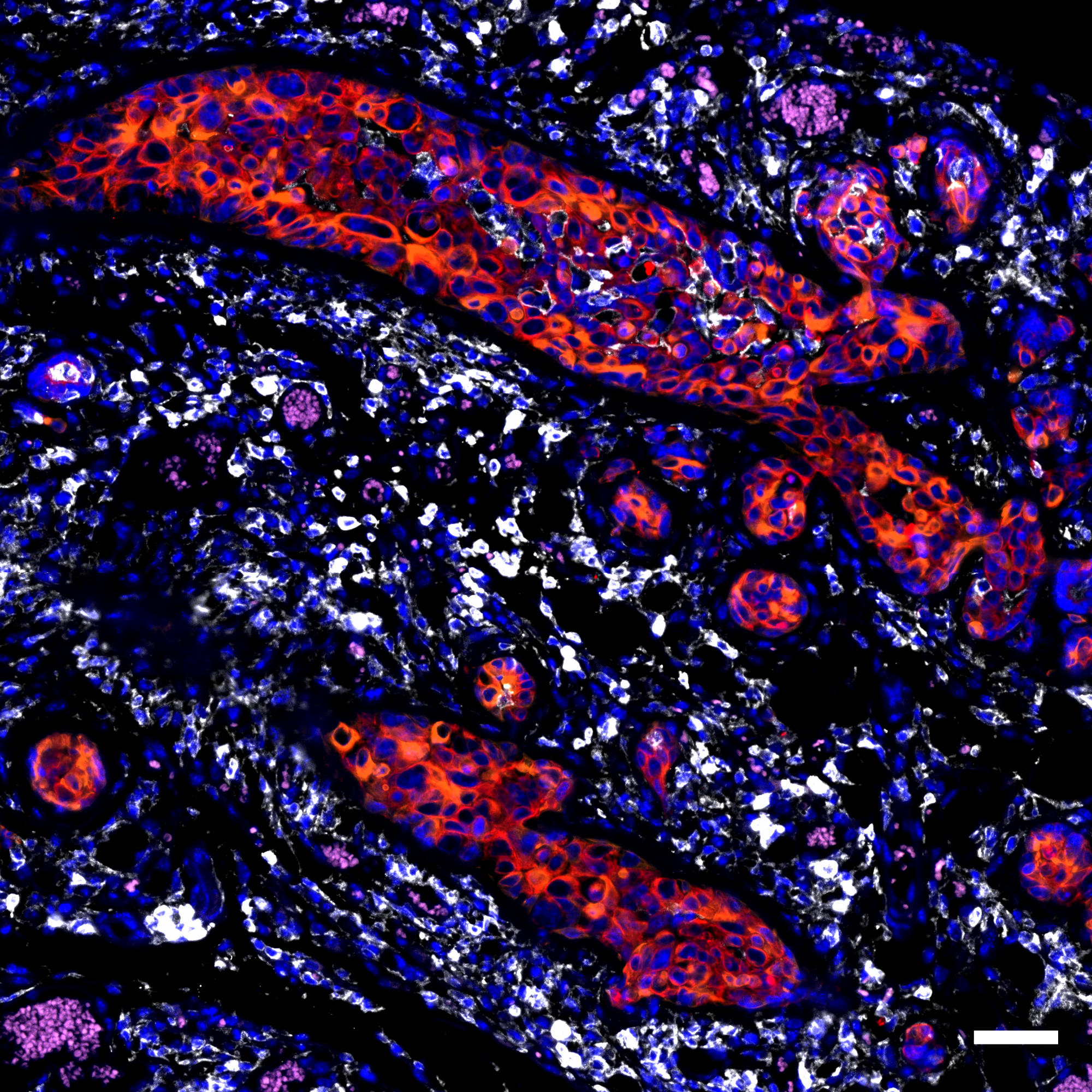

Supplement: Supplementary file 7 — Source data Fig. 5 [file 44319_2025_370_MOESM7_ESM.zip › Source Data Fig 5/5A/L12KO K14 K8 CD45.tif]

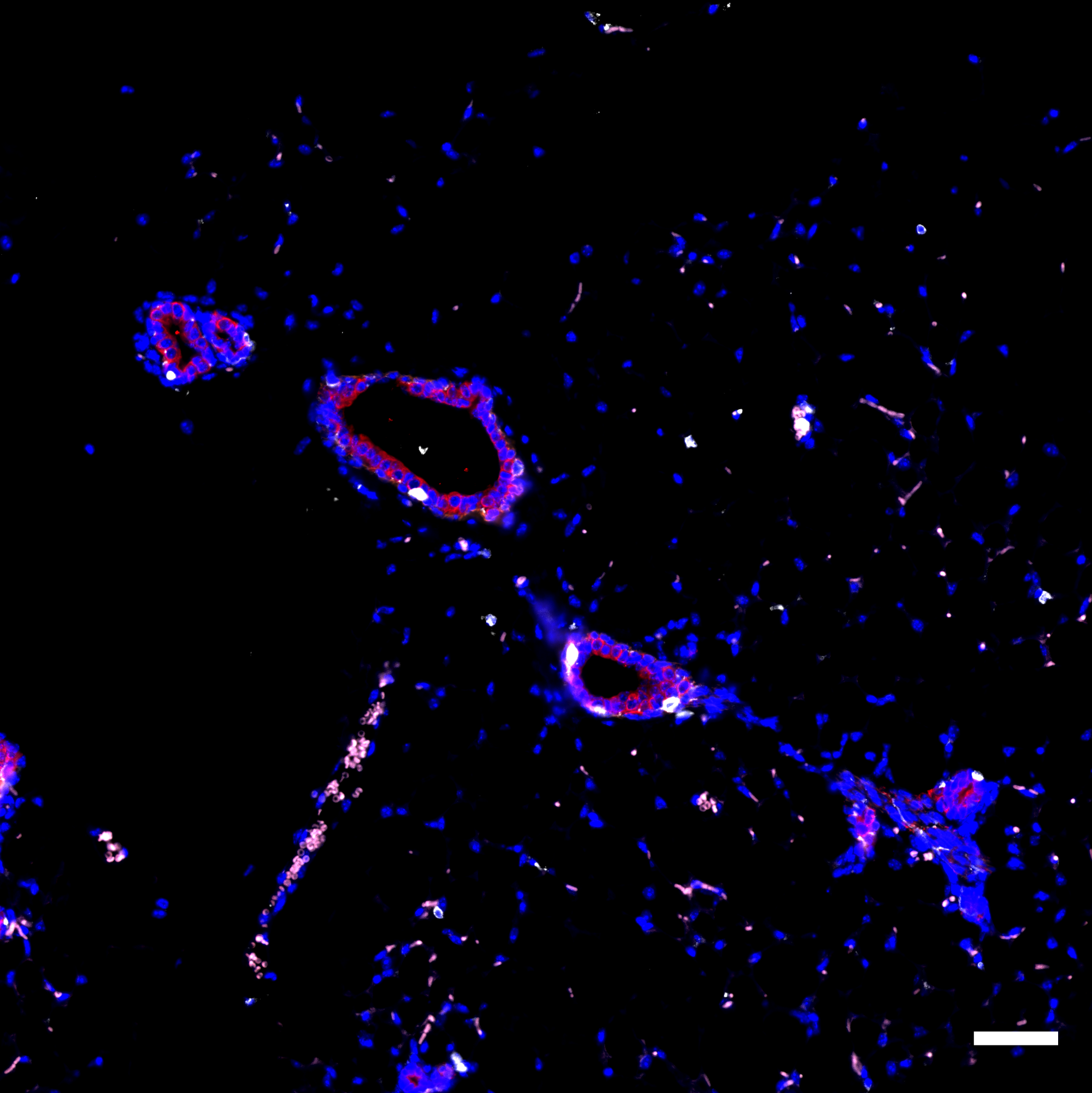

Supplement: Supplementary file 7 — Source data Fig. 5 [file 44319_2025_370_MOESM7_ESM.zip › Source Data Fig 5/5A/CTL K14 K8 CD45.tif]

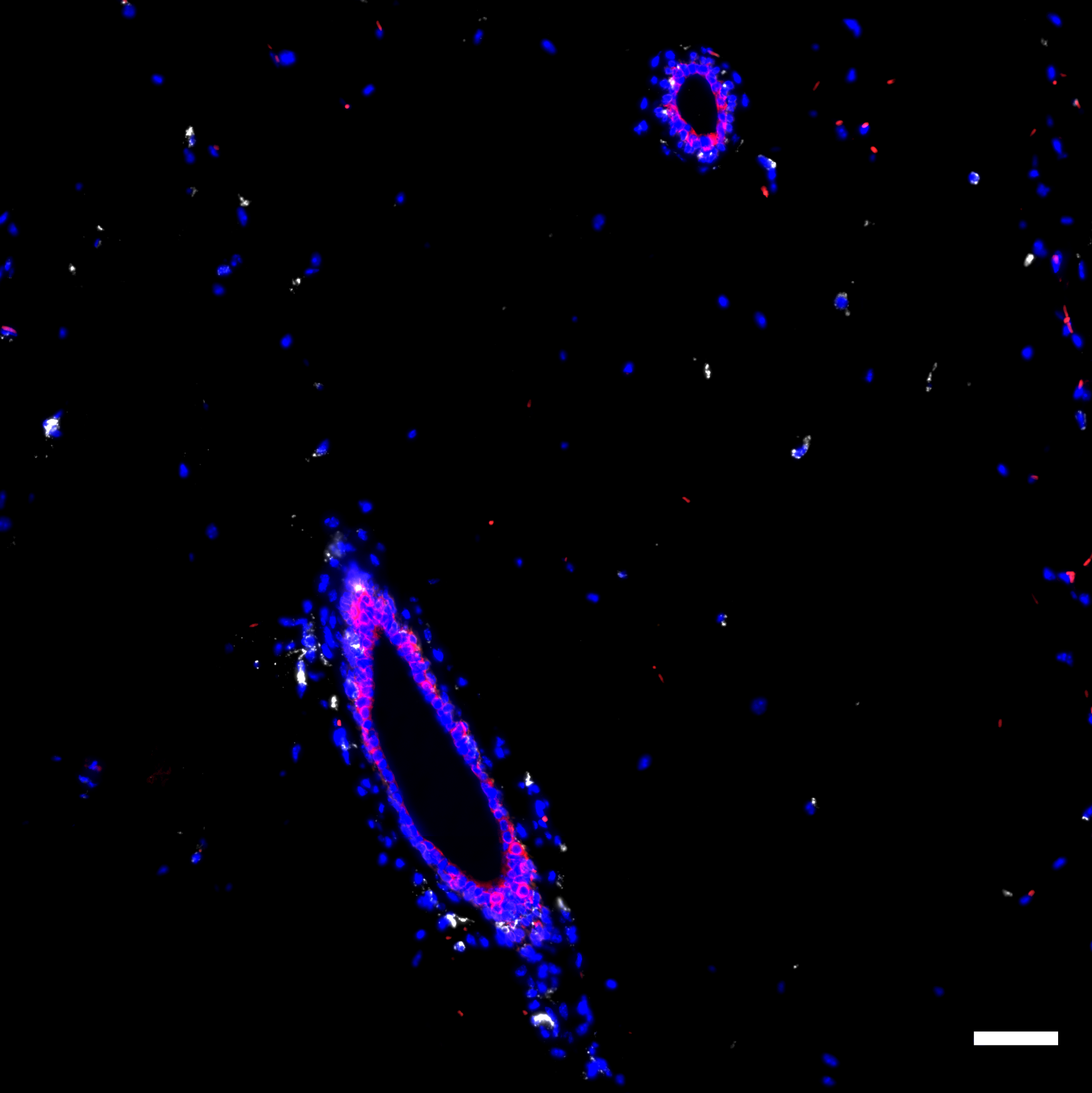

Supplement: Supplementary file 7 — Source data Fig. 5 [file 44319_2025_370_MOESM7_ESM.zip › Source Data Fig 5/5D/CTL K14 K8 CD68.tif]

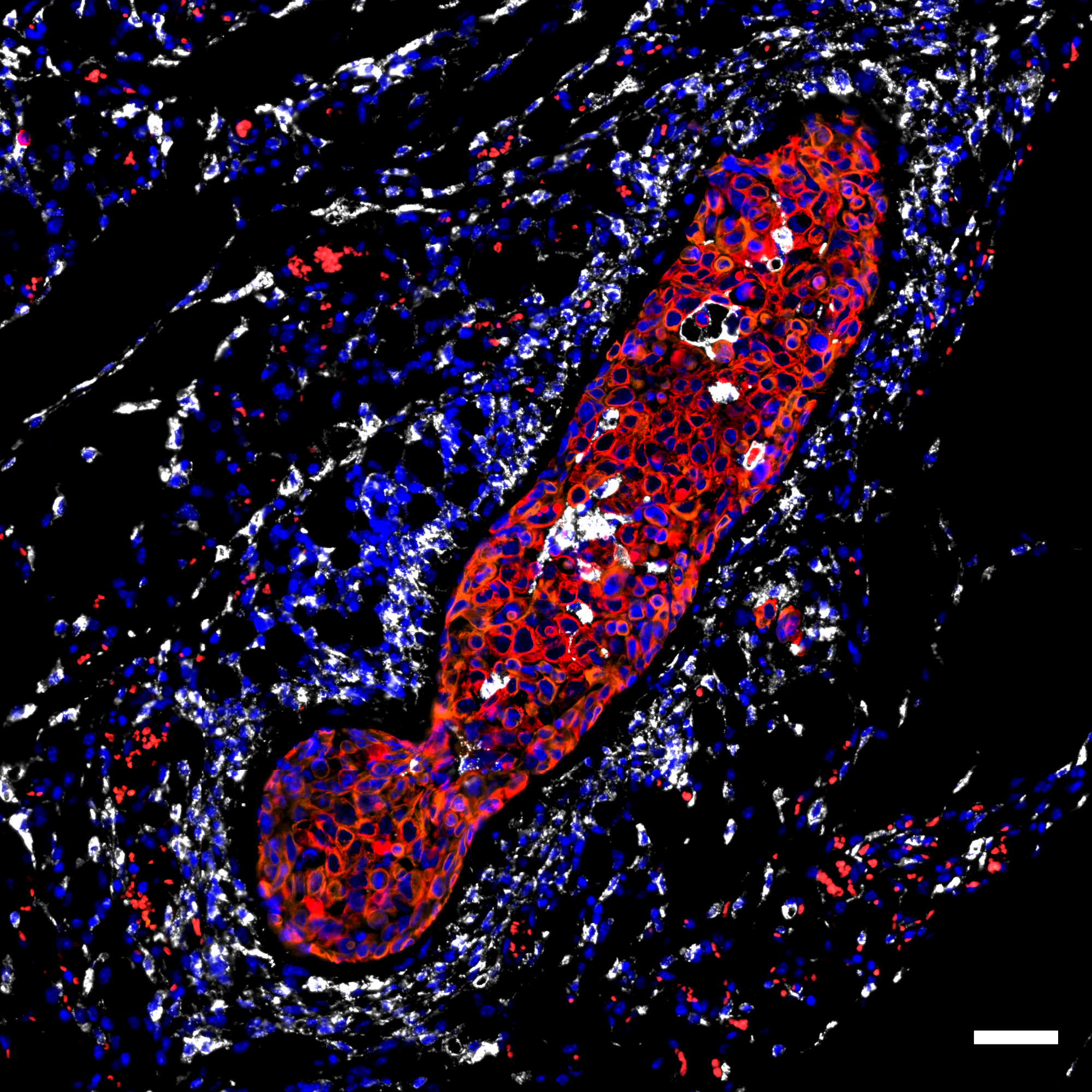

Supplement: Supplementary file 7 — Source data Fig. 5 [file 44319_2025_370_MOESM7_ESM.zip › Source Data Fig 5/5D/L12KO K14 K8 CD68.tif]

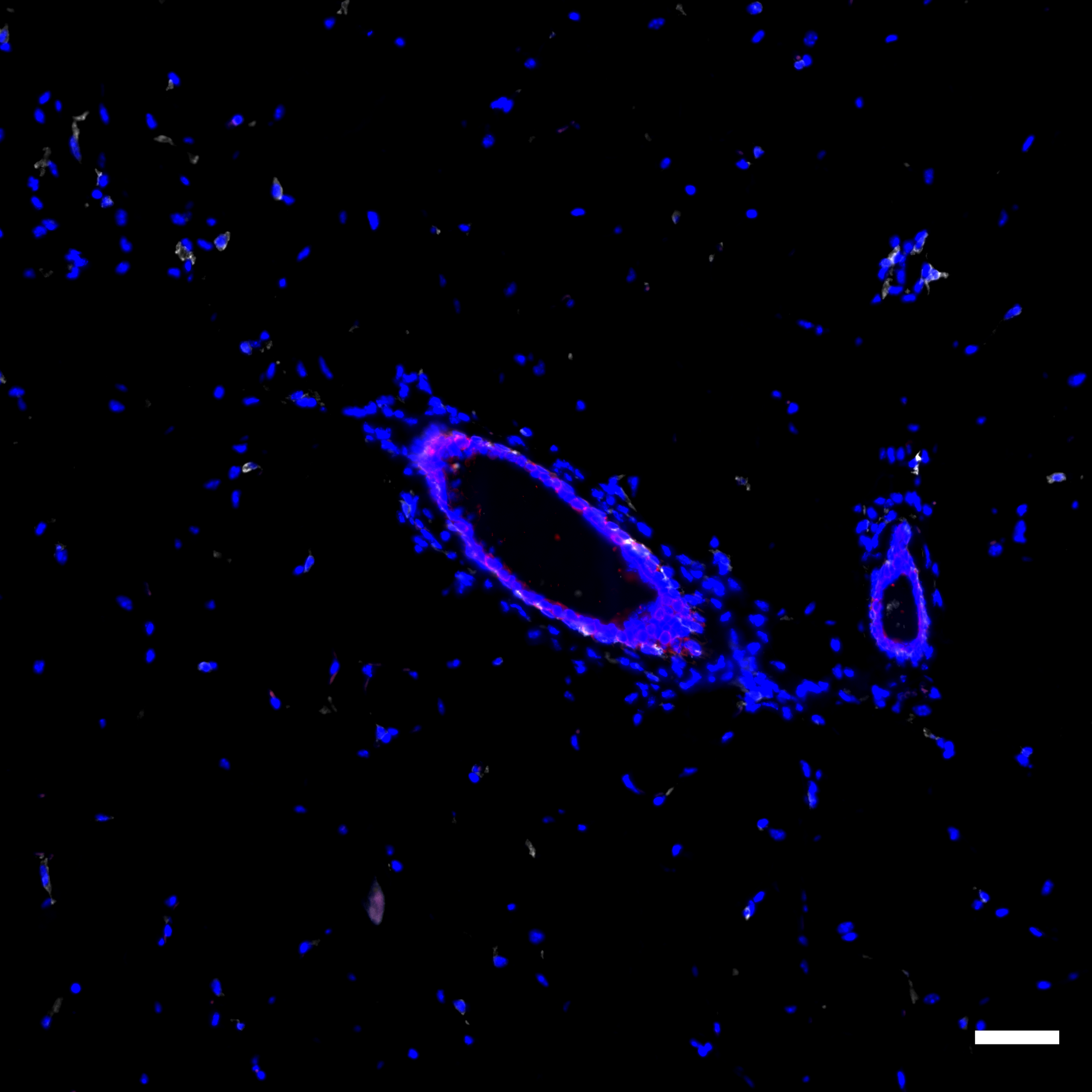

Supplement: Supplementary file 7 — Source data Fig. 5 [file 44319_2025_370_MOESM7_ESM.zip › Source Data Fig 5/5E/CTL K14 K8 F480.tif]

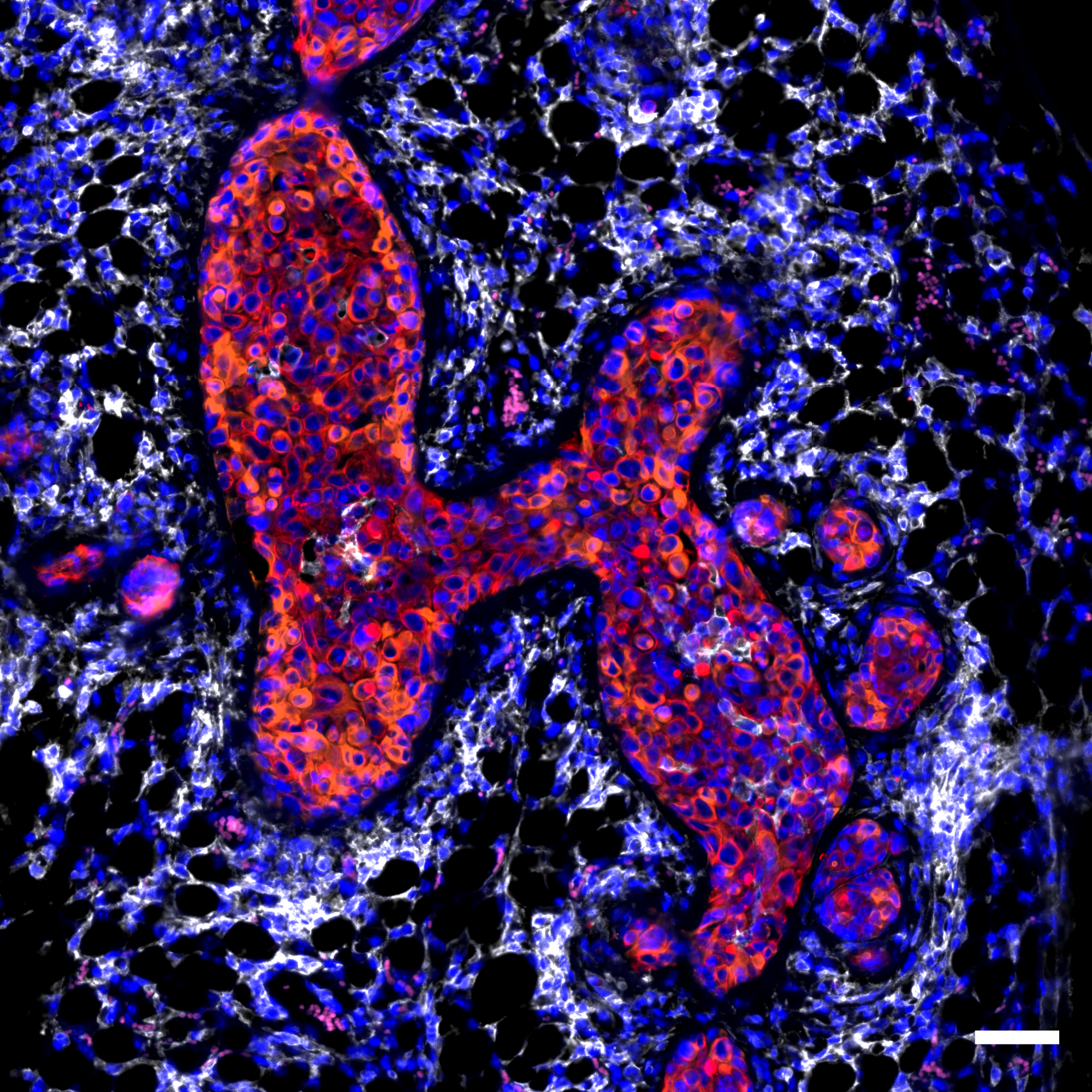

Supplement: Supplementary file 7 — Source data Fig. 5 [file 44319_2025_370_MOESM7_ESM.zip › Source Data Fig 5/5E/L12KO K14 K8 F480.tif]

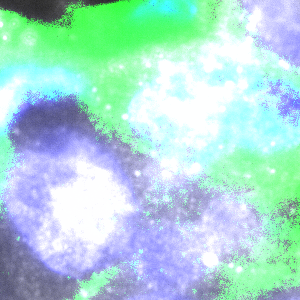

Supplement: Supplementary file 8 — Source data Fig. 6 [file 44319_2025_370_MOESM8_ESM.zip › Source Data Fig 6/6G/L12KO YFP TAZ Subset.tif]

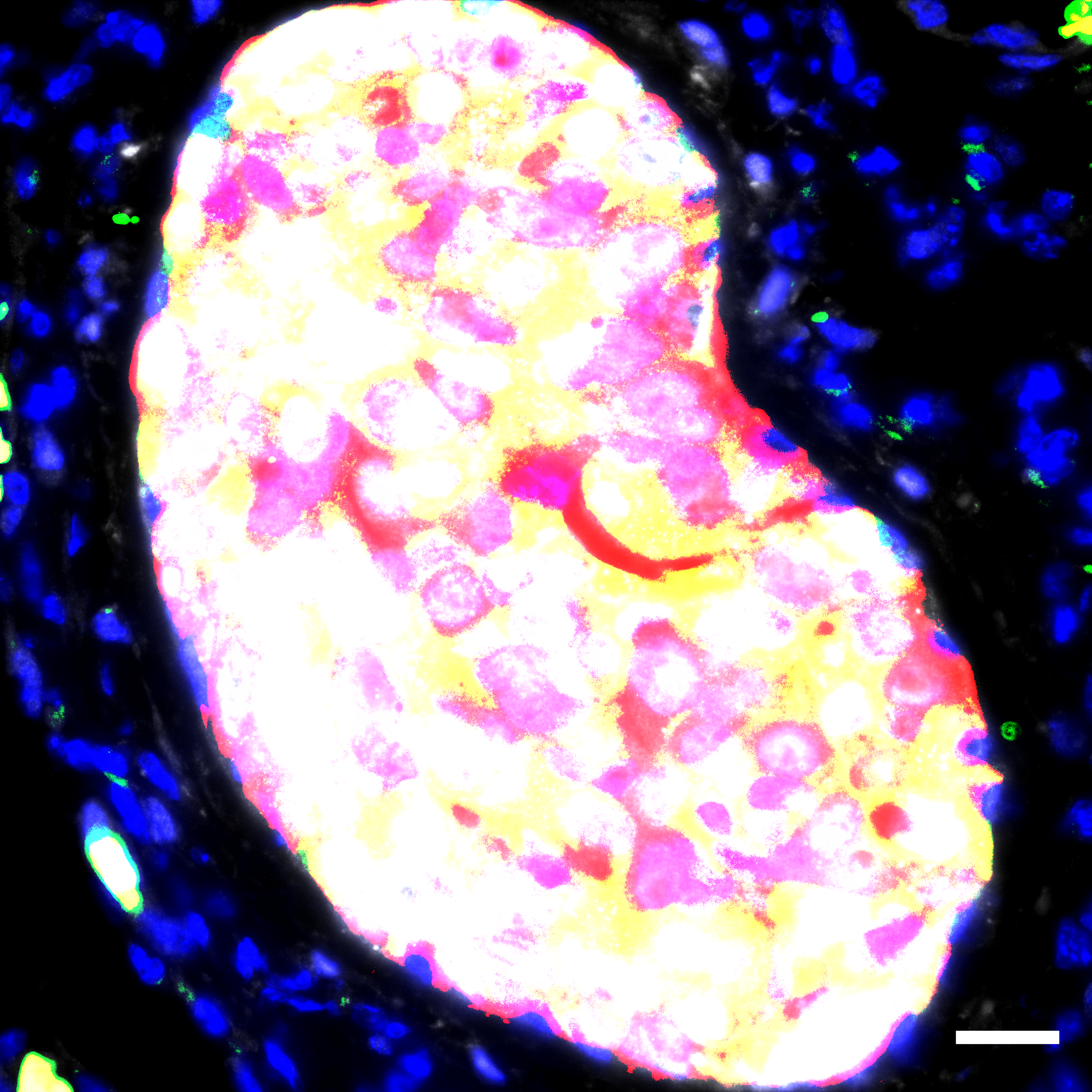

Supplement: Supplementary file 8 — Source data Fig. 6 [file 44319_2025_370_MOESM8_ESM.zip › Source Data Fig 6/6G/L12KO K8 YFP TAZ.tif]

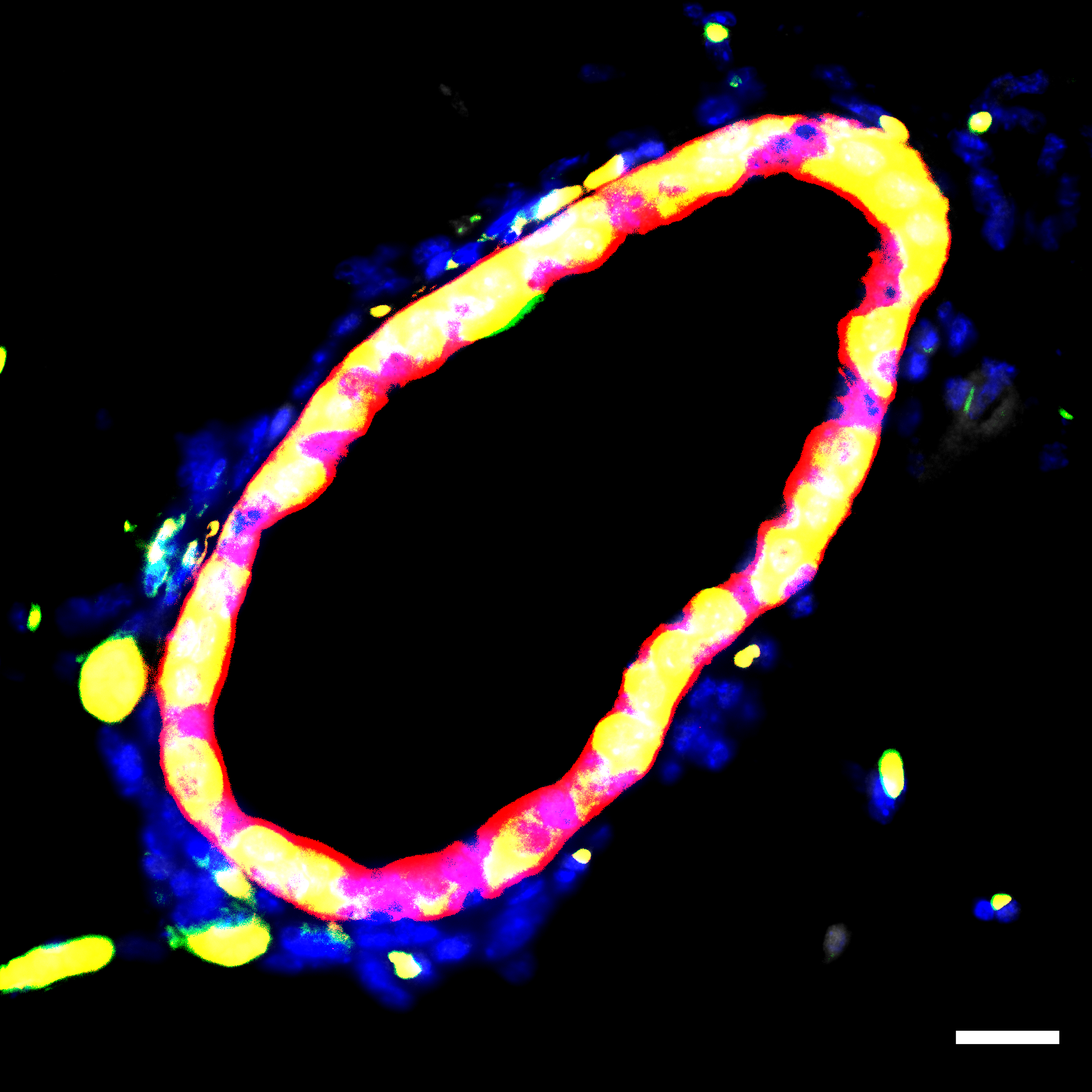

Supplement: Supplementary file 8 — Source data Fig. 6 [file 44319_2025_370_MOESM8_ESM.zip › Source Data Fig 6/6G/CTL K8 YFP TAZ.tif]

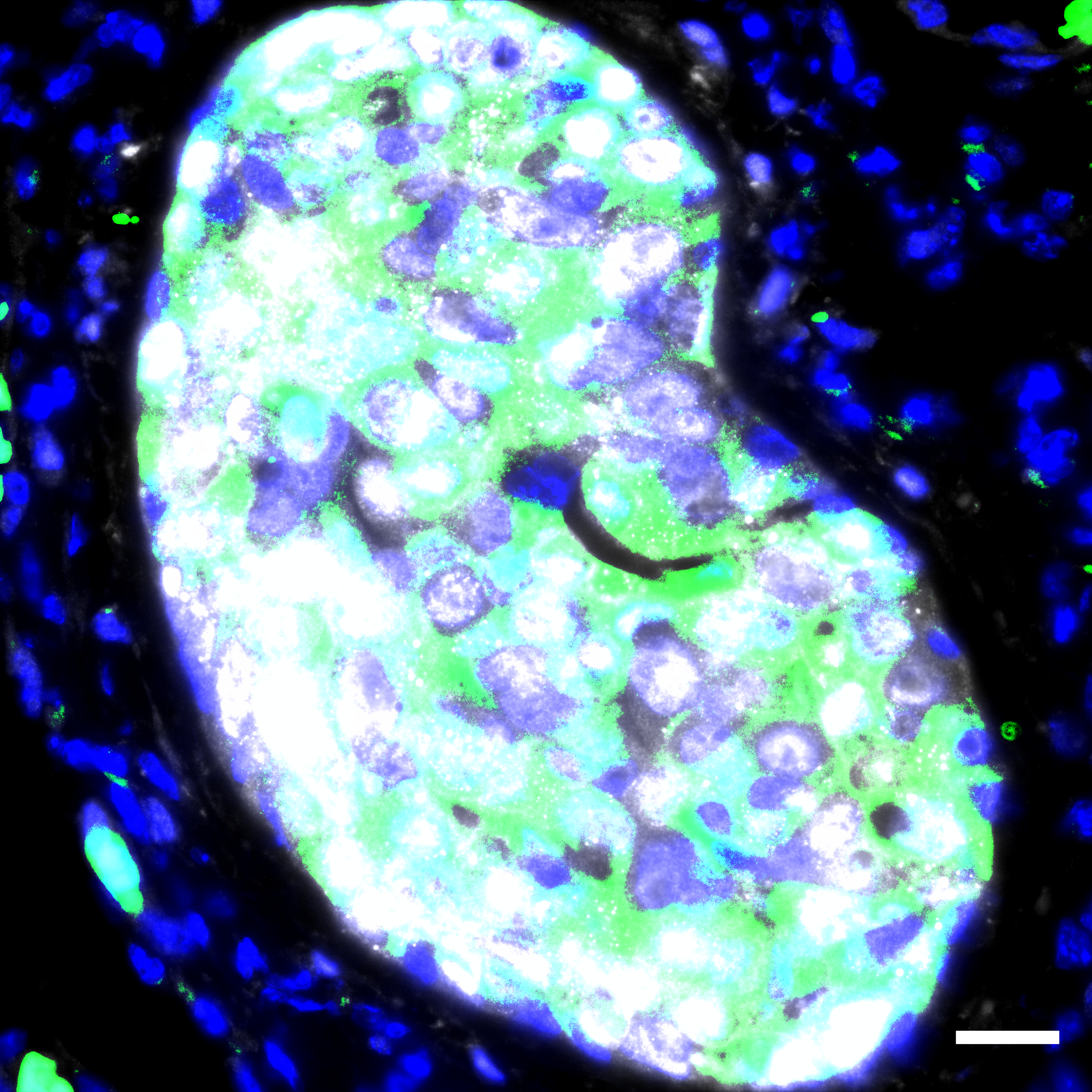

Supplement: Supplementary file 8 — Source data Fig. 6 [file 44319_2025_370_MOESM8_ESM.zip › Source Data Fig 6/6G/L12KO YFP TAZ.tif]

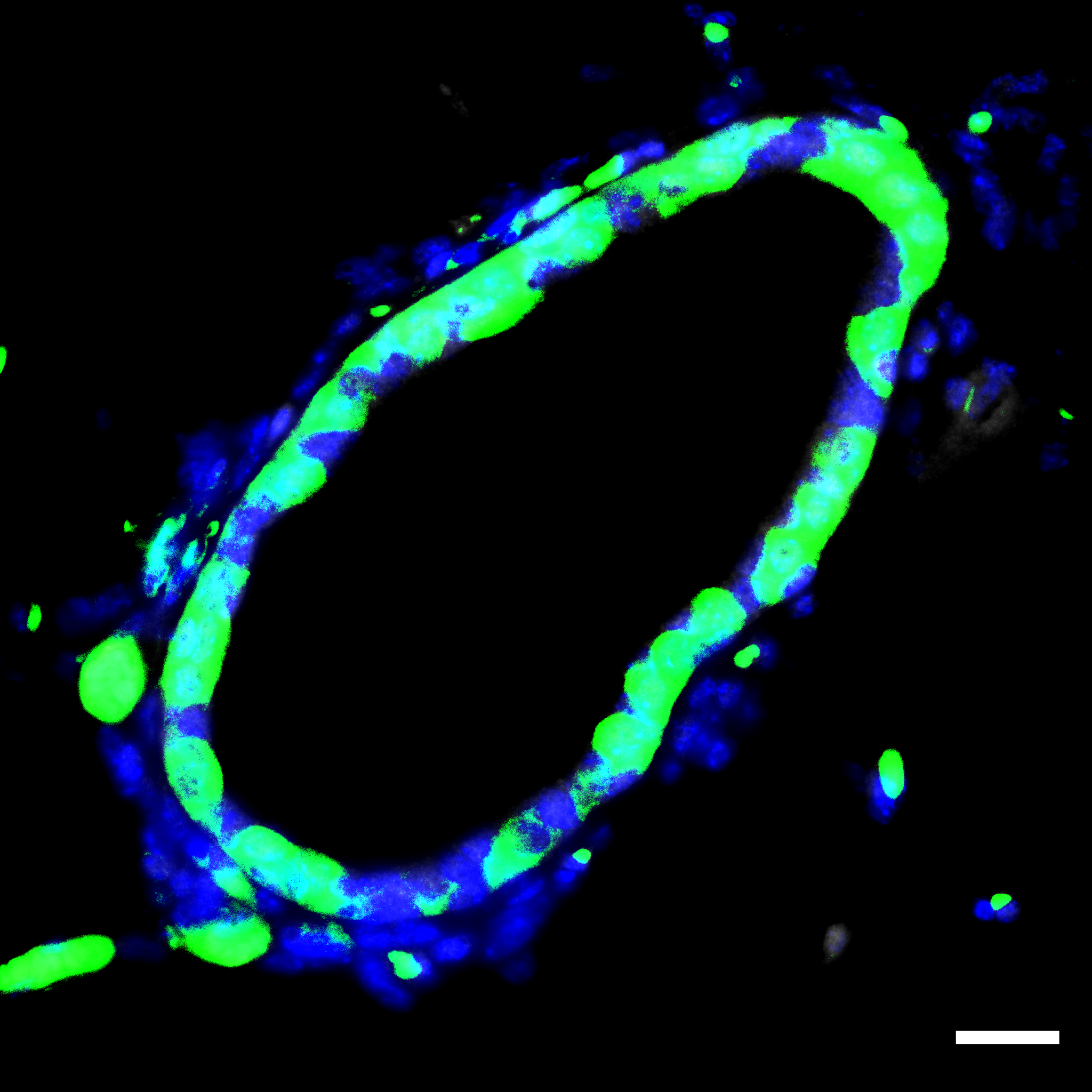

Supplement: Supplementary file 8 — Source data Fig. 6 [file 44319_2025_370_MOESM8_ESM.zip › Source Data Fig 6/6G/CTL YFP TAZ.tif]

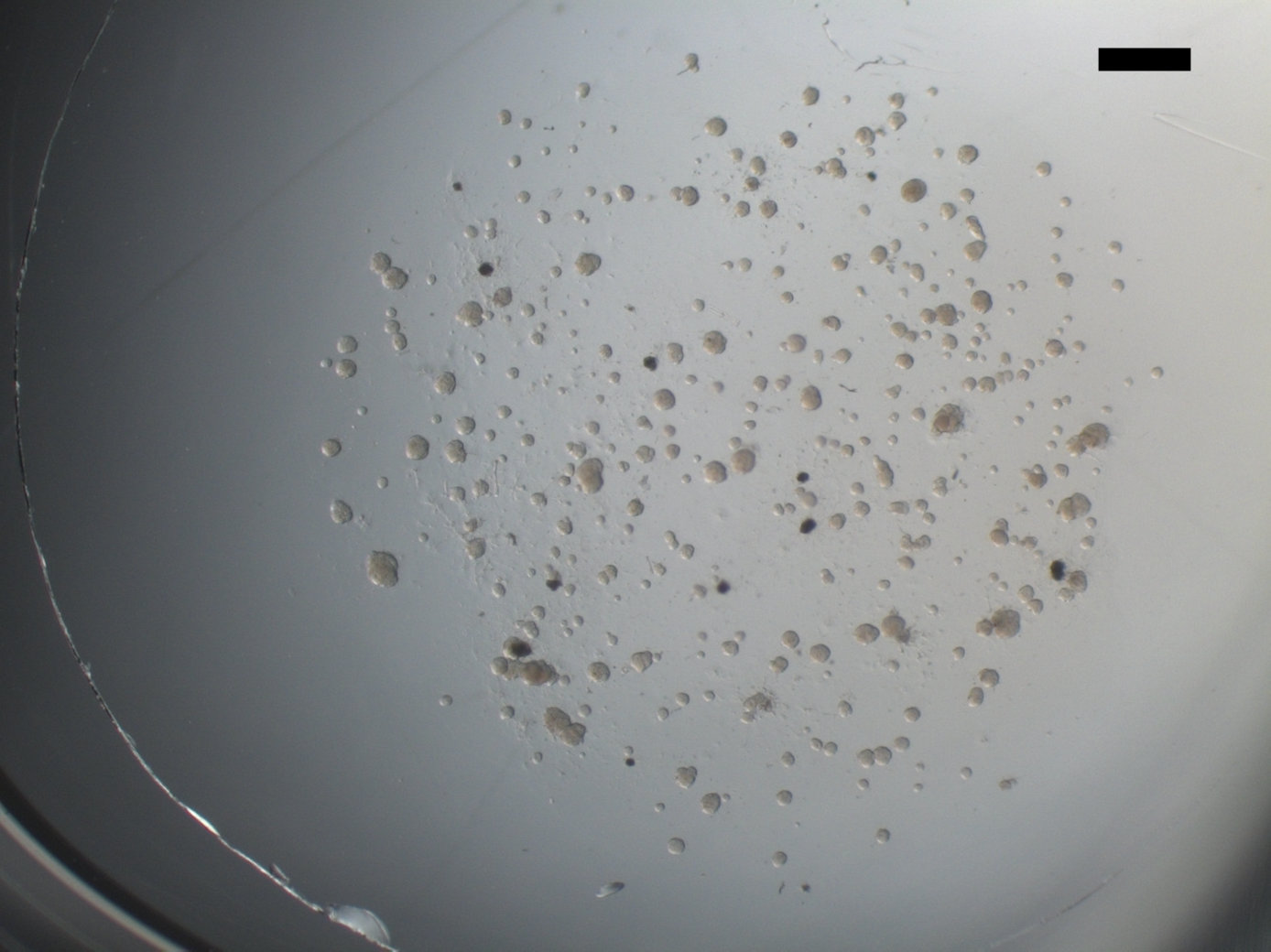

Supplement: Supplementary file 8 — Source data Fig. 6 [file 44319_2025_370_MOESM8_ESM.zip › Source Data Fig 6/6E/L12KO + PF573228.tif]

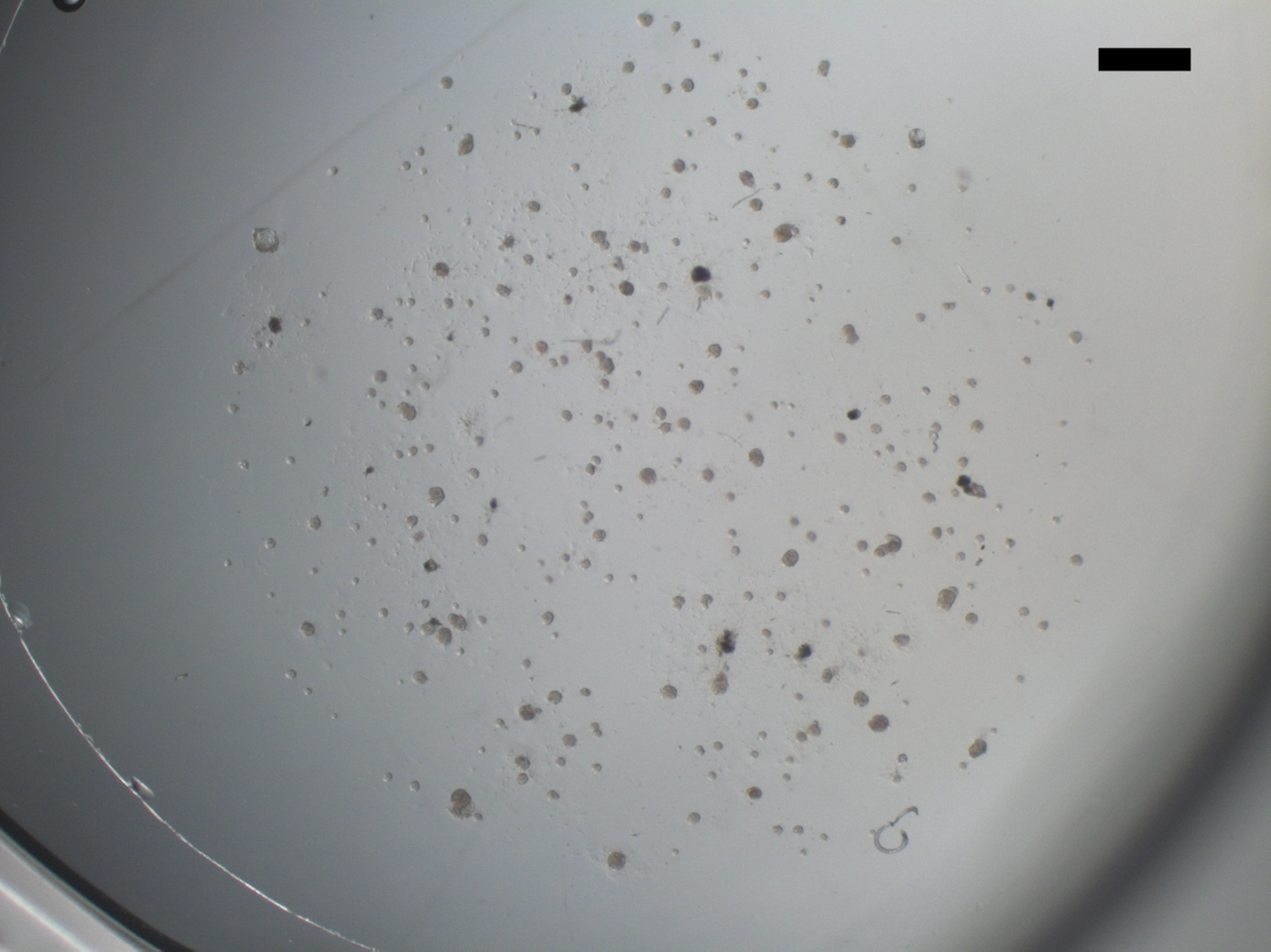

Supplement: Supplementary file 8 — Source data Fig. 6 [file 44319_2025_370_MOESM8_ESM.zip › Source Data Fig 6/6E/CTL + PF573228.tif]

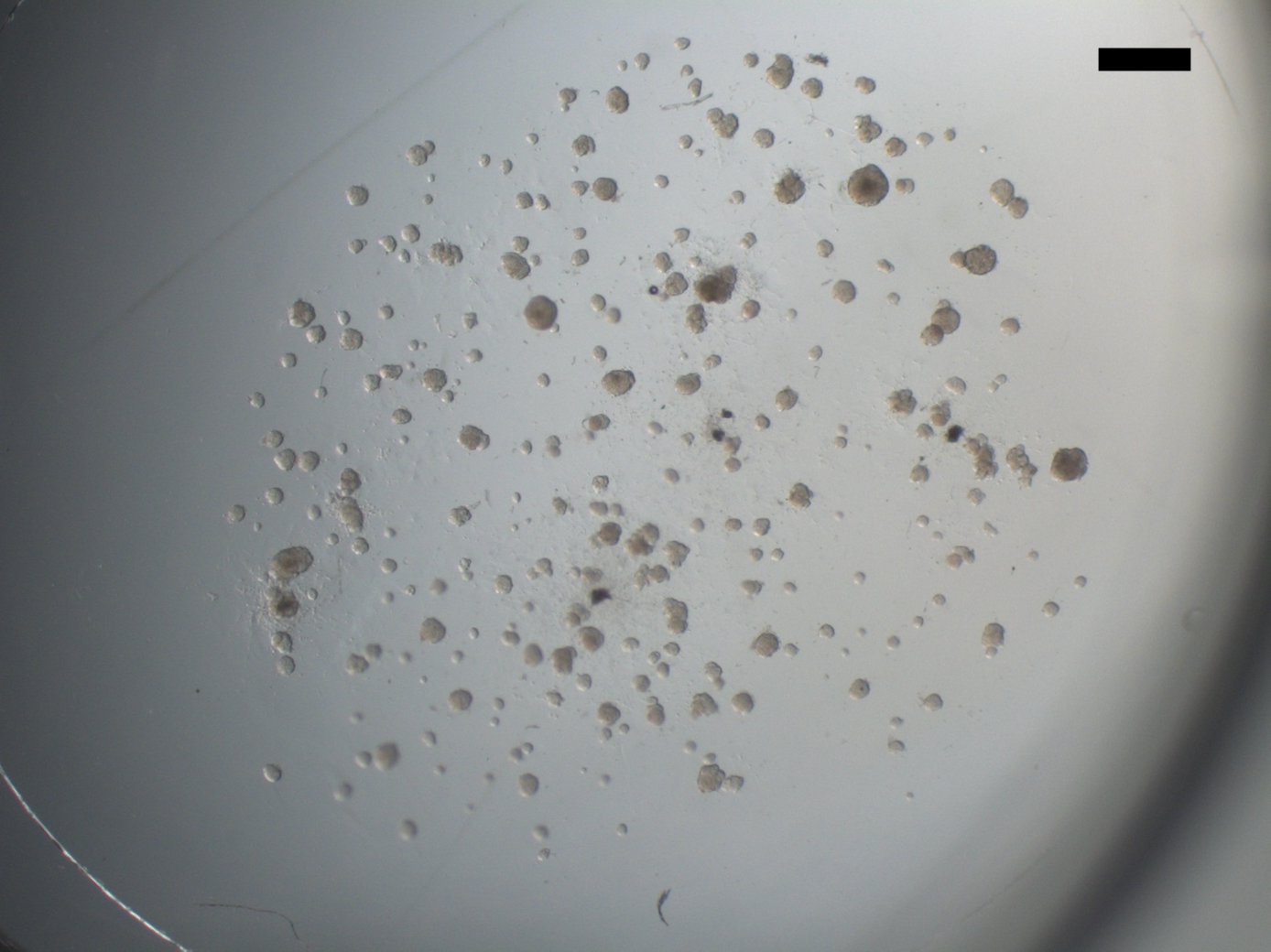

Supplement: Supplementary file 8 — Source data Fig. 6 [file 44319_2025_370_MOESM8_ESM.zip › Source Data Fig 6/6E/L12KO + Vehicle.tif]

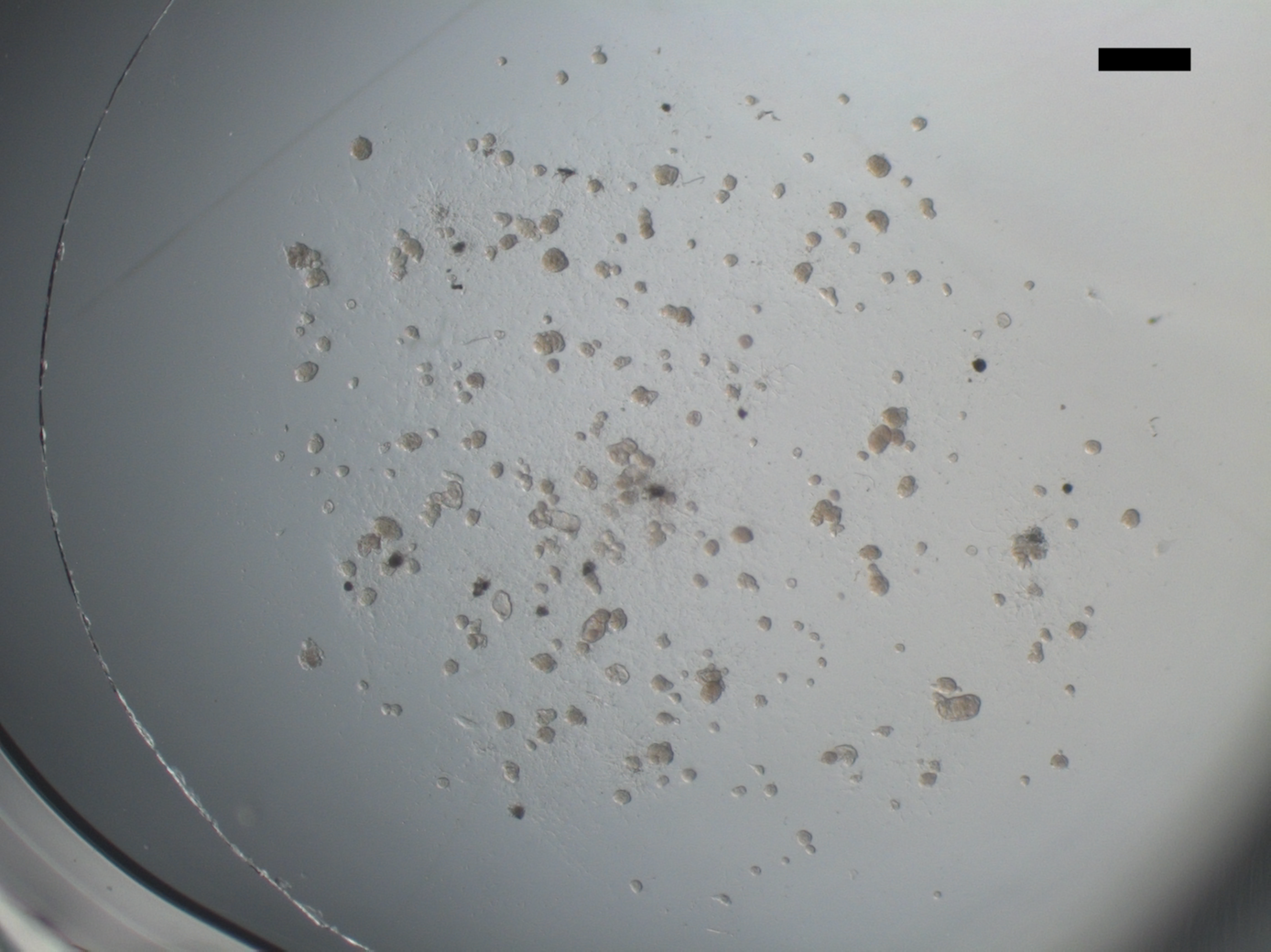

Supplement: Supplementary file 8 — Source data Fig. 6 [file 44319_2025_370_MOESM8_ESM.zip › Source Data Fig 6/6E/CTL + Vehicle.tif]

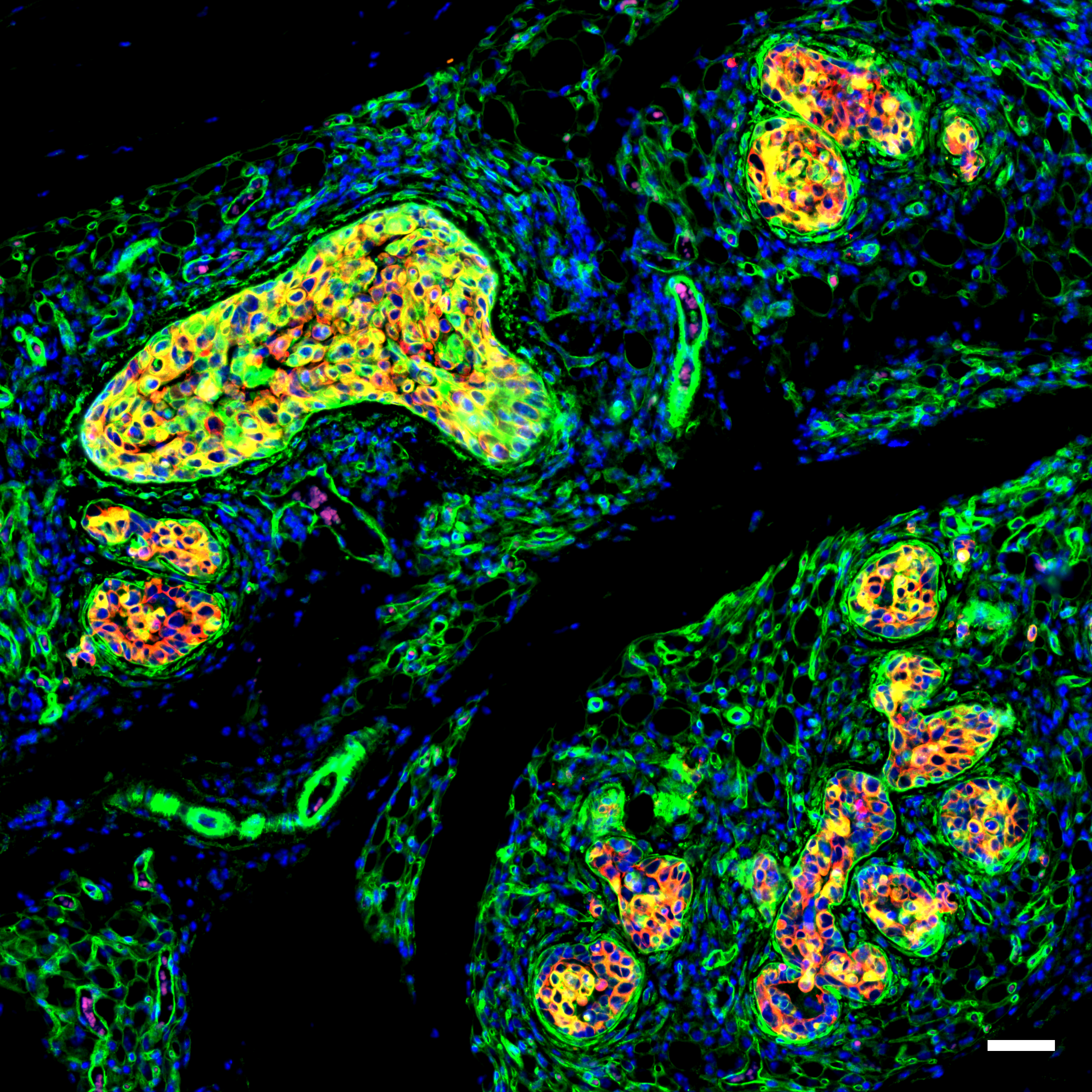

Supplement: Supplementary file 8 — Source data Fig. 6 [file 44319_2025_370_MOESM8_ESM.zip › Source Data Fig 6/6D/L12KO K14 K8 ITGB1.tif]

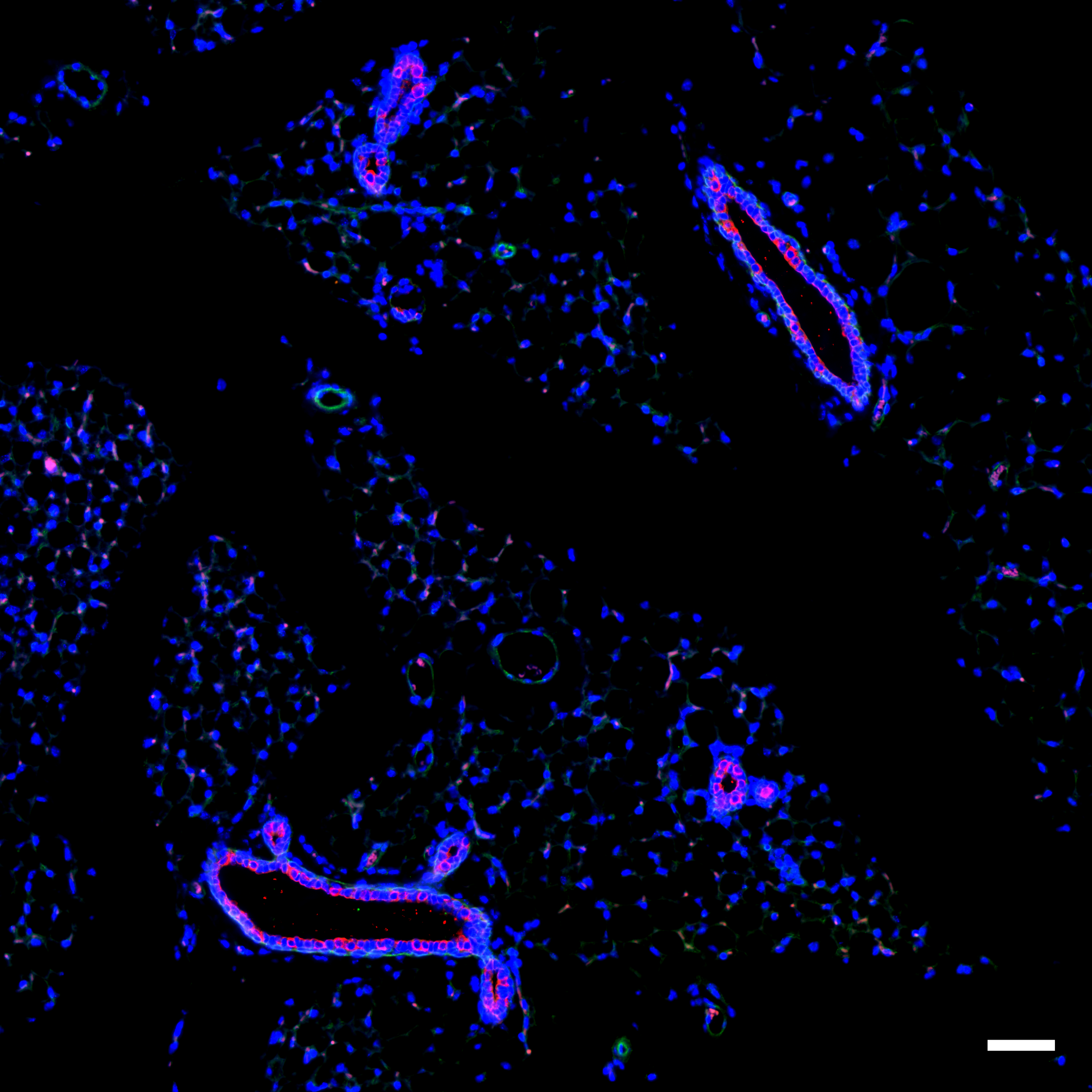

Supplement: Supplementary file 8 — Source data Fig. 6 [file 44319_2025_370_MOESM8_ESM.zip › Source Data Fig 6/6D/CTL K14 K8 ITGB1.tif]

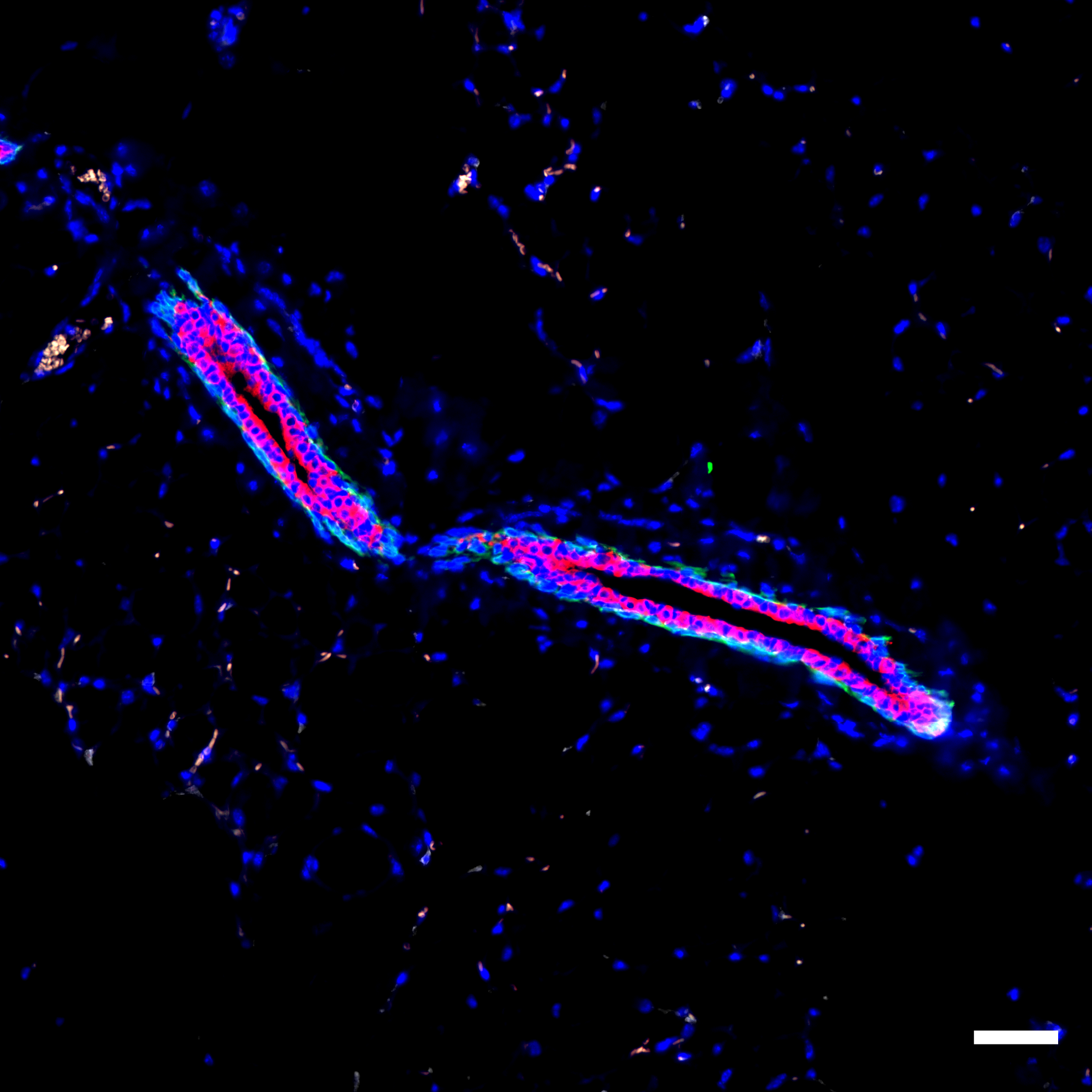

Supplement: Supplementary file 9 — Source data Fig. 7 [file 44319_2025_370_MOESM9_ESM.zip › Source Data Fig 7/7D/CTL (LATS12ff;lslEYFP;NoCre) + Vehicle K14 K8 F480.tif]

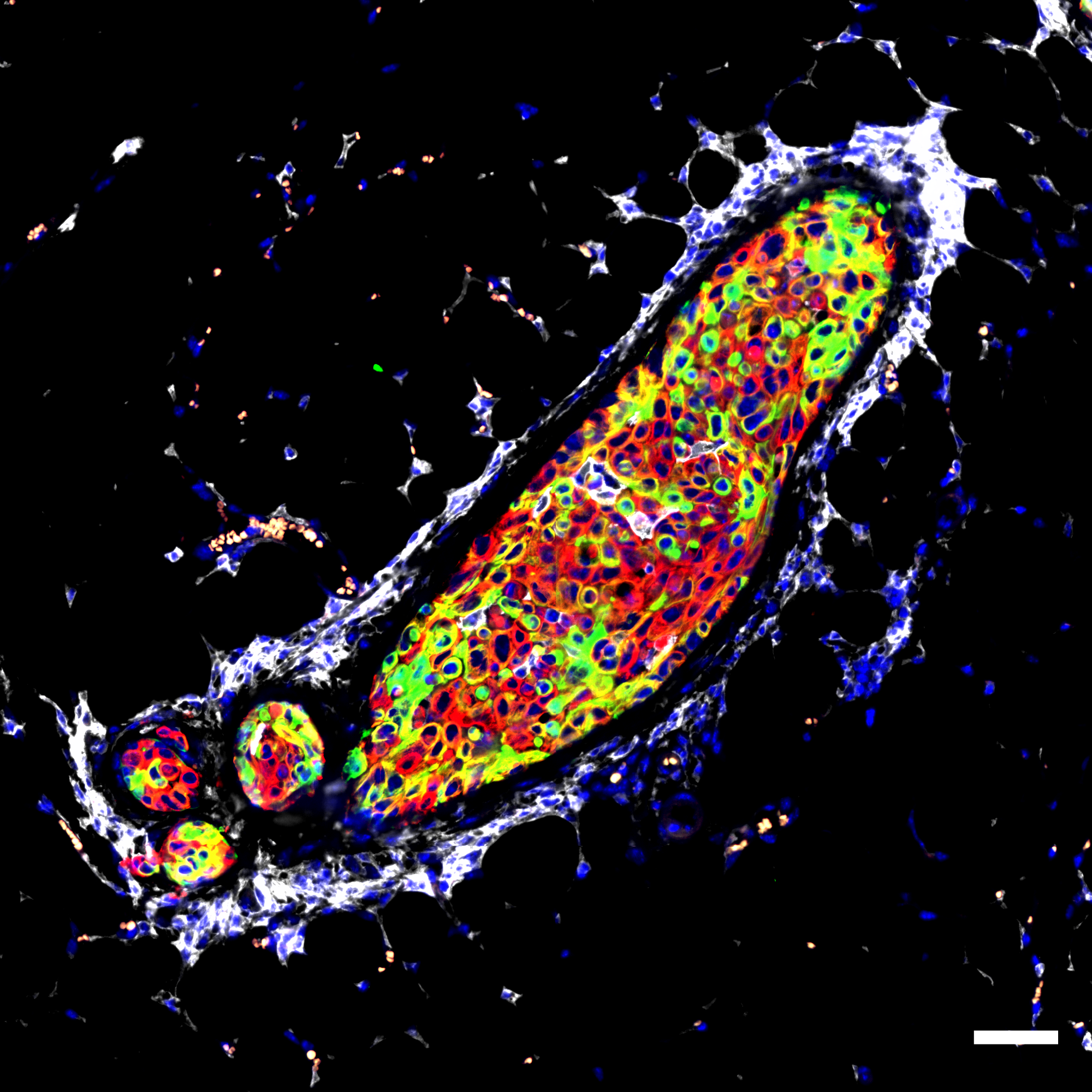

Supplement: Supplementary file 9 — Source data Fig. 7 [file 44319_2025_370_MOESM9_ESM.zip › Source Data Fig 7/7D/L12KO (LATS12ff;lslEYFP;K8Cre) + Vehicle K14 K8 F480.tif]

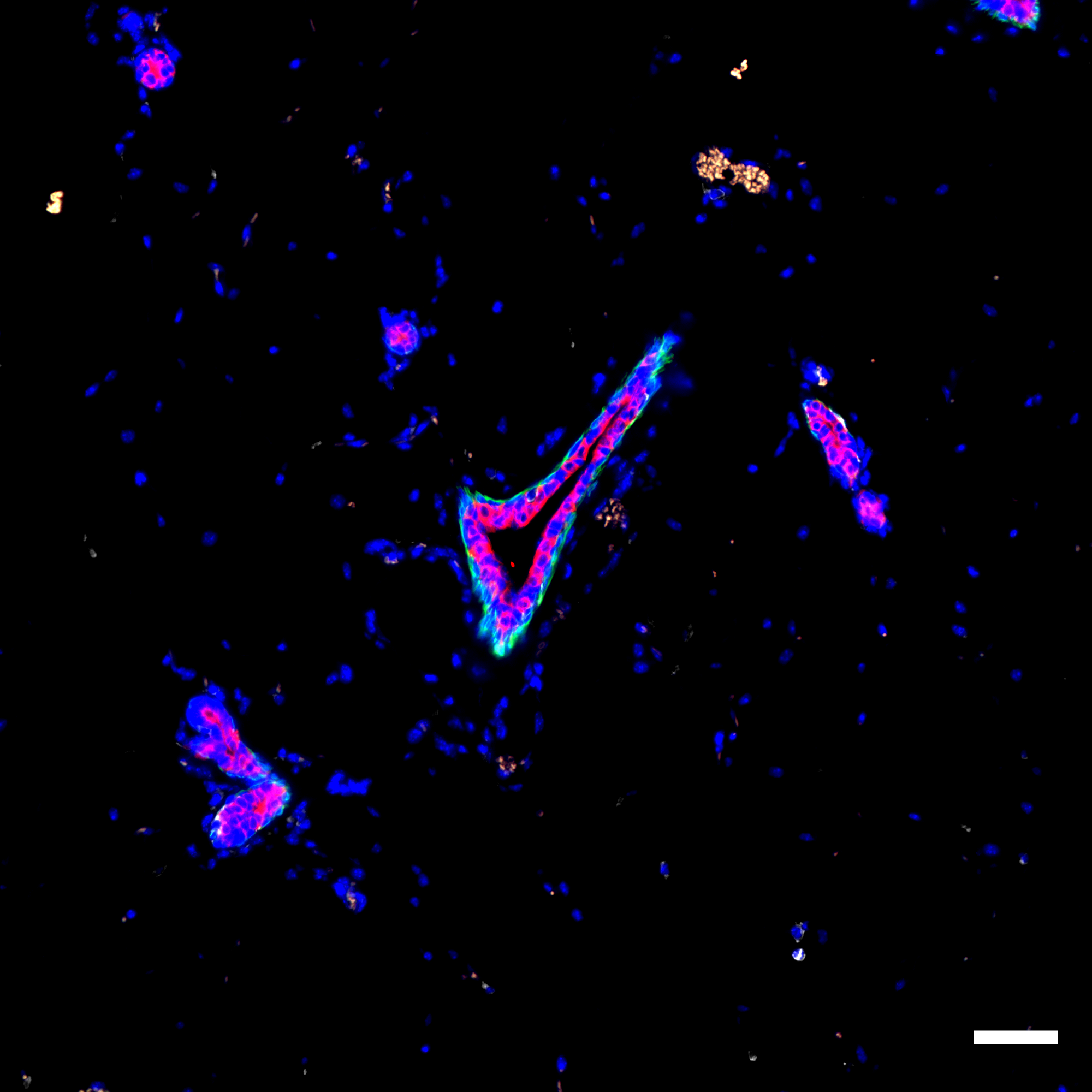

Supplement: Supplementary file 9 — Source data Fig. 7 [file 44319_2025_370_MOESM9_ESM.zip › Source Data Fig 7/7D/CTL (LATS12ff;lslEYFP;NoCre) + VT104 K14 K8 F480.tif]

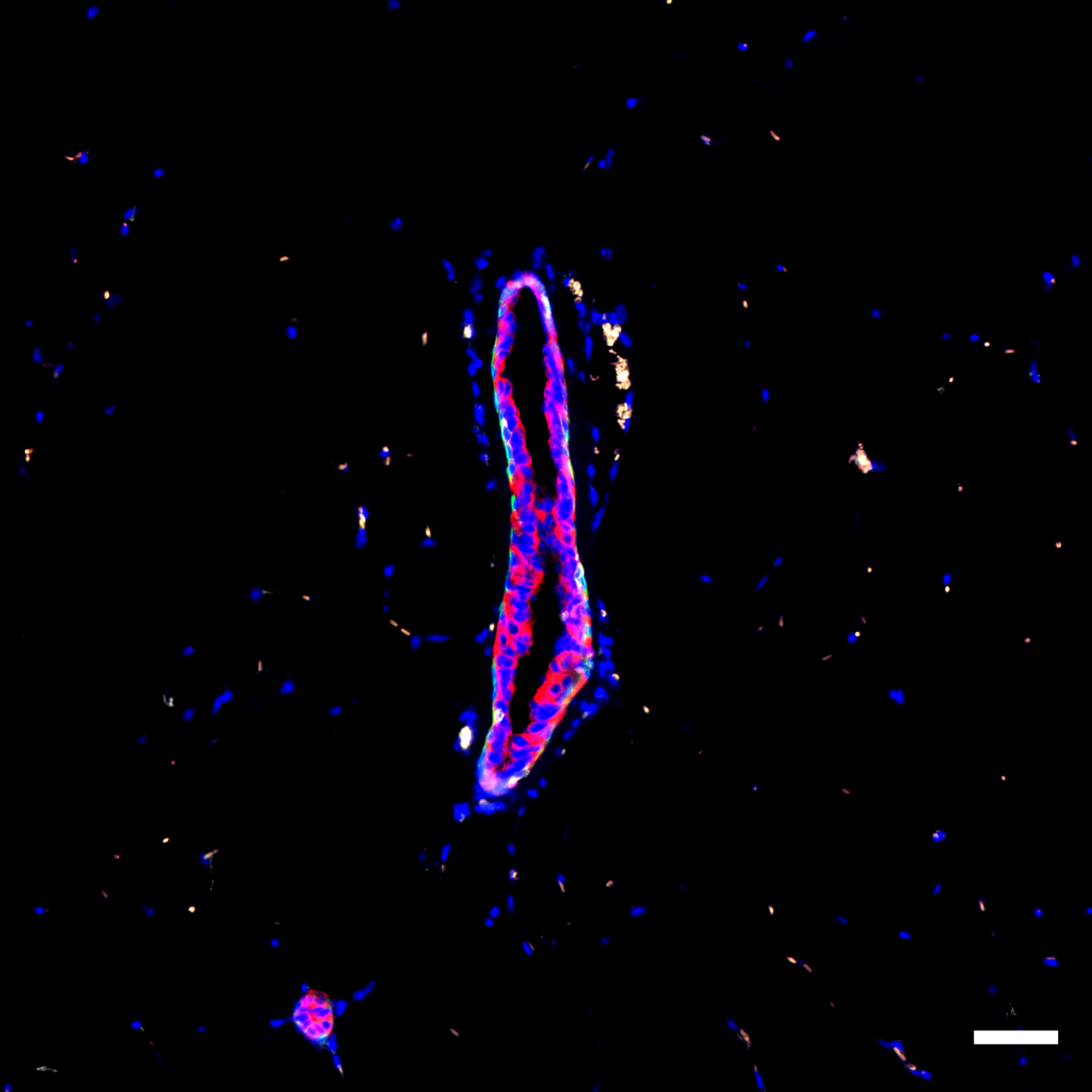

Supplement: Supplementary file 9 — Source data Fig. 7 [file 44319_2025_370_MOESM9_ESM.zip › Source Data Fig 7/7D/L12KO (LATS12ff;lslEYFP;K8Cre) + VT104 K14 K8 F480.tif]

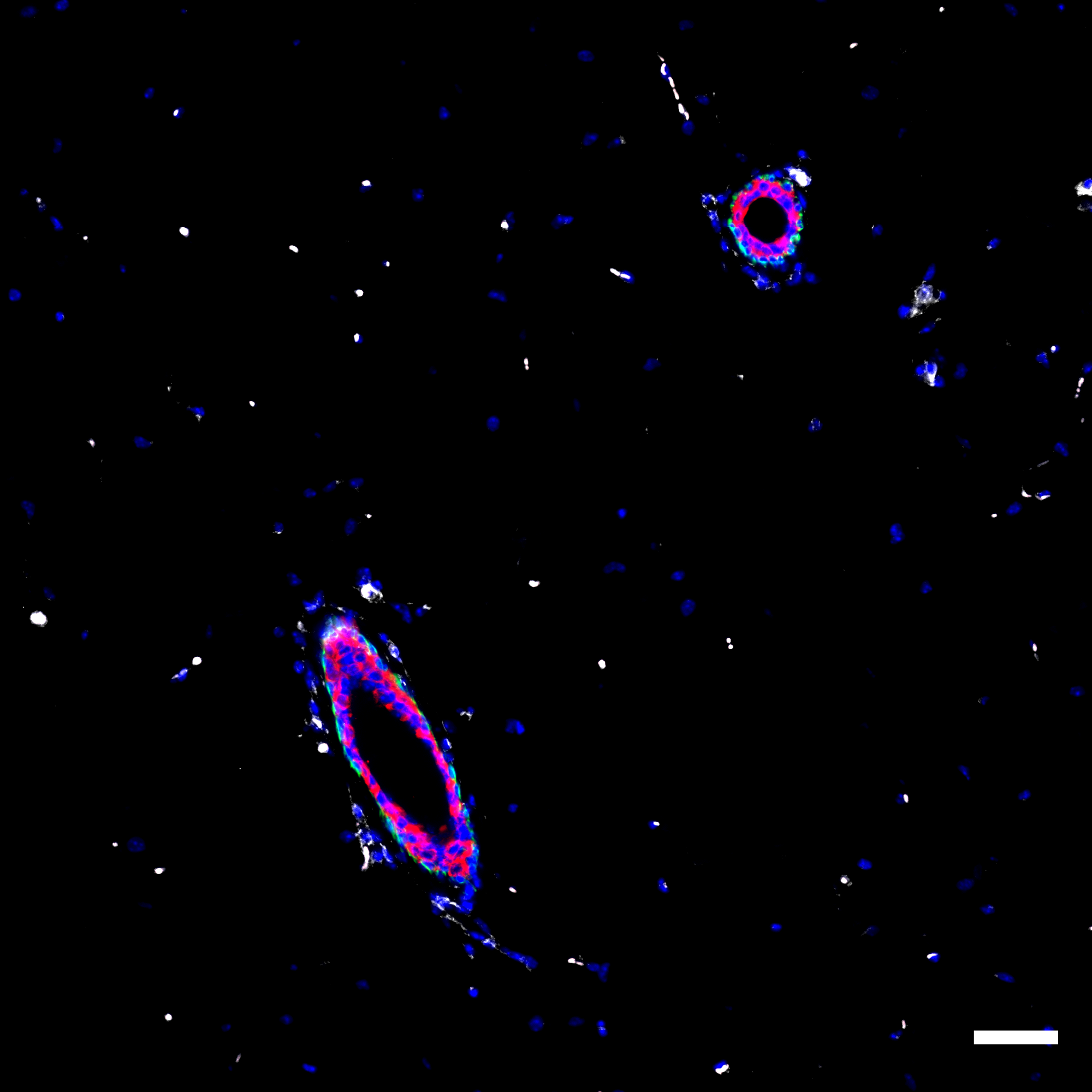

Supplement: Supplementary file 9 — Source data Fig. 7 [file 44319_2025_370_MOESM9_ESM.zip › Source Data Fig 7/7C/L12KO (LATS12ff;lslEYFP;K8Cre) + VT104 K14 K8 PDGFRB.tif]

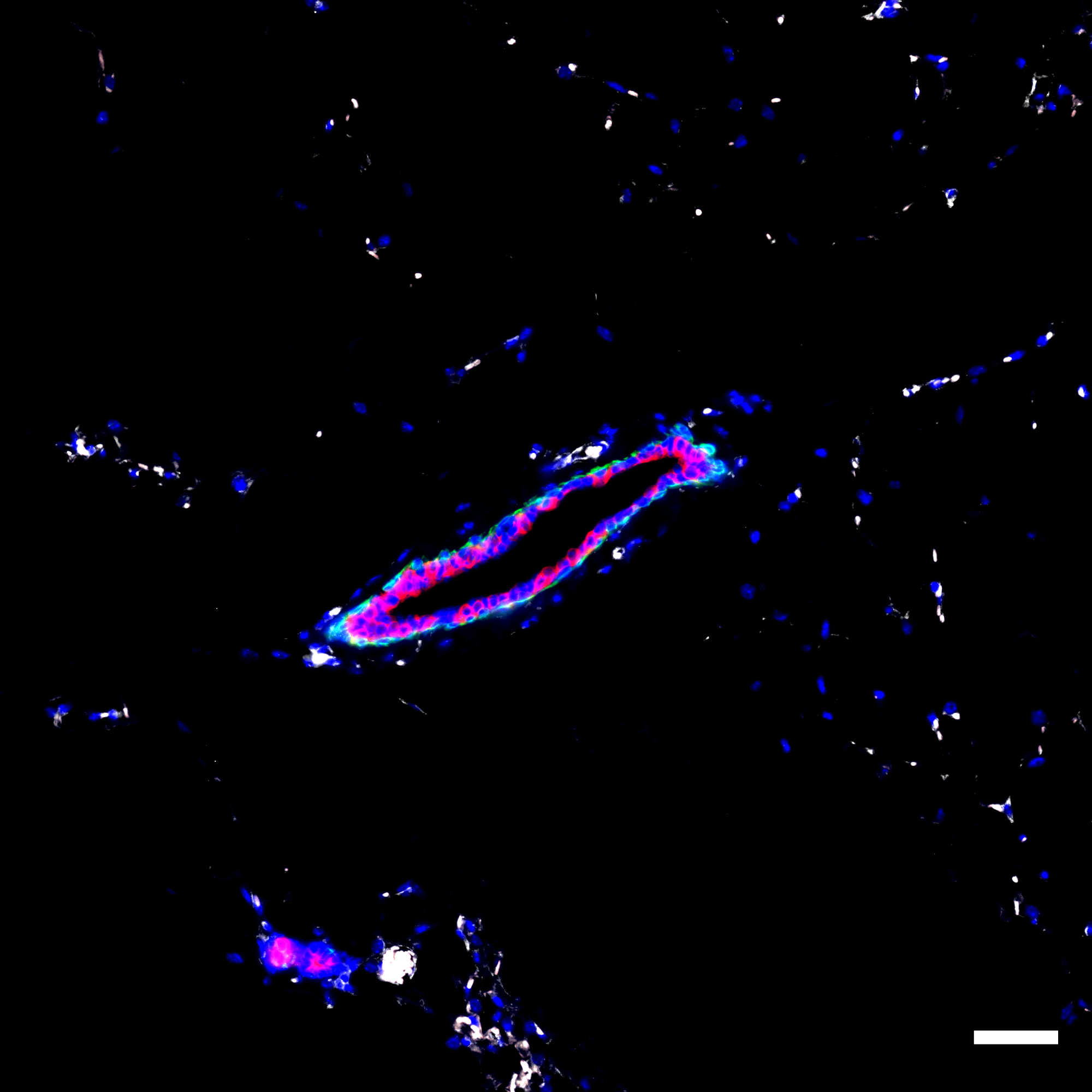

Supplement: Supplementary file 9 — Source data Fig. 7 [file 44319_2025_370_MOESM9_ESM.zip › Source Data Fig 7/7C/CTL (LATS12ff;lslEYFP;NoCre) + VT104 K14 K8 PDGFRB.tif]

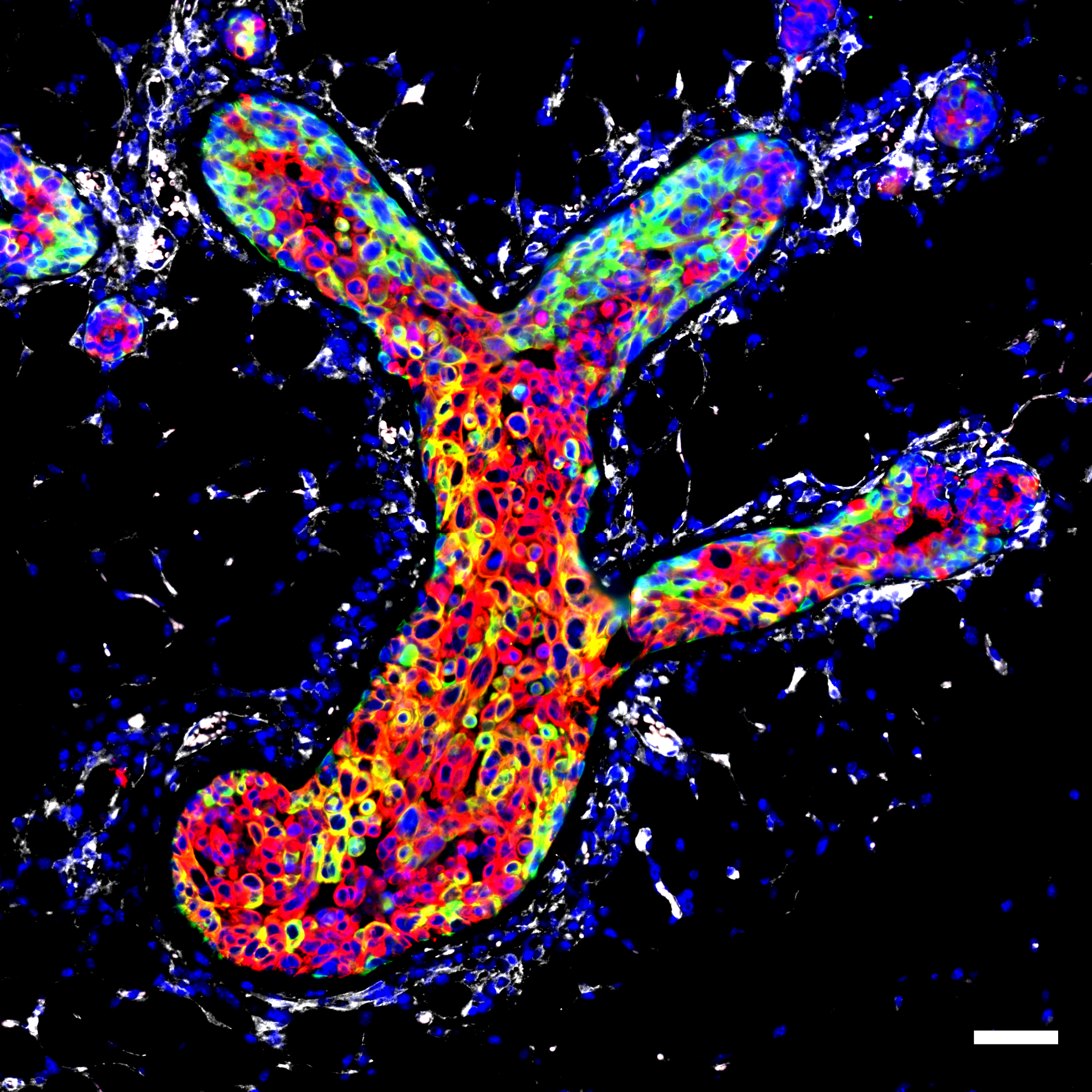

Supplement: Supplementary file 9 — Source data Fig. 7 [file 44319_2025_370_MOESM9_ESM.zip › Source Data Fig 7/7C/L12KO (LATS12ff;lslEYFP;K8Cre) + Vehicle K14 K8 PDGFRB.tif]

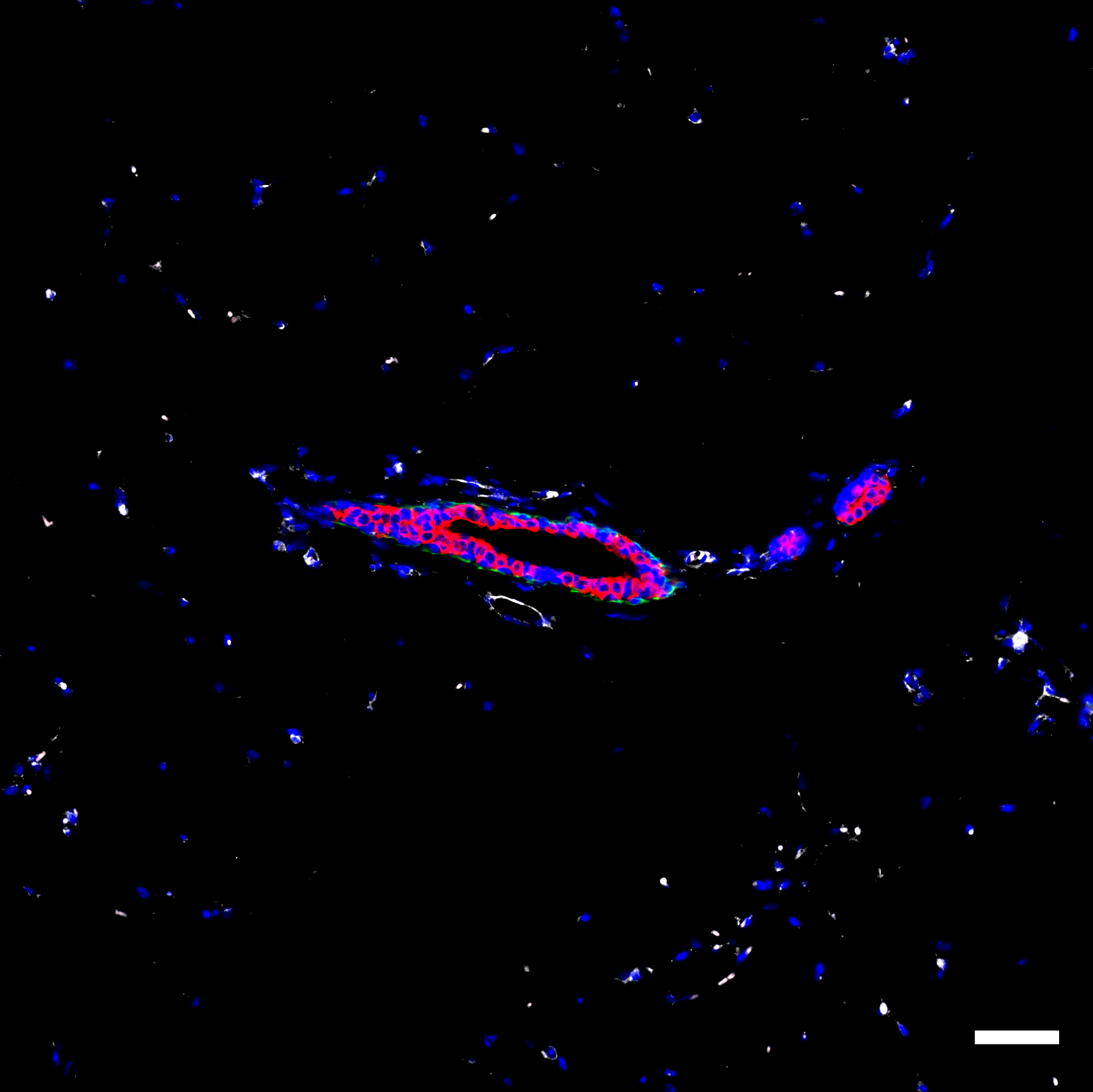

Supplement: Supplementary file 9 — Source data Fig. 7 [file 44319_2025_370_MOESM9_ESM.zip › Source Data Fig 7/7C/CTL (LATS12ff;lslEYFP;NoCre) + Vehicle K14 K8 PDGFRB.tif]

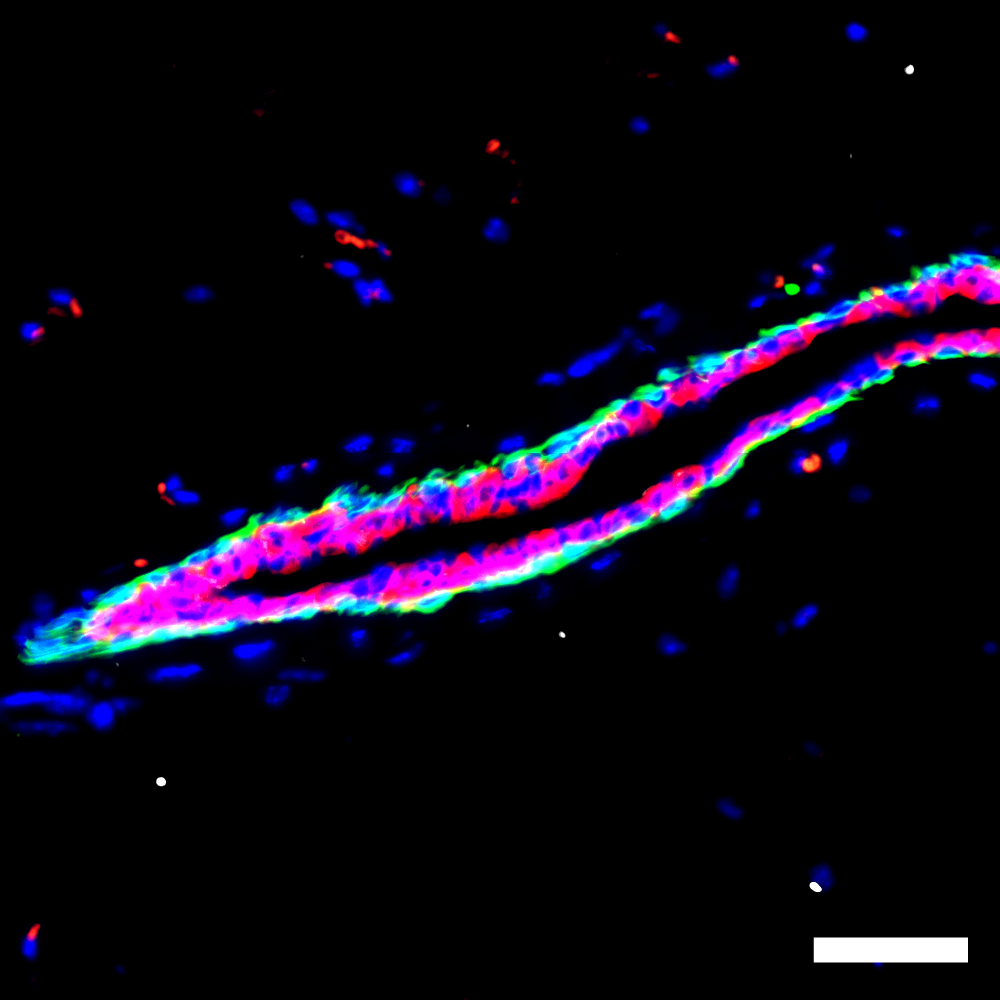

Supplement: Supplementary file 9 — Source data Fig. 7 [file 44319_2025_370_MOESM9_ESM.zip › Source Data Fig 7/7B/CTL (LATS12ff;lslEYFP;NoCre) + VT104 K14 K8 YFP.tif]

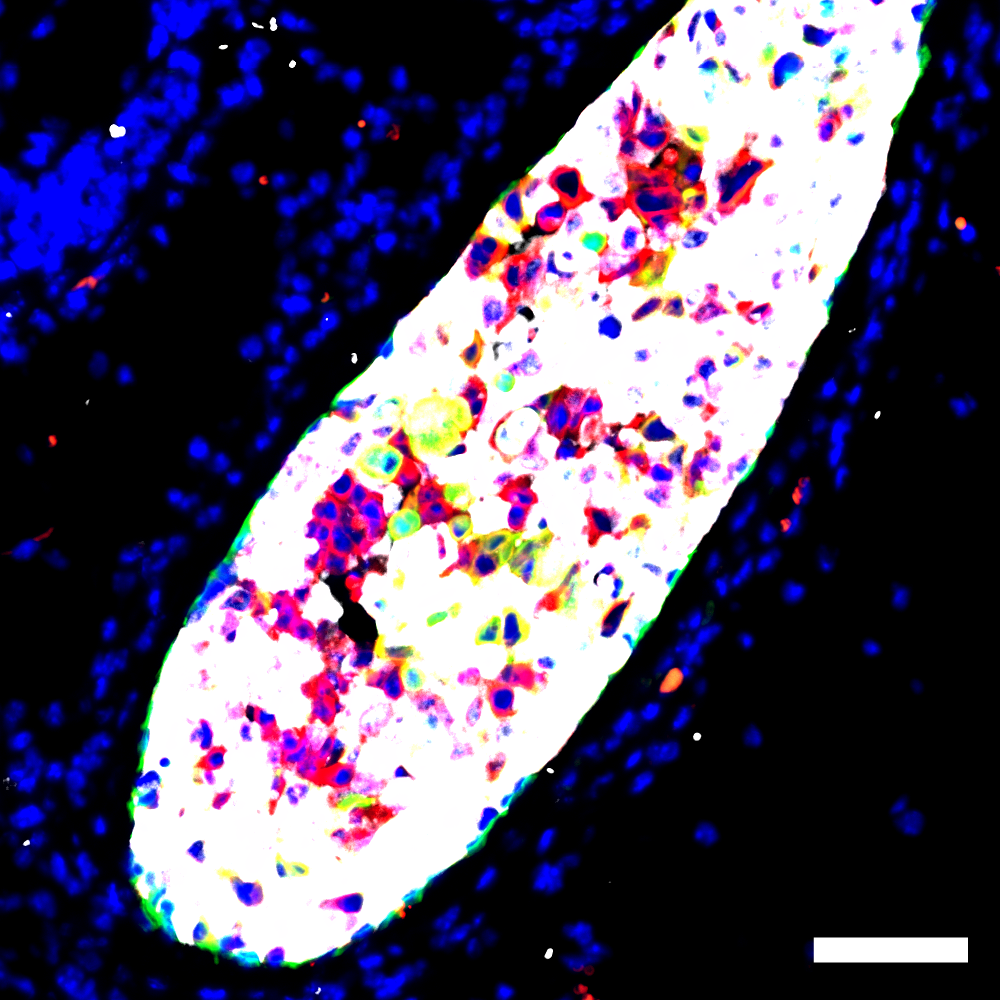

Supplement: Supplementary file 9 — Source data Fig. 7 [file 44319_2025_370_MOESM9_ESM.zip › Source Data Fig 7/7B/L12KO (LATS12ff;lslEYFP;K8Cre) + Vehicle K14 K8 YFP.tif]

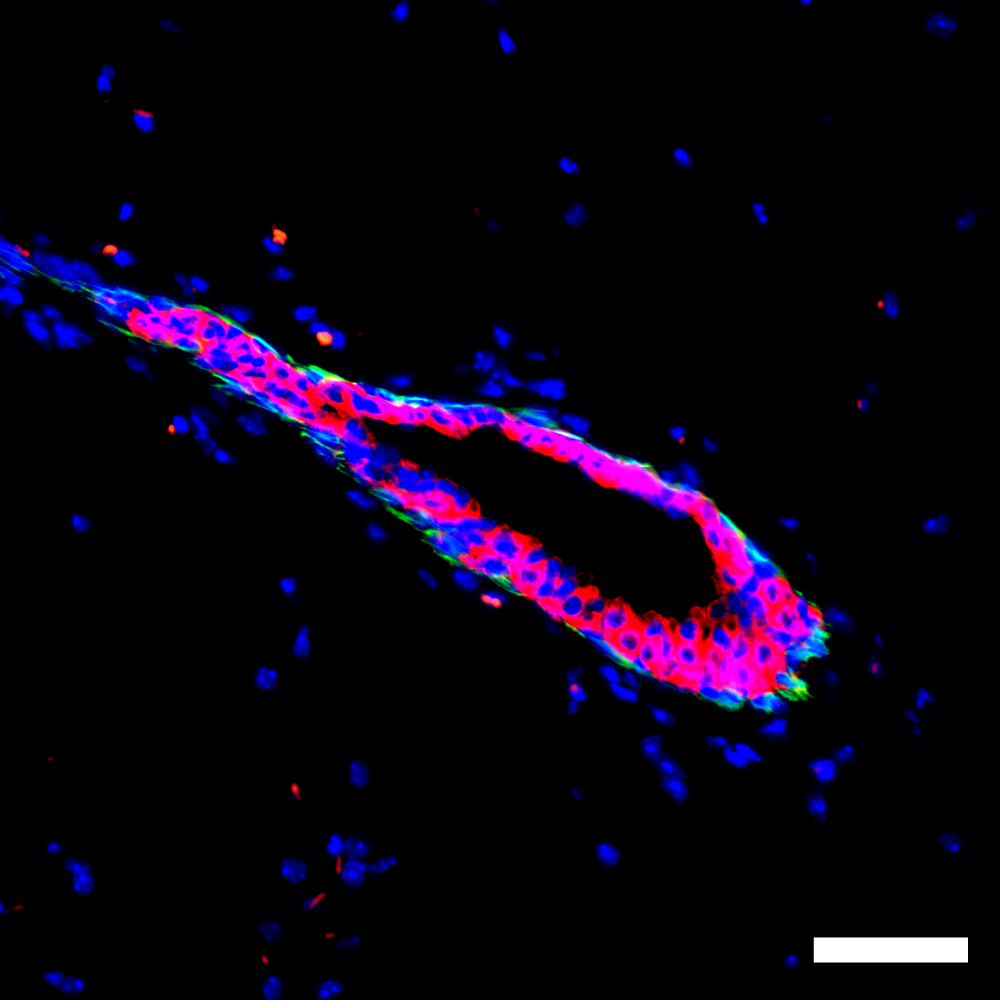

Supplement: Supplementary file 9 — Source data Fig. 7 [file 44319_2025_370_MOESM9_ESM.zip › Source Data Fig 7/7B/CTL (LATS12ff;lslEYFP;NoCre) + Vehicle K14 K8.tif]

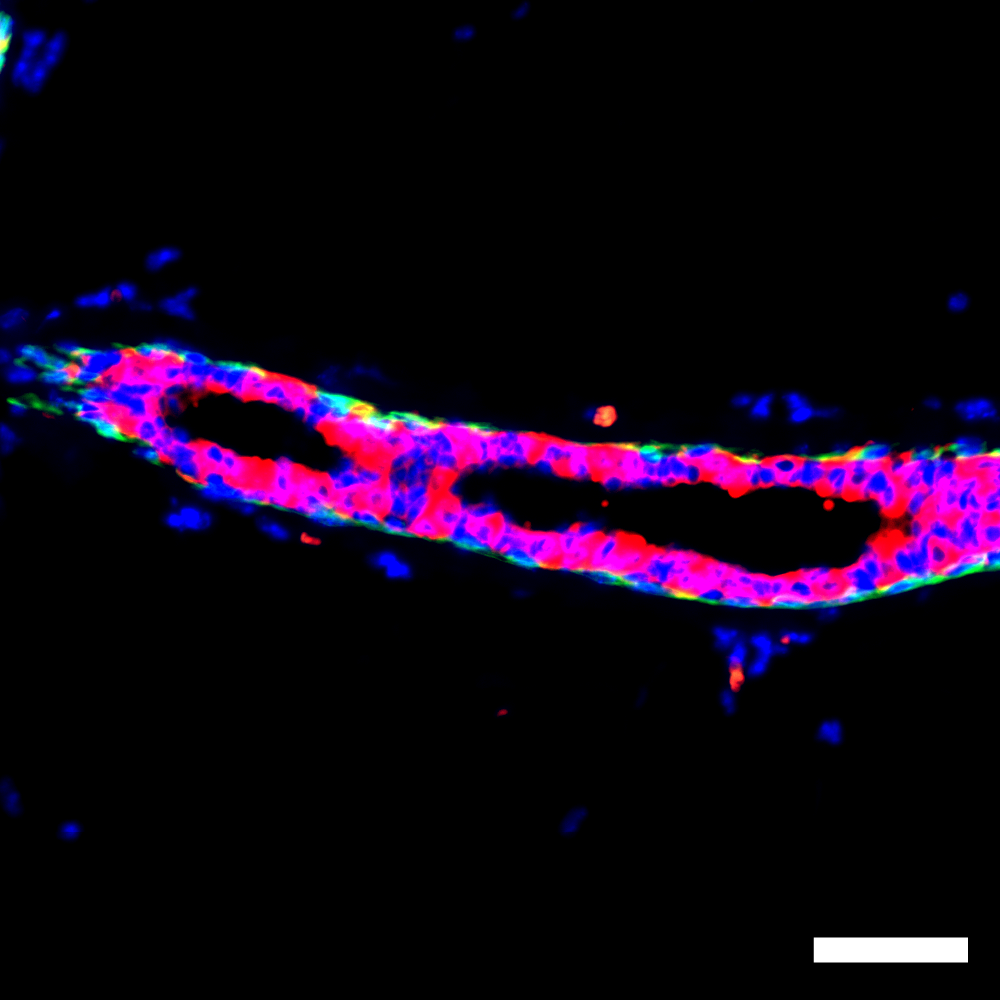

Supplement: Supplementary file 9 — Source data Fig. 7 [file 44319_2025_370_MOESM9_ESM.zip › Source Data Fig 7/7B/L12KO (LATS12ff;lslEYFP;K8Cre) + VT104 K14 K8.tif]

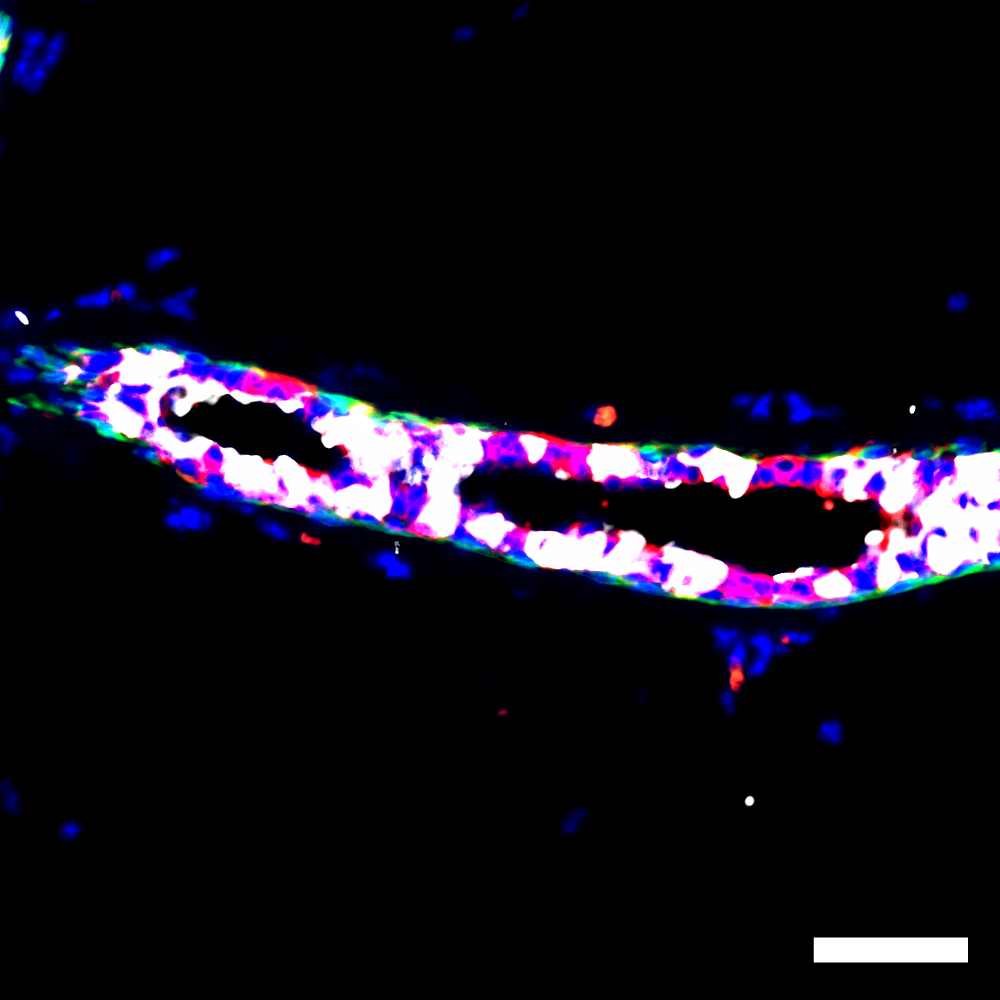

Supplement: Supplementary file 9 — Source data Fig. 7 [file 44319_2025_370_MOESM9_ESM.zip › Source Data Fig 7/7B/L12KO (LATS12ff;lslEYFP;K8Cre) + VT104 K14 K8 YFP.tif]

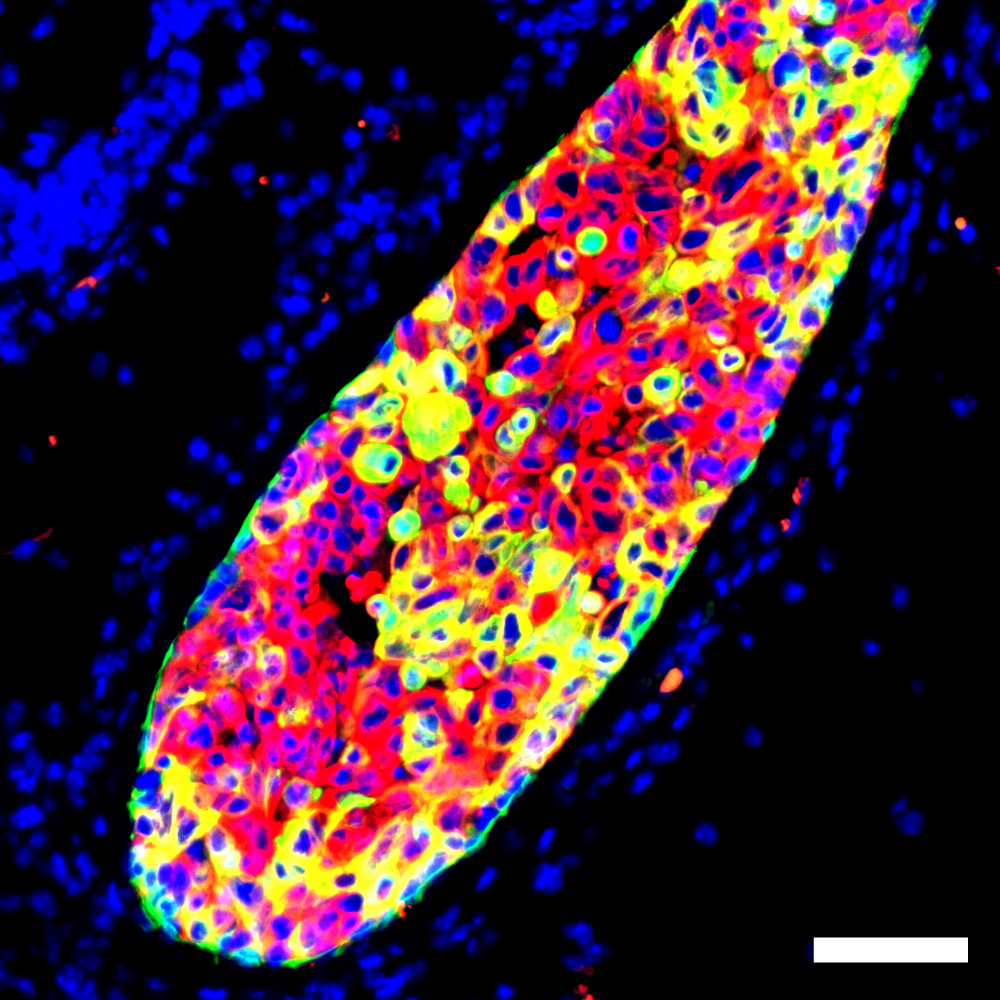

Supplement: Supplementary file 9 — Source data Fig. 7 [file 44319_2025_370_MOESM9_ESM.zip › Source Data Fig 7/7B/L12KO (LATS12ff;lslEYFP;K8Cre) + Vehicle K14 K8.tif]

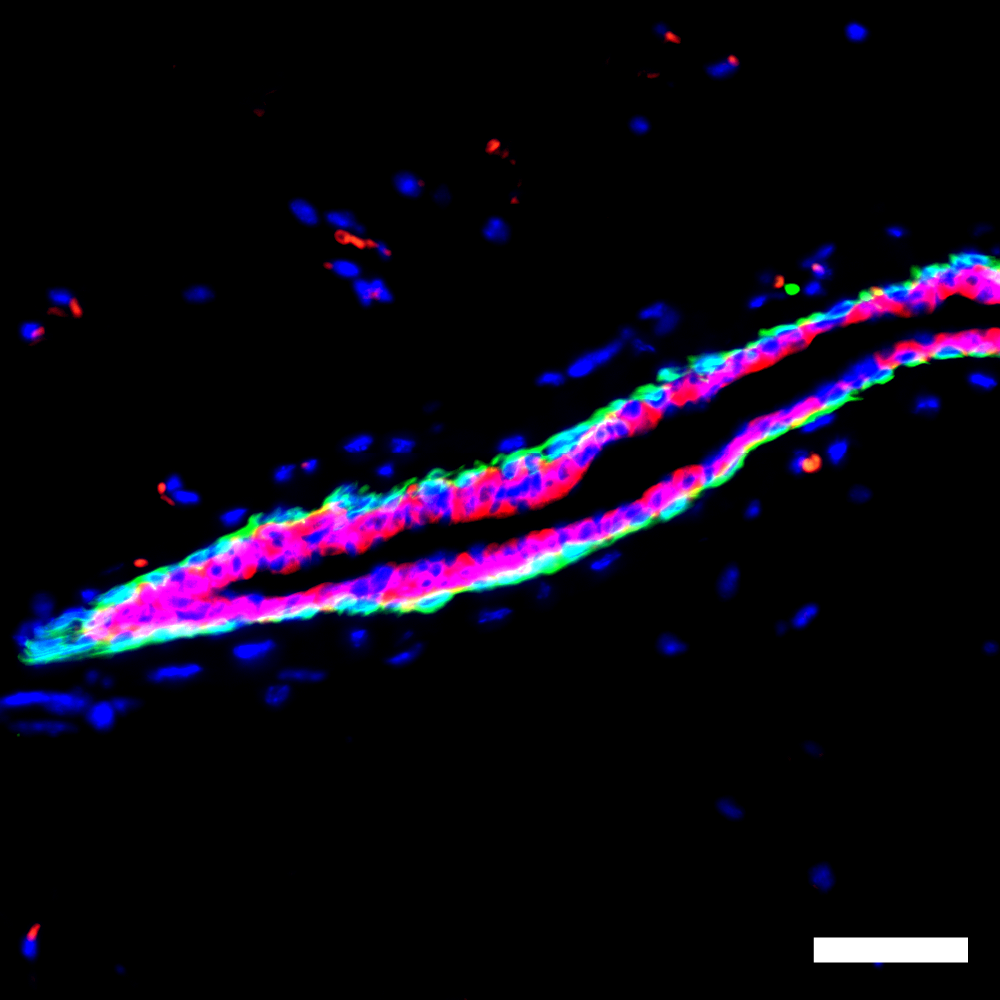

Supplement: Supplementary file 9 — Source data Fig. 7 [file 44319_2025_370_MOESM9_ESM.zip › Source Data Fig 7/7B/CTL (LATS12ff;lslEYFP;NoCre) + VT104 K14 K8.tif]

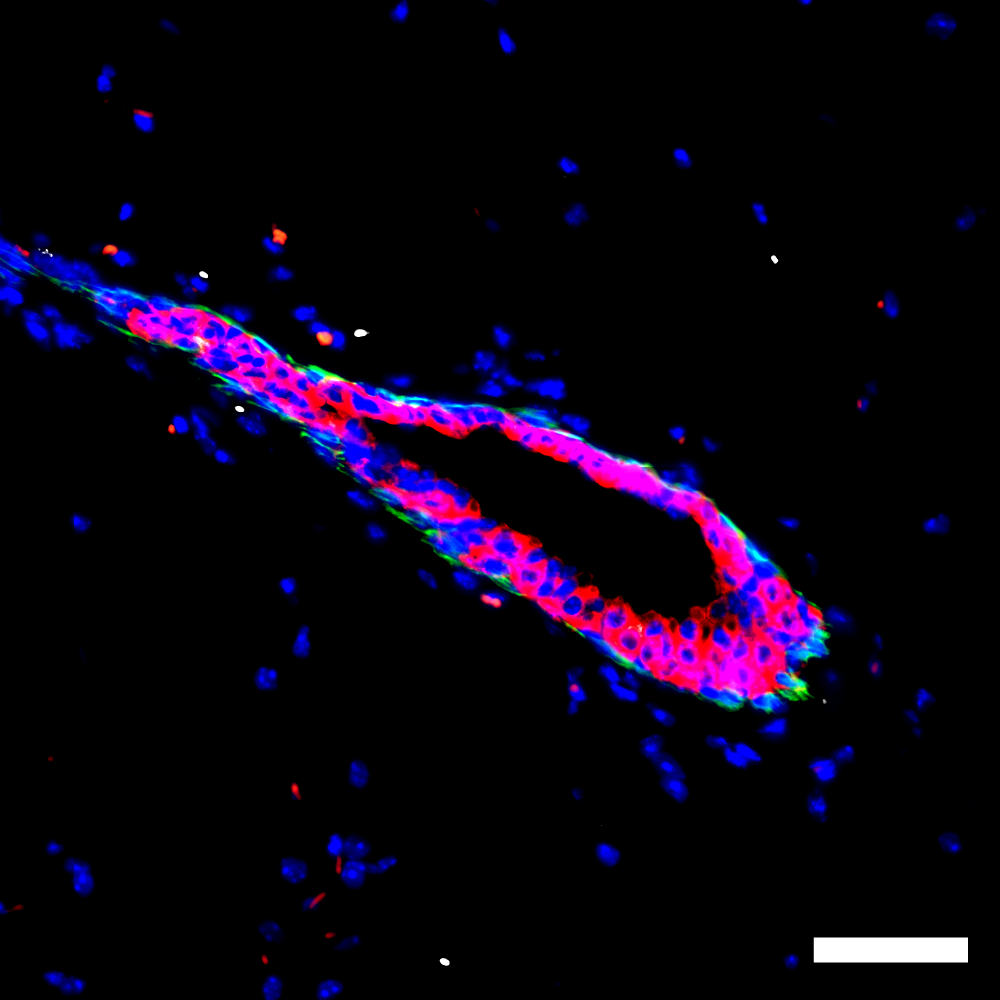

Supplement: Supplementary file 9 — Source data Fig. 7 [file 44319_2025_370_MOESM9_ESM.zip › Source Data Fig 7/7B/CTL (LATS12ff;lslEYFP;NoCre) + Vehicle K14 K8 YFP.tif]

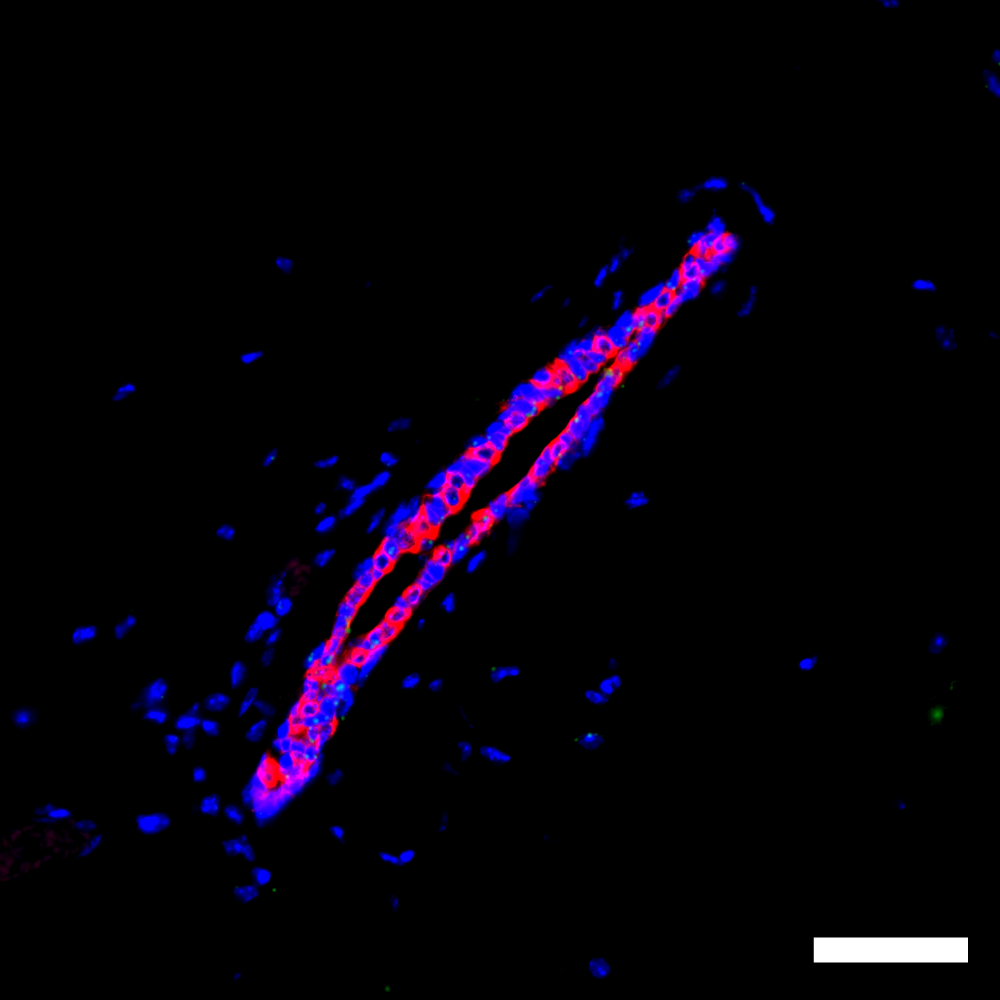

Supplement: Supplementary file 9 — Source data Fig. 7 [file 44319_2025_370_MOESM9_ESM.zip › Source Data Fig 7/7E/CTL (LATS12ff;lslEYFP;NoCre) + Vehicle K8 Tgfb2rna.tif]

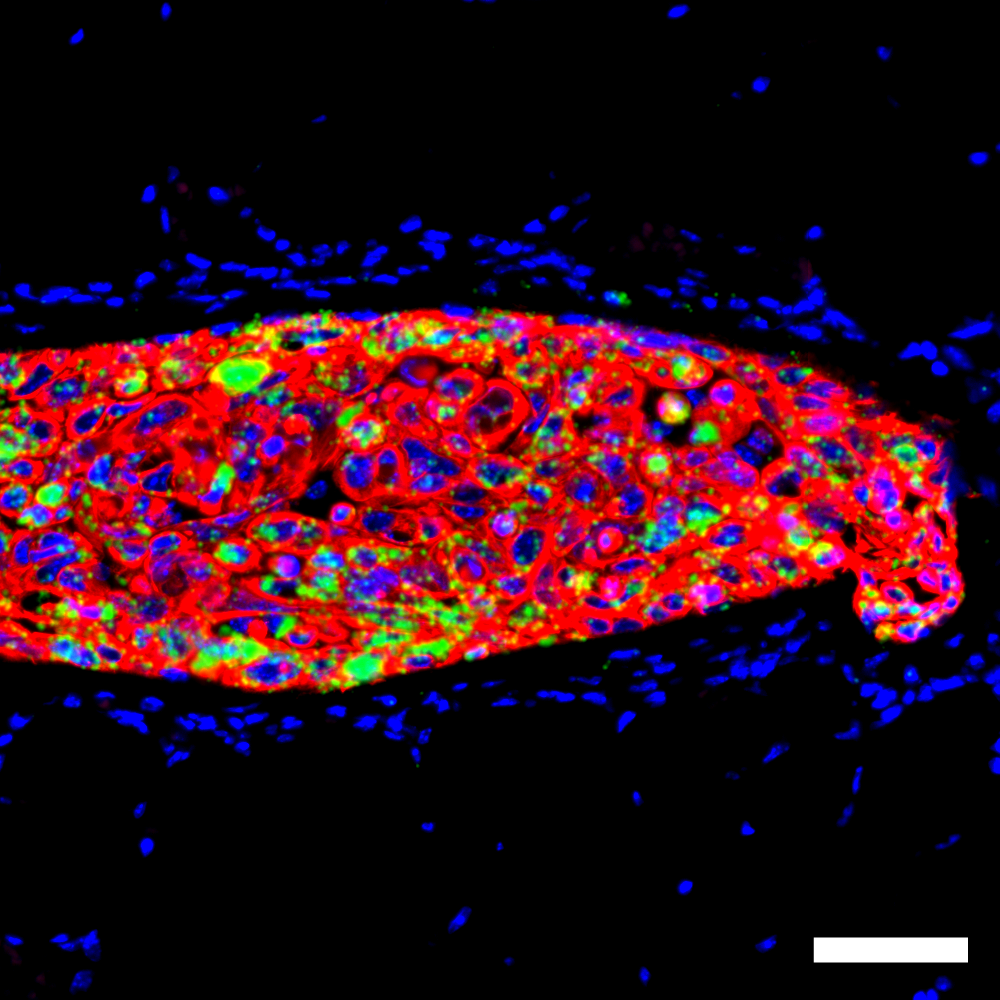

Supplement: Supplementary file 9 — Source data Fig. 7 [file 44319_2025_370_MOESM9_ESM.zip › Source Data Fig 7/7E/L12KO (LATS12ff;lslEYFP;K8Cre) + Vehicle K8 Tgfb2rna.tif]

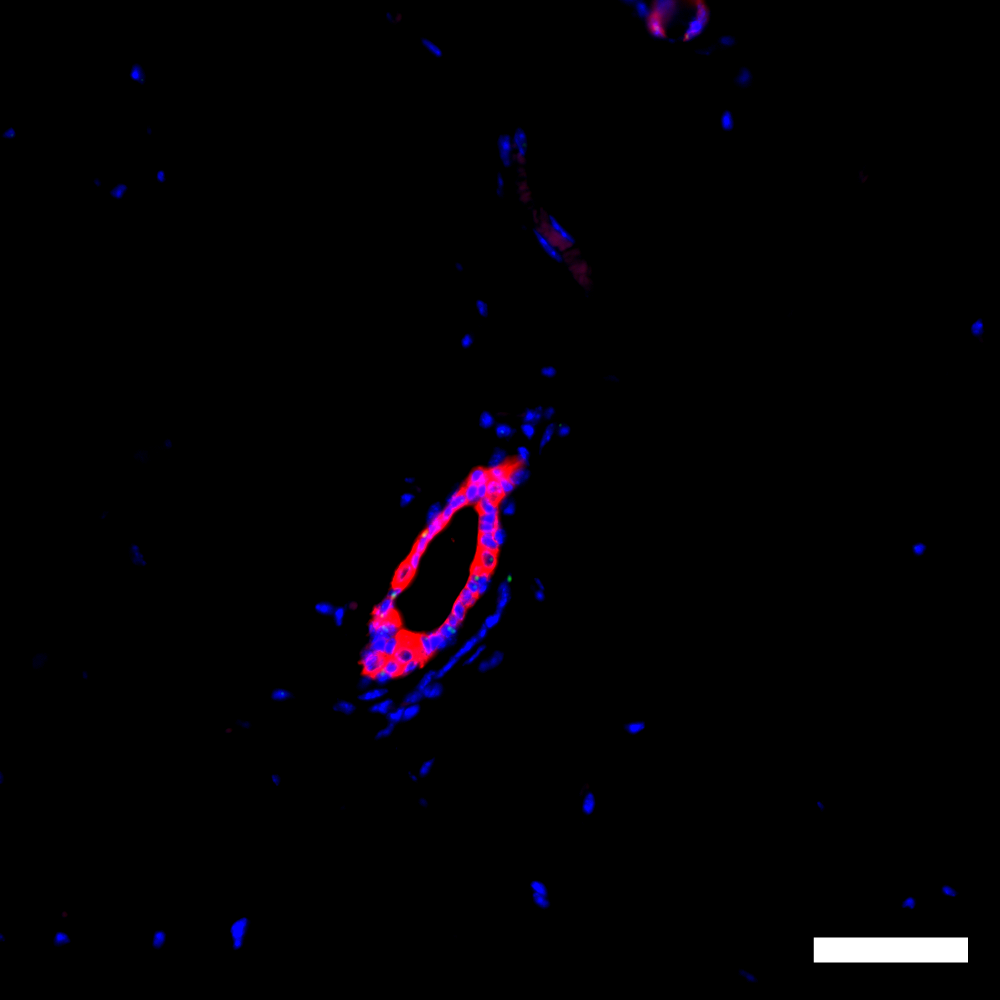

Supplement: Supplementary file 9 — Source data Fig. 7 [file 44319_2025_370_MOESM9_ESM.zip › Source Data Fig 7/7E/CTL (LATS12ff;lslEYFP;NoCre) + VT104 K8 Tgfb2rna.tif]

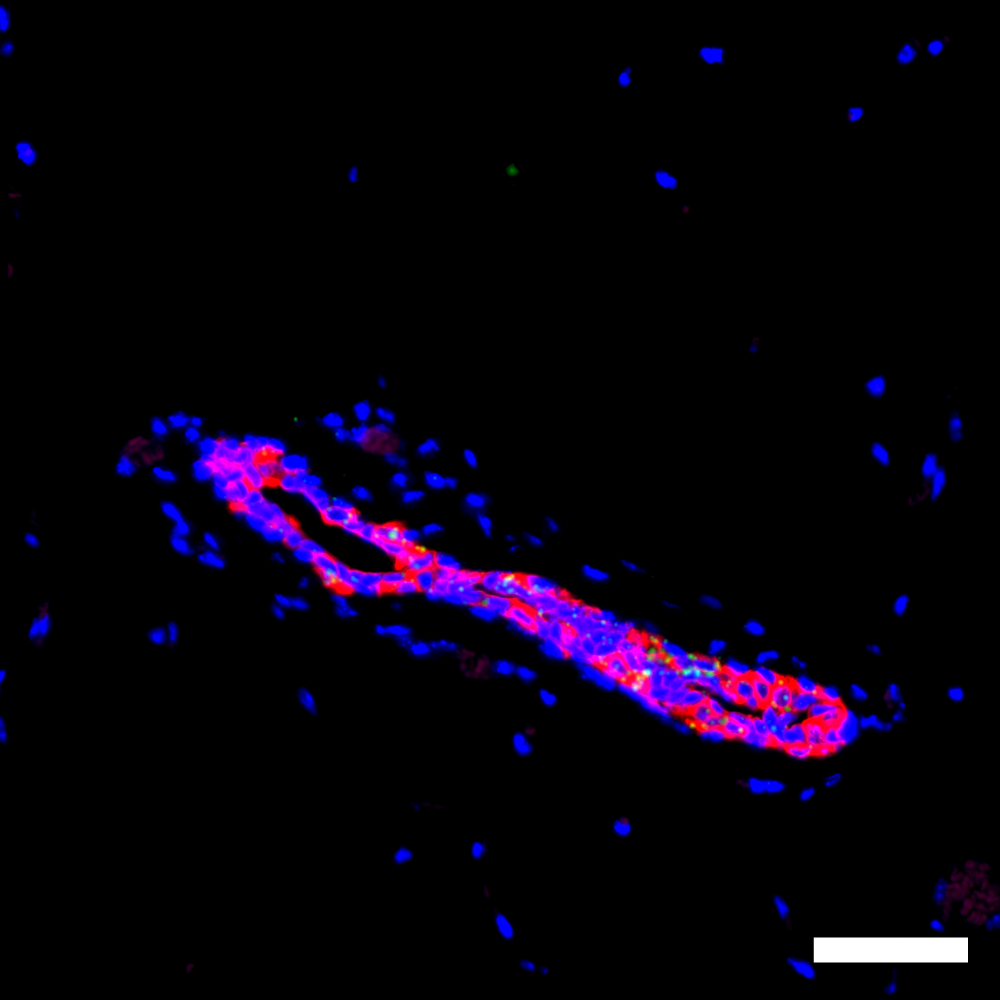

Supplement: Supplementary file 9 — Source data Fig. 7 [file 44319_2025_370_MOESM9_ESM.zip › Source Data Fig 7/7E/L12KO (LATS12ff;lslEYFP;K8Cre) + VT104 K8 Tgfb2rna.tif]

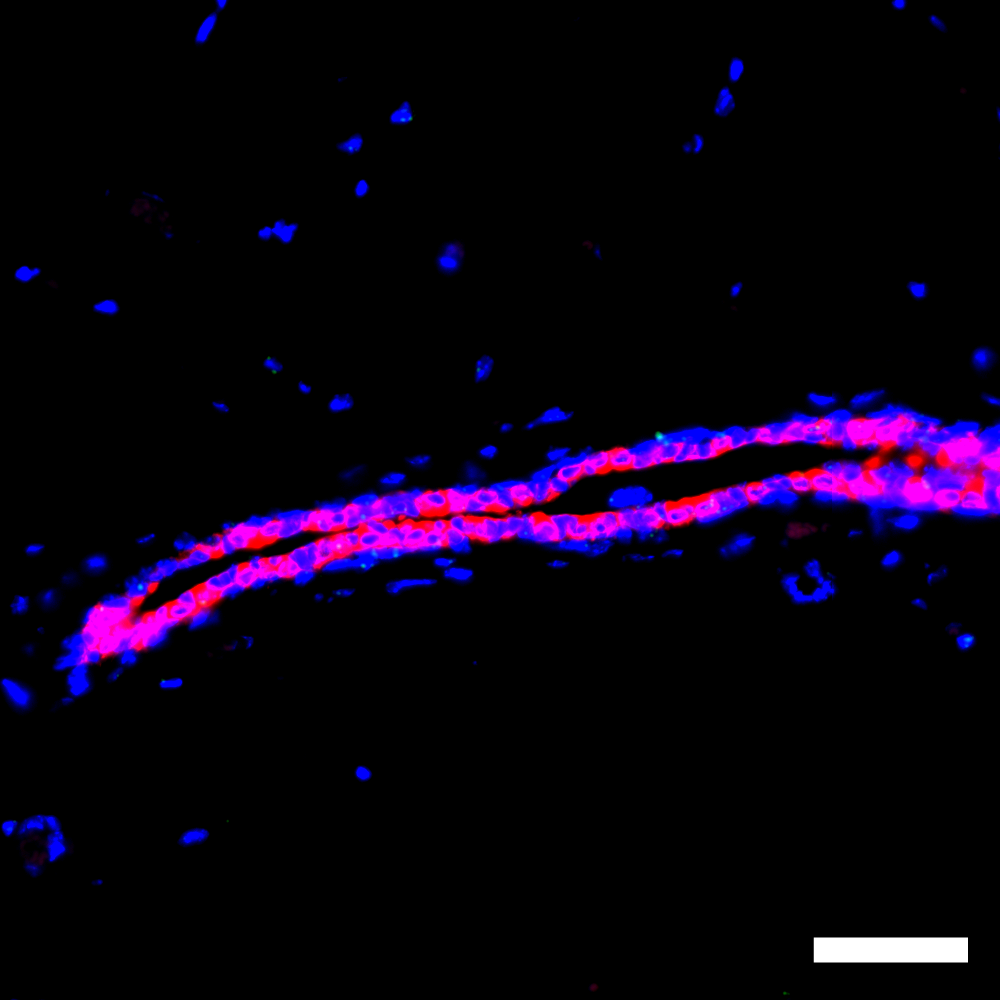

Supplement: Supplementary file 9 — Source data Fig. 7 [file 44319_2025_370_MOESM9_ESM.zip › Source Data Fig 7/7G/CTL (LATS12ff;lslEYFP;NoCre) + VT104 K8 Csf1rna.tif]

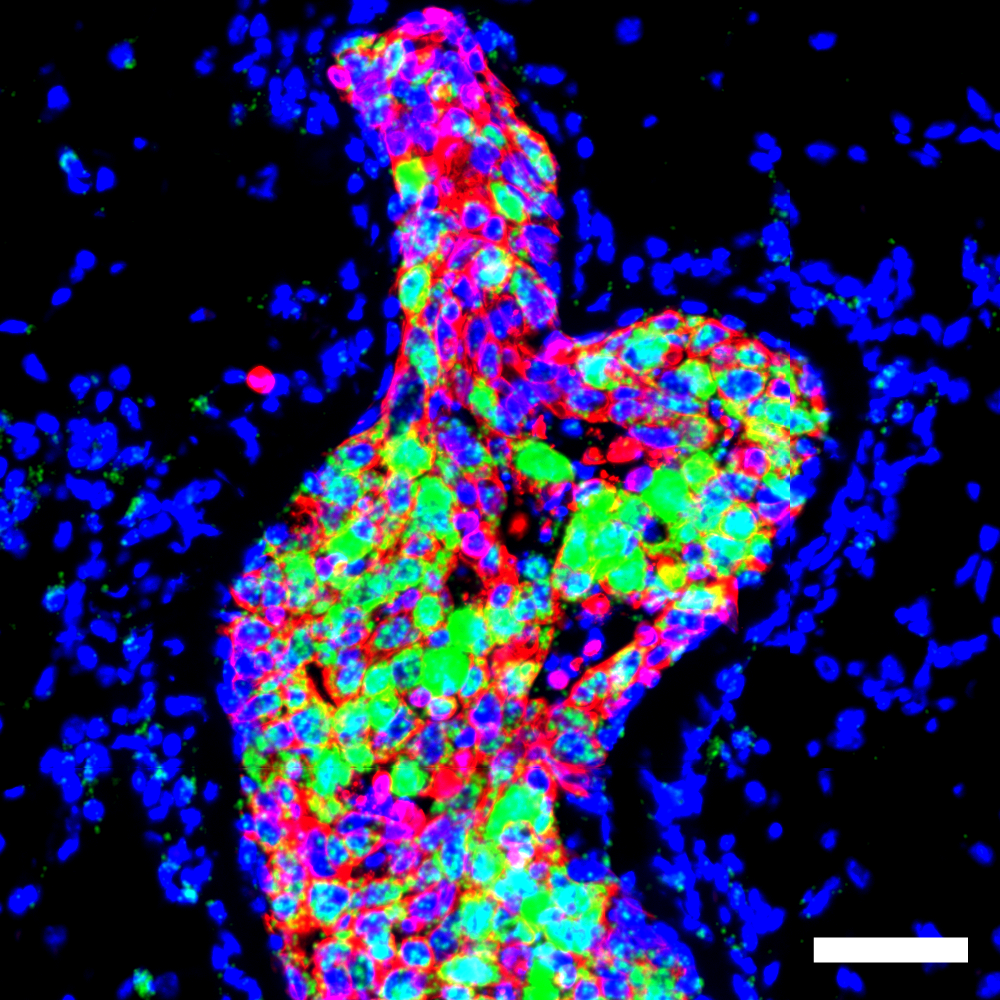

Supplement: Supplementary file 9 — Source data Fig. 7 [file 44319_2025_370_MOESM9_ESM.zip › Source Data Fig 7/7G/L12KO (LATS12ff;lslEYFP;K8Cre) + Vehicle K8 Csf1rna.tif]

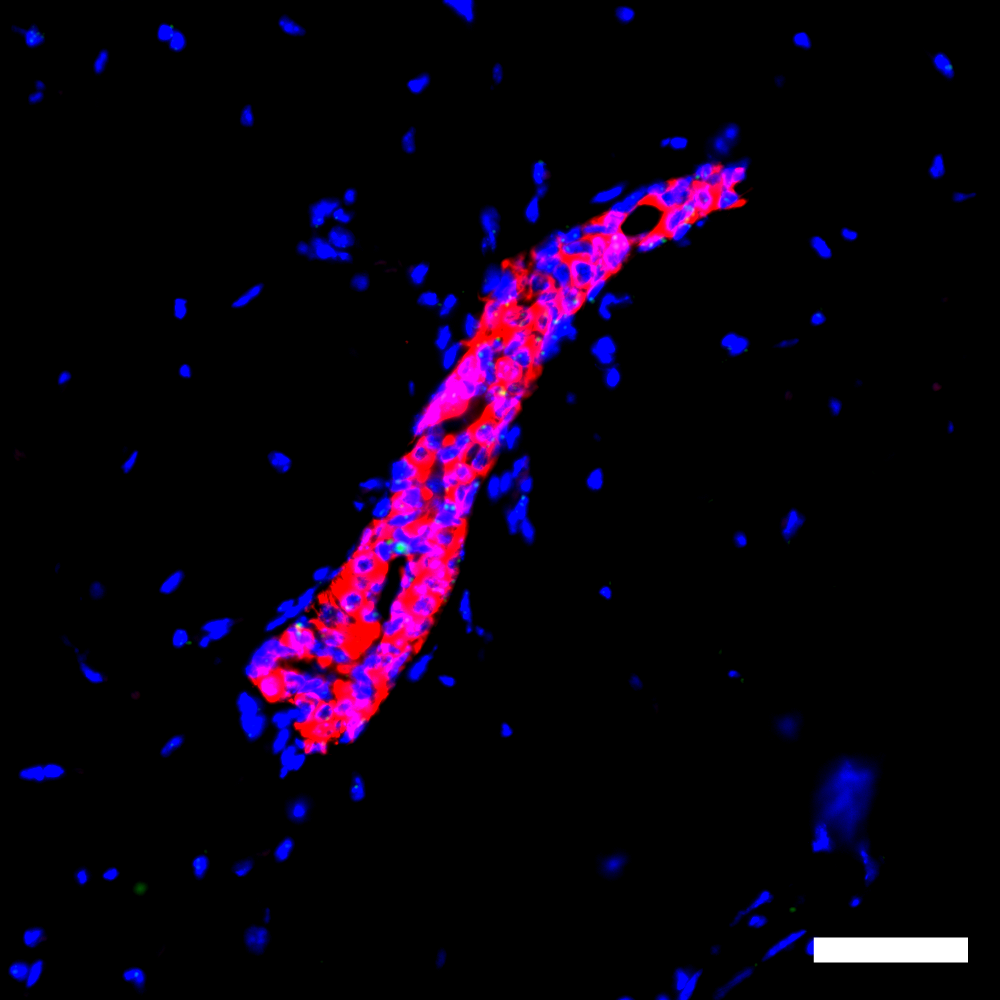

Supplement: Supplementary file 9 — Source data Fig. 7 [file 44319_2025_370_MOESM9_ESM.zip › Source Data Fig 7/7G/L12KO (LATS12ff;lslEYFP;K8Cre) + VT104 K8 Csf1rna.tif]

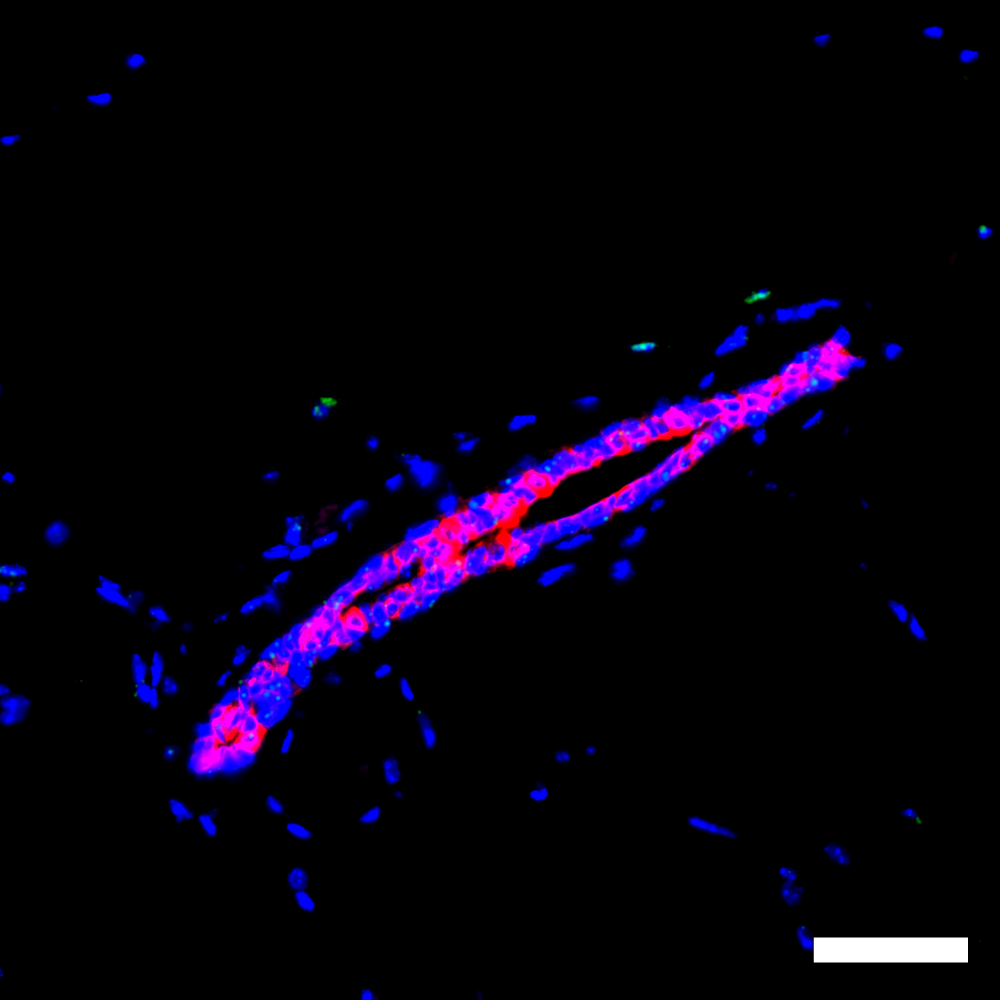

Supplement: Supplementary file 9 — Source data Fig. 7 [file 44319_2025_370_MOESM9_ESM.zip › Source Data Fig 7/7G/CTL (LATS12ff;lslEYFP;NoCre) + Vehicle K8 Csf1rna.tif]

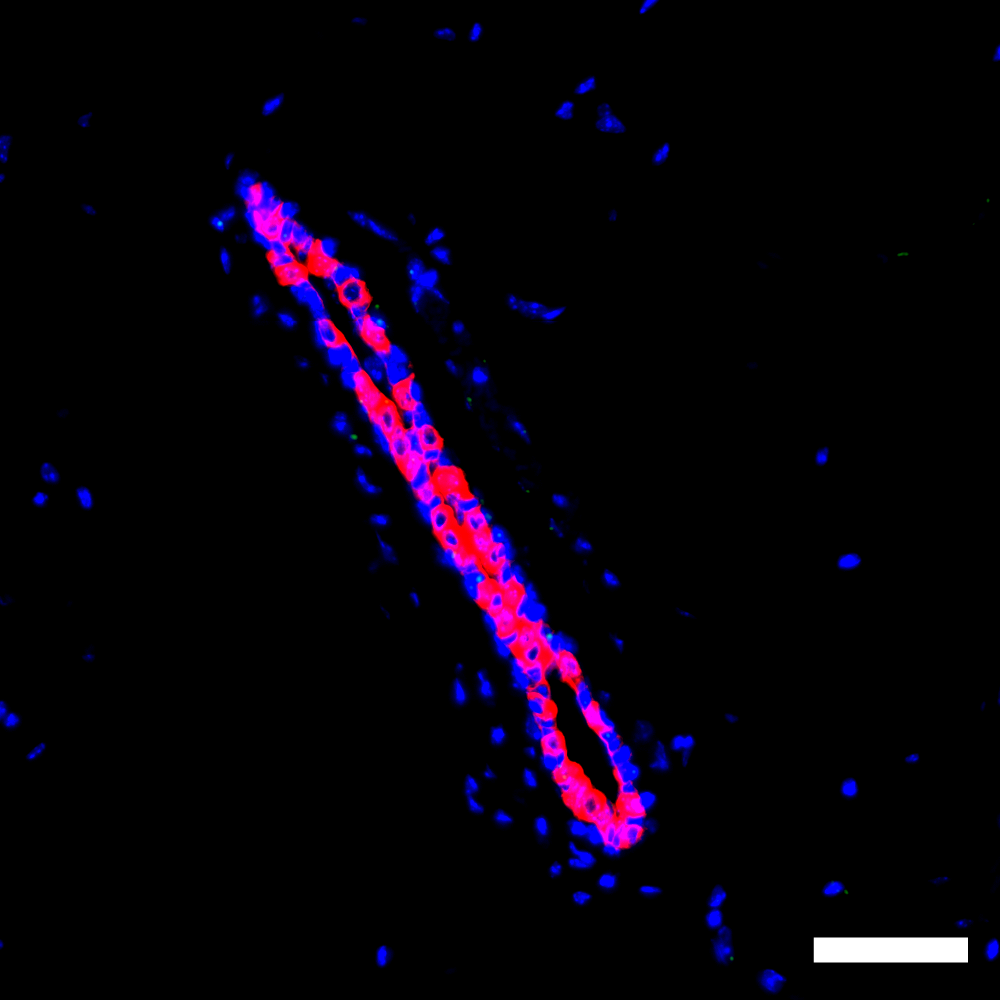

Supplement: Supplementary file 9 — Source data Fig. 7 [file 44319_2025_370_MOESM9_ESM.zip › Source Data Fig 7/7F/CTL (LATS12ff;lslEYFP;NoCre) + VT104 K8 Pdgfbrna.tif]

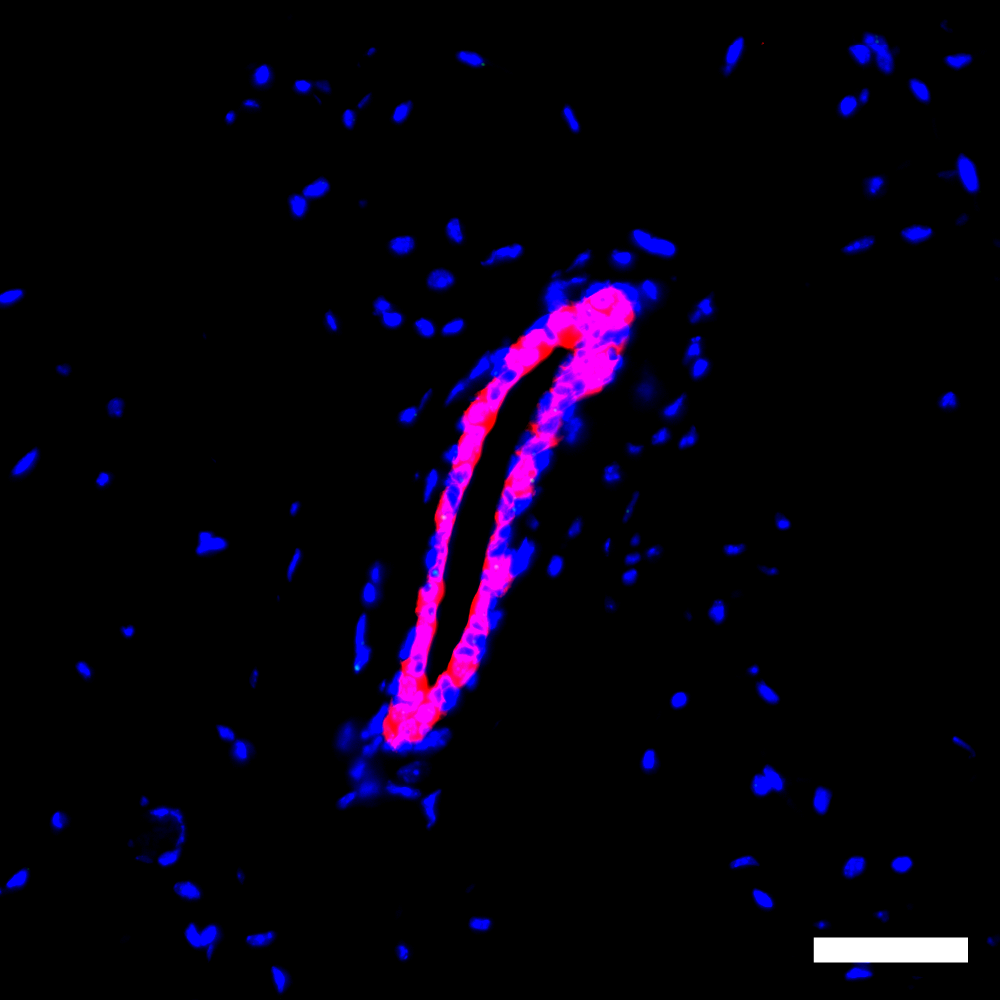

Supplement: Supplementary file 9 — Source data Fig. 7 [file 44319_2025_370_MOESM9_ESM.zip › Source Data Fig 7/7F/CTL (LATS12ff;lslEYFP;NoCre) + Vehicle K8 Pdgfbrna.tif]

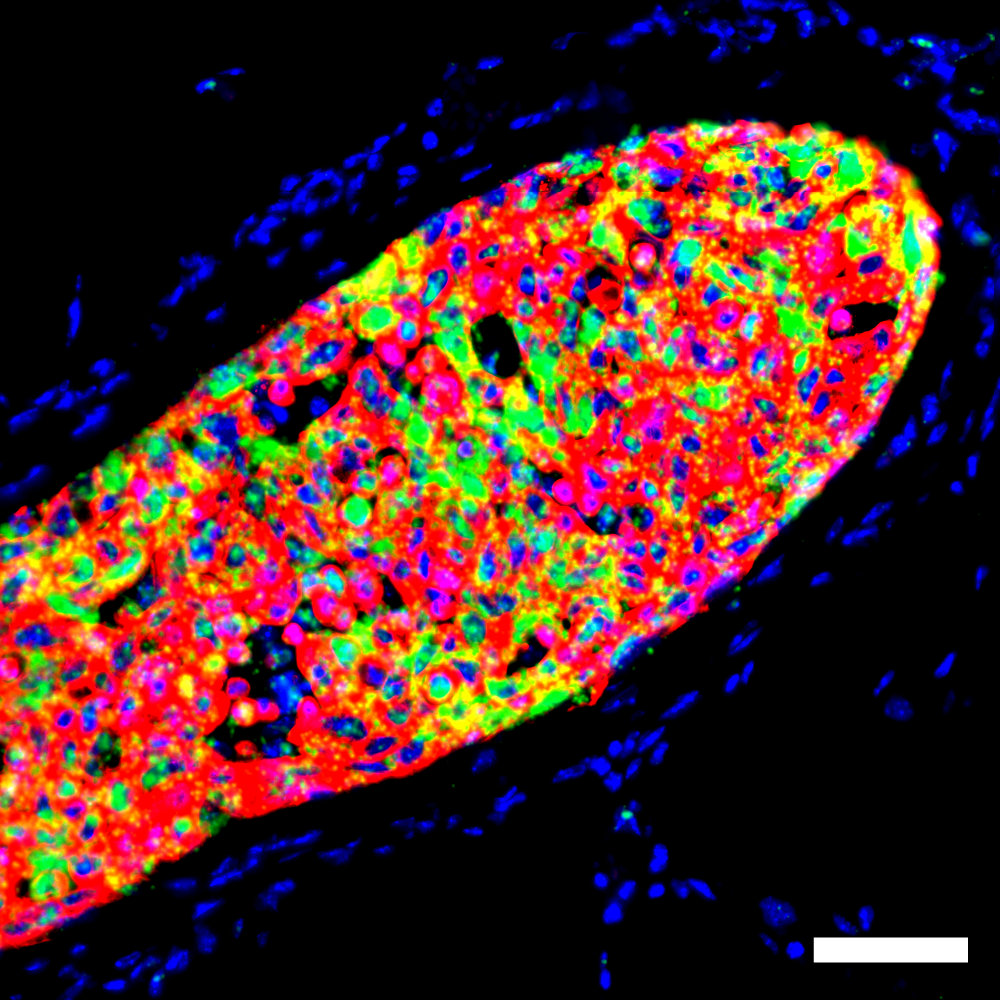

Supplement: Supplementary file 9 — Source data Fig. 7 [file 44319_2025_370_MOESM9_ESM.zip › Source Data Fig 7/7F/L12KO (LATS12ff;lslEYFP;K8Cre) + Vehicle K8 Pdgfbrna.tif]

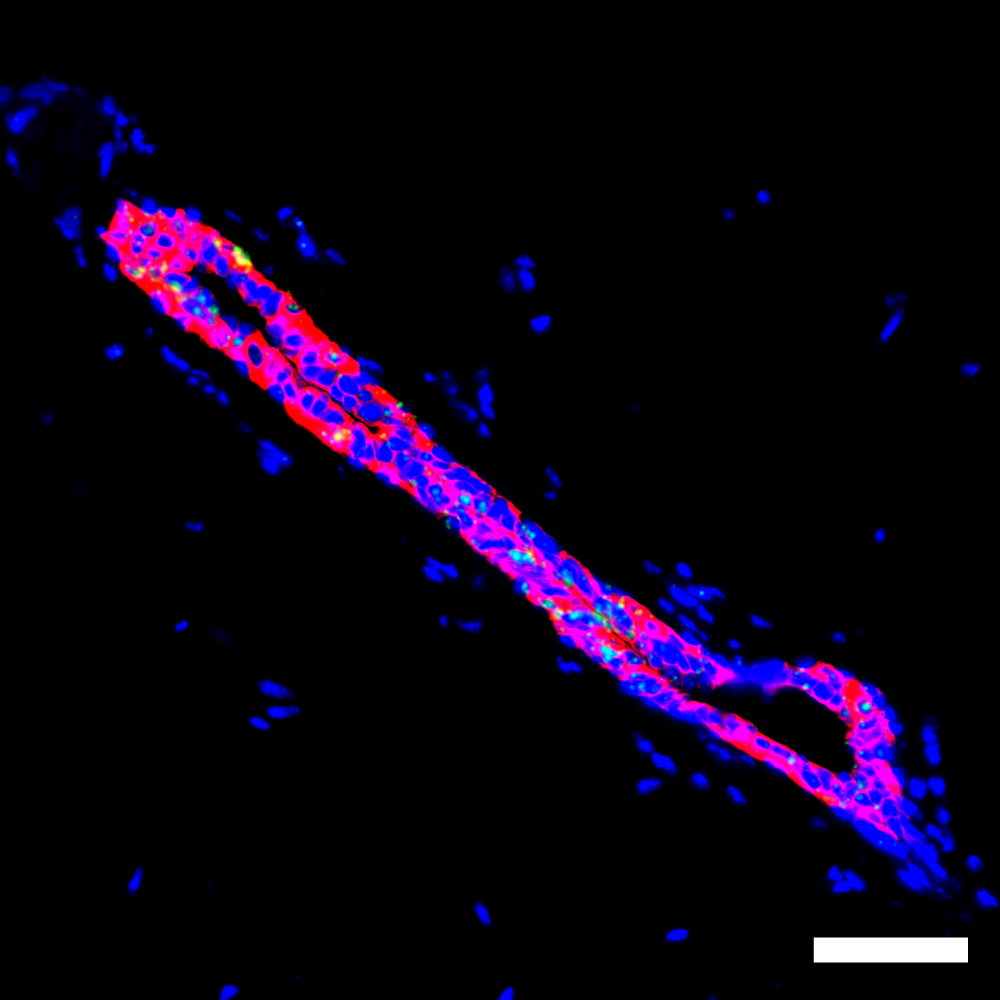

Supplement: Supplementary file 9 — Source data Fig. 7 [file 44319_2025_370_MOESM9_ESM.zip › Source Data Fig 7/7F/L12KO (LATS12ff;lslEYFP;K8Cre) + VT104 K8 Pdgfbrna.tif]

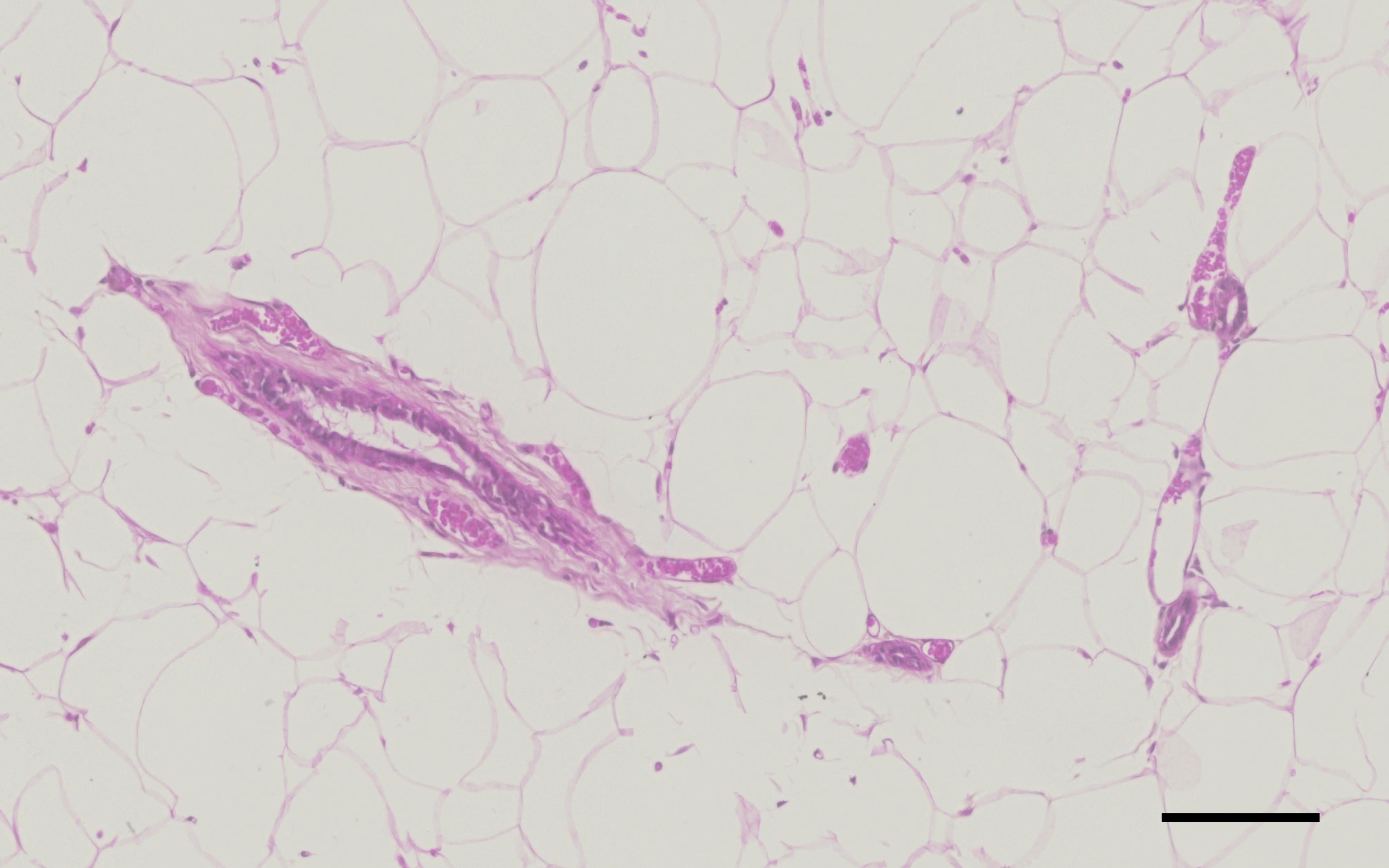

Supplement: Supplementary file 9 — Source data Fig. 7 [file 44319_2025_370_MOESM9_ESM.zip › Source Data Fig 7/7A/L12KO + VT104.tif]

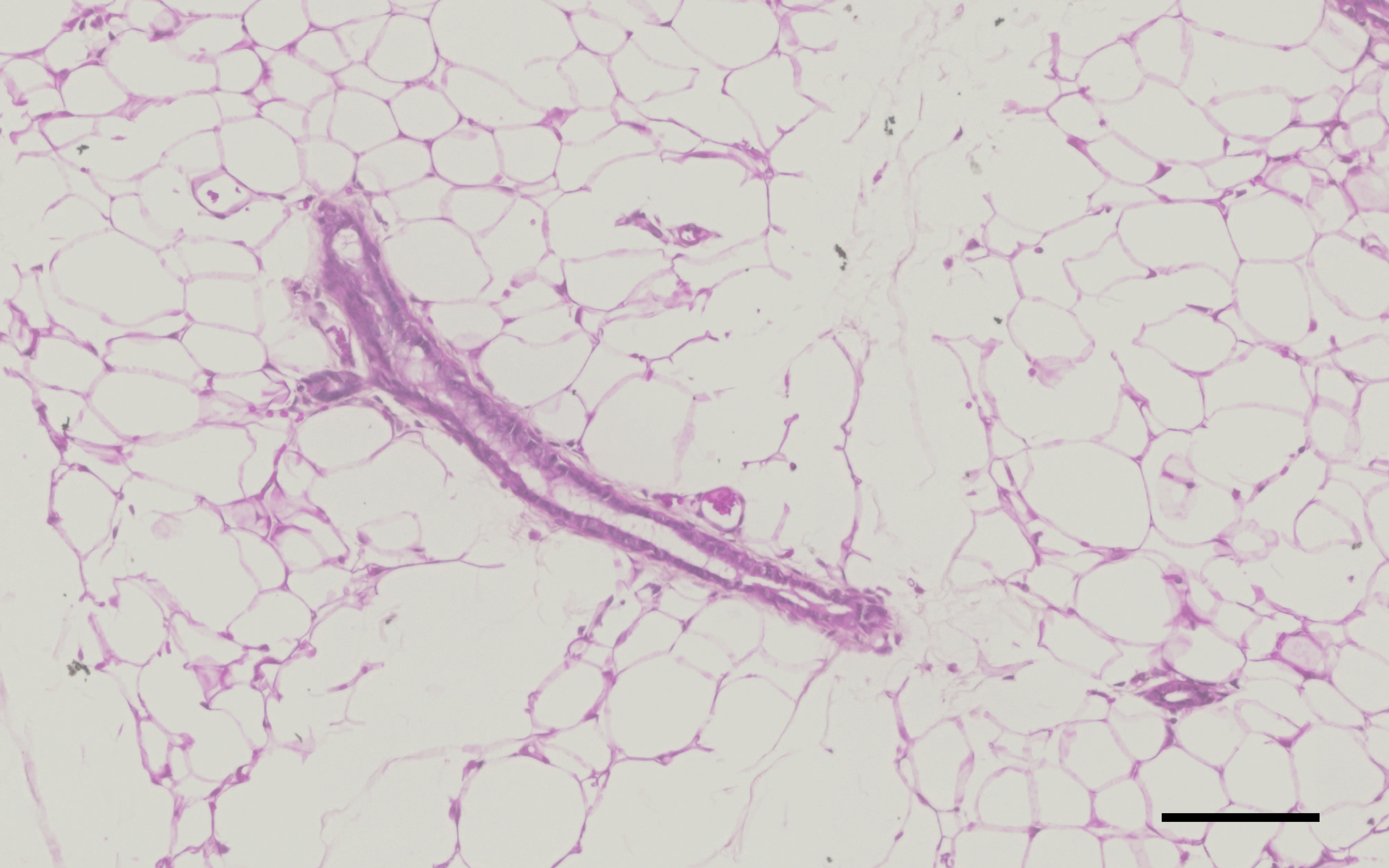

Supplement: Supplementary file 9 — Source data Fig. 7 [file 44319_2025_370_MOESM9_ESM.zip › Source Data Fig 7/7A/CTL + VT104.tif]

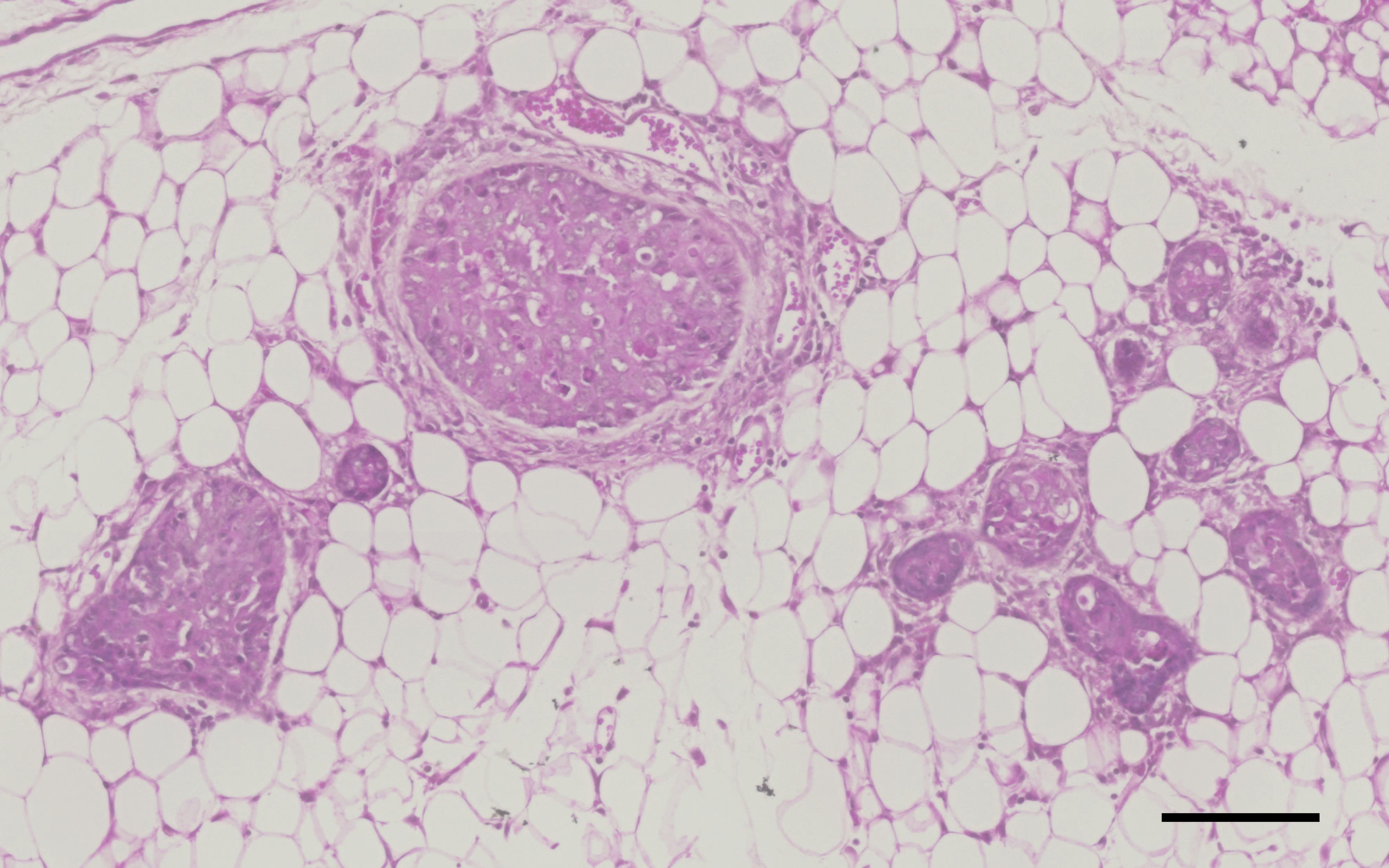

Supplement: Supplementary file 9 — Source data Fig. 7 [file 44319_2025_370_MOESM9_ESM.zip › Source Data Fig 7/7A/L12KO + Vehicle.tif]

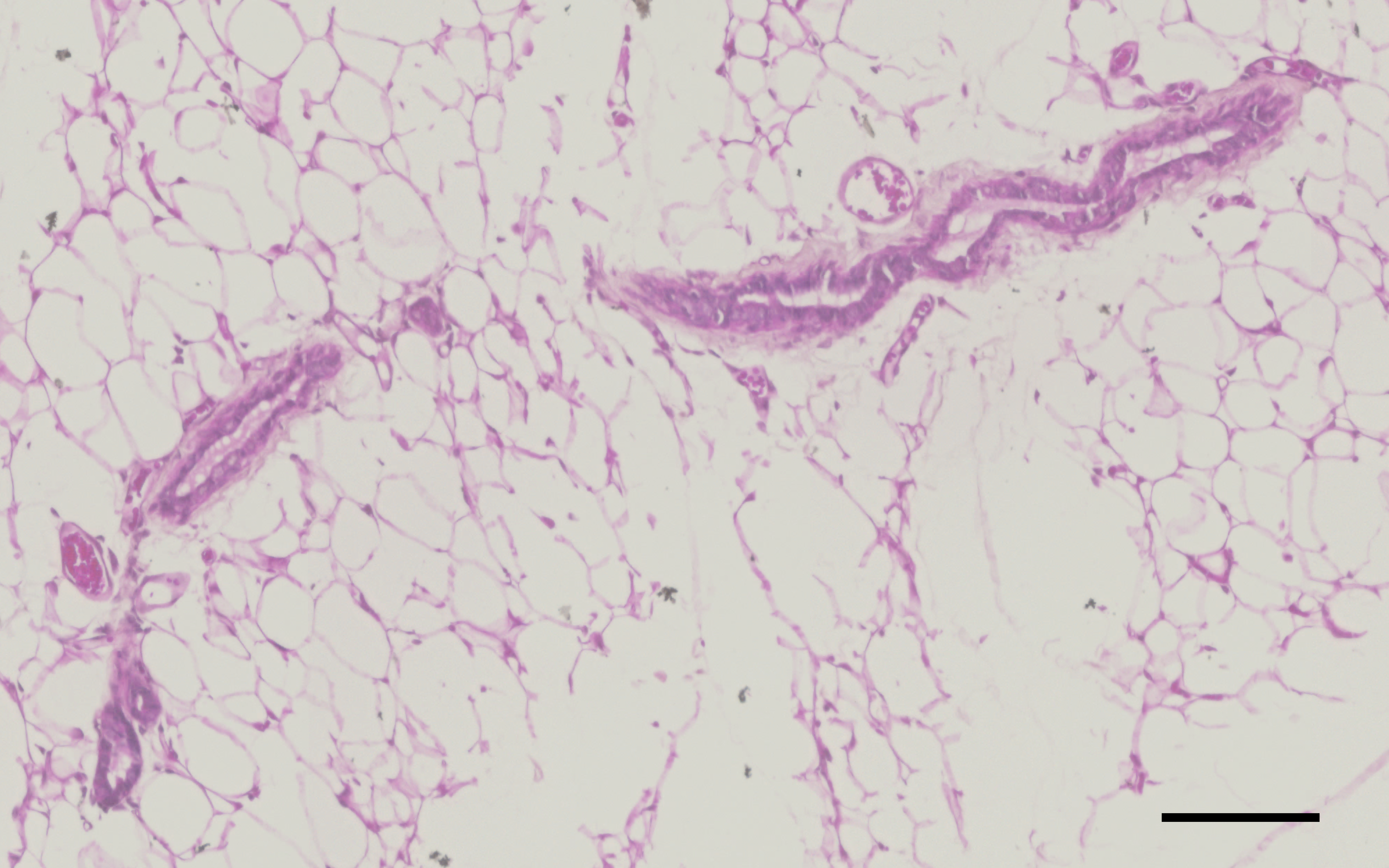

Supplement: Supplementary file 9 — Source data Fig. 7 [file 44319_2025_370_MOESM9_ESM.zip › Source Data Fig 7/7A/CTL + Vehicle.tif]
