# Supplementary material for: LATS1/2 inactivation in the mammary epithelium drives the evolution of a tumor-associated niche
Source: EMBO Rep. 2025 Feb 14;26(6):1472–503. doi: 10.1038/s44319-025-00370-3 (PMC11933708; doi:10.1038/s44319-025-00370-3)
Supplement: Supplementary file 10 — Expanded View Figures [file 44319_2025_370_MOESM10_ESM.pdf]

Expanded View Figures

Figure EV1. Epithelial and stromal alterations in basal-like mammary carcinomas driven by LATS1/2 deletion.

(**A**) Immunofluorescence staining for DAPI, KRT14, and KRT8 in CTL and LATS1/2-KO mammary glands (Scale bar, 50 µm) and quantification of nuclei counts per area in the mammary stroma ($n = 6$ CTL mice, $n = 6$ L1/2-KO mice, 5 regions analyzed per mouse. Data are shown with mean ± SEM. Two-tailed unpaired T-test). (**B**) Heatmap of the top 10 differentially expressed genes in each cluster (ranked by Log2FC and using genes expressed in at least 50% of cells in the cluster). Note that if a gene is duplicated in the top 10 genes of two separate clusters, it is only shown once. (**C**) Violin plot of EYFP expression across all clusters ($n = 3496$ cells for C1, $n = 2666$ cells for C2, $n = 2458$ cells for C3, $n = 1607$ cells for C4, $n = 1483$ cells for C5, $n = 1156$ cells for C6, $n = 1031$ cells for C7, $n = 928$ cells for C8, $n = 799$ cells for C9, $n = 611$ cells for C10, $n = 603$ cells for C11, $n = 440$ cells for C12, $n = 417$ cells for C13, $n = 369$ cells for C14, $n = 140$ cells for C15). (**D**) UMAP of cell groups specified for CellChat analyses.

© The Author(s)

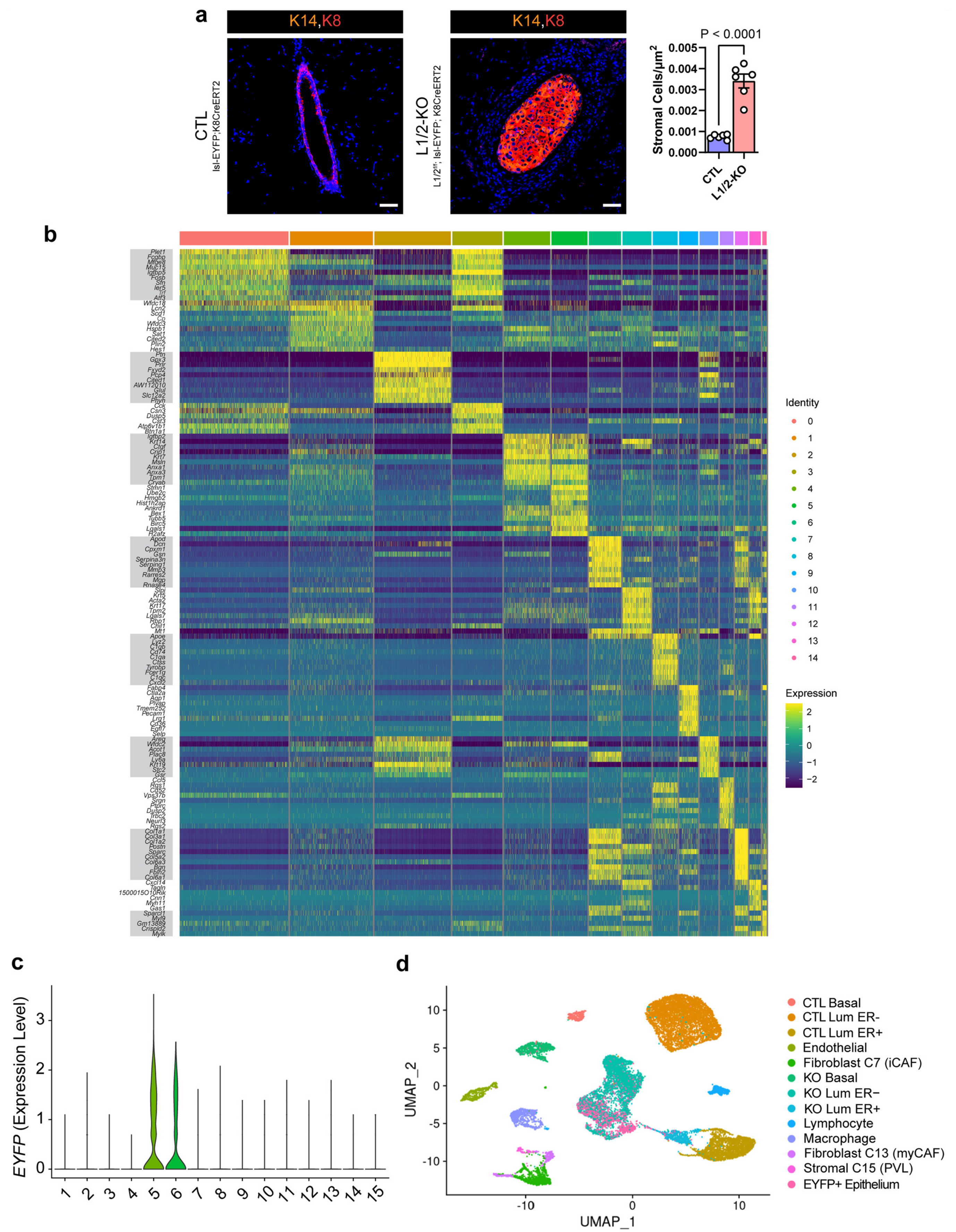
a
K14,K8
K14,K8
CTL
lsl-EYFP;K8CreERT2
L1/2-KO
L1/2$^{f/f}$; lsl-EYFP; K8CreERT2
P < 0.0001
Stromal Cells/µm^2
0.005
0.004
0.003
0.002
0.001
0.000
CTL
L1/2-KO
b
Identity
0
1
2
3
4
5
6
7
8
9
10
11
12
13
14
Expression
2
1
0
−1
−2
c
EYFP (Expression Level)
3
2
1
0
1 2 3 4 5 6 7 8 9 10 11 12 13 14 15
d
UMAP_2
UMAP_1
10
5
0
-5
-10
-10
0
10
CTL Basal
CTL Lum ER-
CTL Lum ER+
Endothelial
Fibroblast C7 (iCAF)
KO Basal
KO Lum ER−
KO Lum ER+
Lymphocyte
Macrophage
Fibroblast C13 (myCAF)
Stromal C15 (PVL)
EYFP+ Epithelium

 © The Author(s)

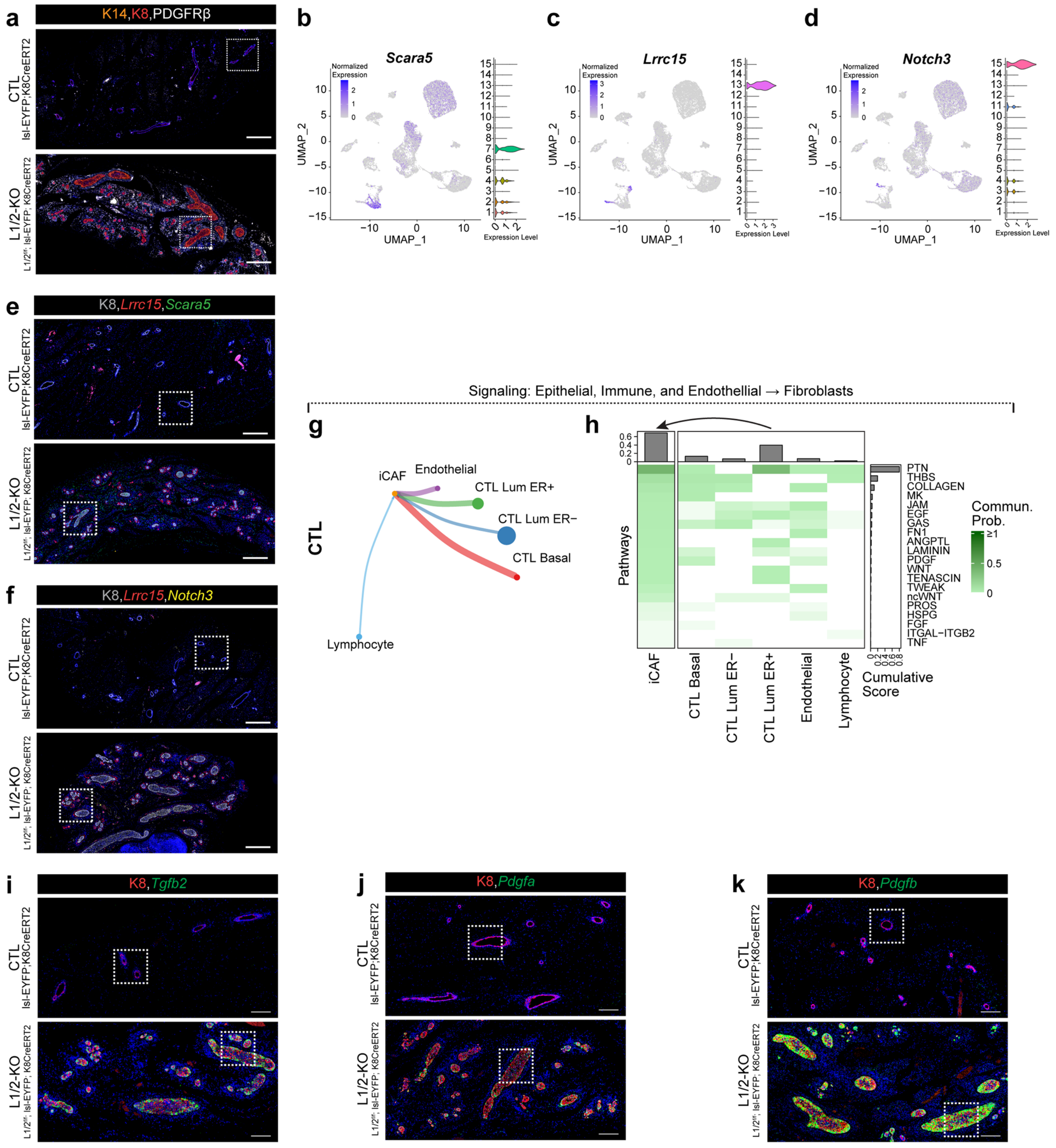
a
K14,K8,PDGFRβ
CTL
lsl-EYFP;K8CreERT2
L1/2-KO
L1/2f/f; lsl-EYFP; K8CreERT2
b
Scara5
c
Lrrc15
d
Notch3
Normalized Expression
UMAP_1
UMAP_2
Expression Level
e
K8,Lrrc15,Scara5
f
K8,Lrrc15,Notch3
Signaling: Epithelial, Immune, and Endothellial → Fibroblasts
g
CTL
iCAF
Endothelial
CTL Lum ER+
CTL Lum ER−
CTL Basal
Lymphocyte
h
Pathways
PTN
THBS
COLLAGEN
MK
JAM
EGF
GAS
FN1
ANGPTL
LAMININ
PDGF
WNT
TENASCIN
TWEAK
ncWNT
PROS
HSPG
FGF
ITGAL−ITGB2
TNF
Commun. Prob.
iCAF
CTL Basal
CTL Lum ER−
CTL Lum ER+
Endothelial
Lymphocyte
Cumulative Score
i
K8,Tgfb2
j
K8,Pdgfa
k
K8,Pdgfb

Figure EV2. Accumulation of cancer-associated fibroblasts in the mammary stroma of mice with LATS1/2 inactivation.

(**A**) Immunofluorescence staining for KRT14, KRT8, and PDGFRβ in CTL and LATS1/2-KO mammary glands. Dashed boxes indicate subsets shown in Fig. 3A (Scale bar, 500 μm). (**B–D**) UMAPs of transcriptional markers of the iCAF (*Scara5*) (**B**), myCAF (*Lrrc15*) (**C**), and PVL (*Notch3*) (**D**) populations, along with violin plots of the expression of each gene across all clusters (For violin plots in (**B–D**), $n = 3496$ cells for C1, $n = 2666$ cells for C2, $n = 2458$ cells for C3, $n = 1607$ cells for C4, $n = 1483$ cells for C5, $n = 1156$ cells for C6, $n = 1031$ cells for C7, $n = 928$ cells for C8, $n = 799$ cells for C9, $n = 611$ cells for C10, $n = 603$ cells for C11, $n = 440$ cells for C12, $n = 417$ cells for C13, $n = 369$ cells for C14, $n = 140$ cells for C15). (**E**) RNAscope of *Lrrc15* and *Scara5* along with IF for KRT8 in CTL and LATS1/2-KO mammary glands. Dashed boxes indicate subsets shown in Fig. 3E (Scale bar, 500 μm) ($n = 3$ CTL mice, $n = 3$ L1/2-KO mice). (**F**) RNAscope of *Lrrc15* and *Notch3* along with IF for KRT8 in CTL and LATS1/2-KO mammary glands. Dashed boxes indicate subsets shown in Fig. 3F (Scale bar, 500 μm) ($n = 3$ CTL mice, $n = 3$ L1/2-KO mice). (**G**) CellChat circle plots showing communication networks from epithelial and stromal cell groups (senders) to iCAF group (receiver) in CTL mammary glands. Line width represents the total number of interactions identified. (**H**) Heatmap of communication probabilities of signaling pathways from epithelial and non-fibroblast-associated stromal cell groups to the iCAF population in CTL mammary glands. (**I–K**) RNAscope for *Tgfb2* (**I**), *Pdgfa* (**J**), and *Pdgfb* (**K**) in CTL and LATS1/2-KO mammary glands, along with IF for KRT8. Dashed boxes indicate subsets shown in Fig. 3J–L (Scale bar, 200 μm) ($n = 3$ CTL mice, $n = 3$ L1/2-KO mice each for **I–K**).

 © The Author(s)

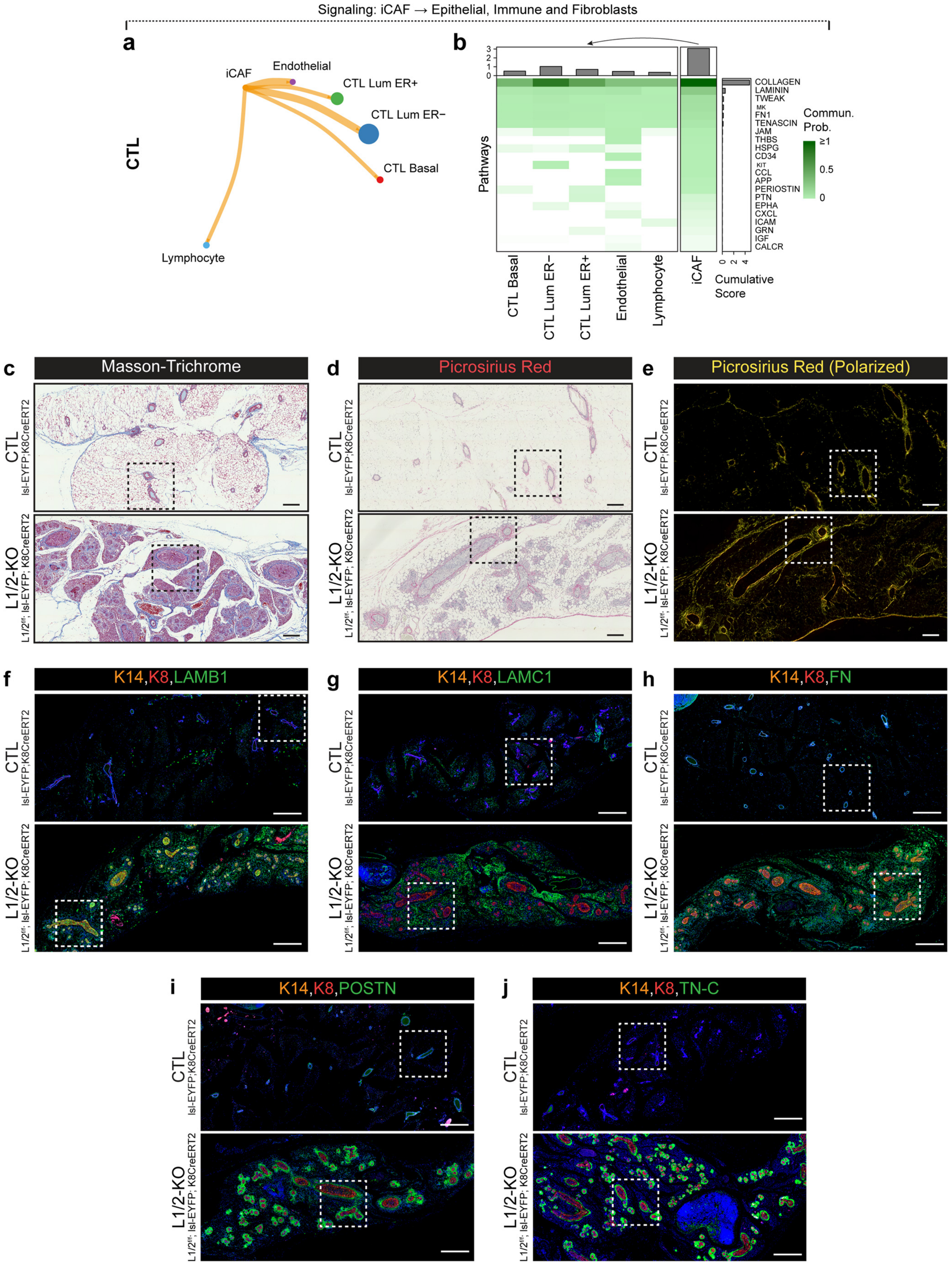
Signaling: iCAF → Epithelial, Immune and Fibroblasts
a
CTL
iCAF
Endothelial
CTL Lum ER+
CTL Lum ER−
CTL Basal
Lymphocyte
b
Pathways
COLLAGEN
LAMININ
TWEAK
MK
FN1
TENASCIN
JAM
THBS
HSPG
CD34
KIT
CCL
APP
PERIOSTIN
PTN
EPHA
CXCL
ICAM
GRN
IGF
CALCR
Commun. Prob.
≥1
0.5
0
CTL Basal
CTL Lum ER−
CTL Lum ER+
Endothelial
Lymphocyte
iCAF
Cumulative Score
c
Masson-Trichrome
d
Picrosirius Red
e
Picrosirius Red (Polarized)
CTL
lsl-EYFP;K8CreERT2
L1/2-KO
L1/2f/f; lsl-EYFP; K8CreERT2
f
K14,K8,LAMB1
g
K14,K8,LAMC1
h
K14,K8,FN
i
K14,K8,POSTN
j
K14,K8,TN-C

Figure EV3. Mammary carcinomas driven by LATS1/2 deletion display deposition of extracellular matrix proteins.

(**A**) CellChat circle plots showing communication networks from the iCAF group (senders) to epithelial and stromal cell groups (receivers) in CTL mammary glands. Line widths represent the total number of interactions identified. (**B**) Heatmap of communication probabilities of signaling pathways from the iCAF group to epithelial and stromal cell groups in CTL mammary glands. (**C**) Masson-Trichrome staining of CTL and LATS1/2-KO mammary glands (Scale bar 200 μm) ($n = 6$ CTL mice, $n = 4$ L1/2-KO mice from two experiments). (**D–E**) Picrosirius red staining in CTL and LATS1/2-KO mammary glands using brightfield (**D**) and polarizing (**E**) light. (Scale bar 200 μm) ($n = 4$ CTL mice, $n = 6$ L1/2-KO mice). (**F–J**) Immunofluorescence staining for KRT14, KRT8, and Laminin β1 (**F**), Laminin γ1 (**G**), Fibronectin (**H**), Periostin (**I**), and Tenascin C (**J**) in CTL and LATS1/2-KO mammary glands (Scale bar 500 μm) ($n = 6$ CTL mice, $n = 6$ L1/2-KO mice for each panels **F–J**). For all images, the dashed boxes indicate the subsets that are shown in Fig. 4.

 © The Author(s)

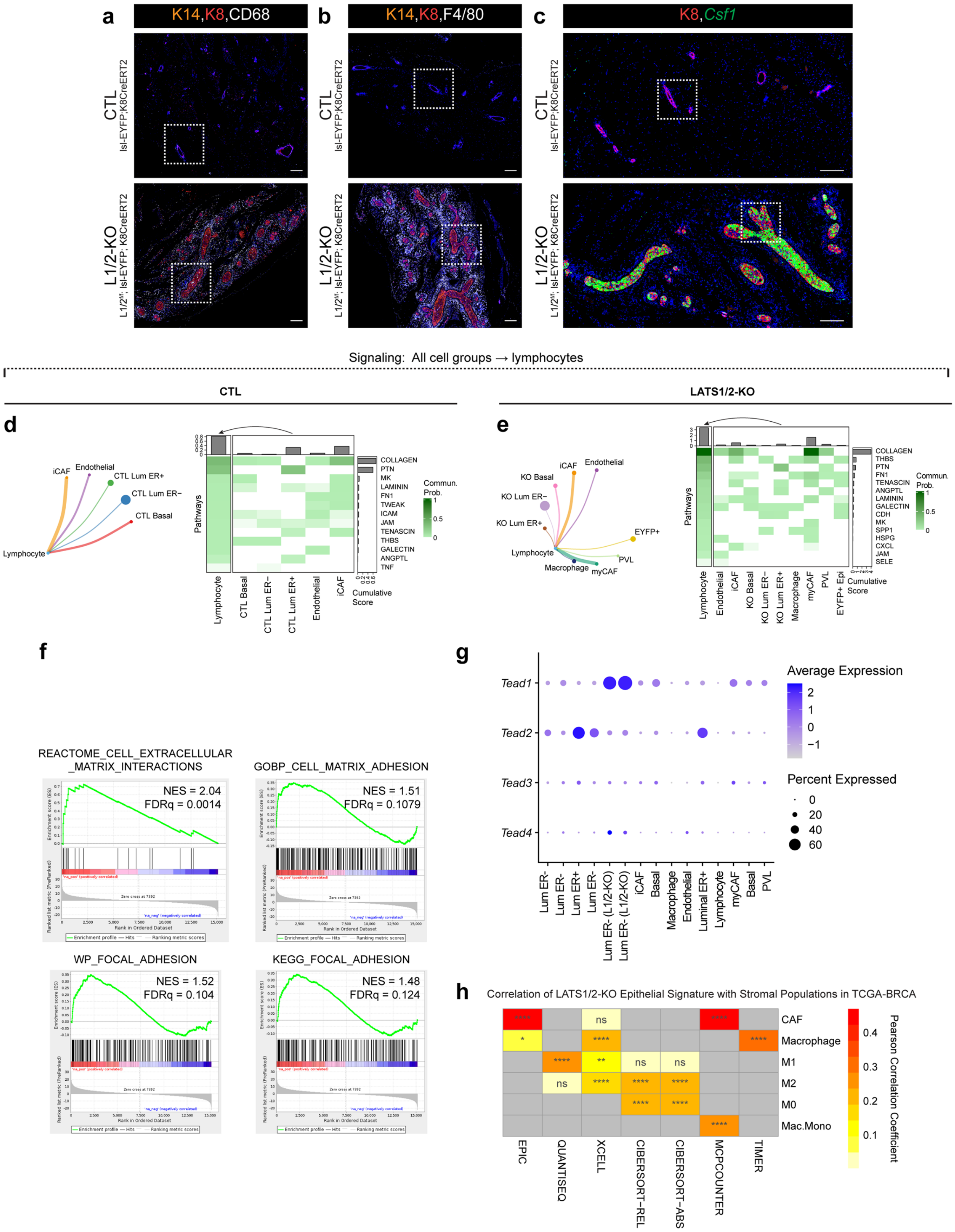
a
K14,K8,CD68
b
K14,K8,F4/80
c
K8,Csf1
CTL
lsl-EYFP;K8CreERT2
L1/2-KO
L1/2f/f; lsl-EYFP; K8CreERT2
Signaling: All cell groups → lymphocytes
CTL
LATS1/2-KO
d
iCAF
Endothelial
CTL Lum ER+
CTL Lum ER−
CTL Basal
Lymphocyte
Pathways
COLLAGEN
PTN
MK
LAMININ
FN1
TWEAK
ICAM
JAM
TENASCIN
THBS
GALECTIN
ANGPTL
TNF
Commun. Prob.
Cumulative Score
Lymphocyte
CTL Basal
CTL Lum ER−
CTL Lum ER+
Endothelial
iCAF
e
iCAF
Endothelial
KO Basal
KO Lum ER−
KO Lum ER+
EYFP+
Lymphocyte
PVL
Macrophage
myCAF
COLLAGEN
THBS
PTN
FN1
TENASCIN
ANGPTL
LAMININ
GALECTIN
CDH
MK
SPP1
HSPG
CXCL
JAM
SELE
Commun. Prob.
Cumulative Score
Lymphocyte
Endothelial
iCAF
KO Basal
KO Lum ER−
KO Lum ER+
Macrophage
myCAF
PVL
EYFP+ Epi
f
REACTOME_CELL_EXTRACELLULAR_MATRIX_INTERACTIONS
NES = 2.04
FDRq = 0.0014
GOBP_CELL_MATRIX_ADHESION
NES = 1.51
FDRq = 0.1079
WP_FOCAL_ADHESION
NES = 1.52
FDRq = 0.104
KEGG_FOCAL_ADHESION
NES = 1.48
FDRq = 0.124
g
Tead1
Tead2
Tead3
Tead4
Average Expression
Percent Expressed
Lum ER-
Lum ER-
Lum ER+
Lum ER-
Lum ER- (L1/2-KO)
Lum ER- (L1/2-KO)
iCAF
Basal
Macrophage
Endothelial
Luminal ER+
Lymphocyte
myCAF
Basal
PVL
h
Correlation of LATS1/2-KO Epithelial Signature with Stromal Populations in TCGA-BRCA
CAF
Macrophage
M1
M2
M0
Mac.Mono
EPIC
QUANTISEQ
XCELL
CIBERSORT-REL
CIBERSORT-ABS
MCPCOUNTER
TIMER
Pearson Correlation Coefficient

© The Author(s)

Figure EV4. Immune cell alterations in carcinomas driven by LATS1/2 inactivation and association of YAP/TAZ-TEAD with epithelial and stromal remodeling in mouse and human breast cancers.

(**A**) Immunofluorescence staining for KRT8, KRT14, and CD68 in CTL and LATS1/2-KO mammary glands. Dashed boxes indicate subsets shown in Fig. 5D (Scale bar, 200 μm) ($n = 6$ CTL mice, $n = 6$ L1/2-KO mice). (**B**) Immunofluorescence staining for KRT8, KRT14, and F4/80 in CTL and LATS1/2-KO mammary glands. Dashed boxes indicate subsets shown in Fig. 5E (Scale bar, 200 μm) ($n = 6$ CTL mice, $n = 6$ L1/2-KO mice). (**C**) RNAscope for *Csf1* along with IF for KRT8 in CTL and LATS1/2-KO mammary glands. Dashed boxes indicate subsets shown in Fig. 5I (Scale bar, 200 μm) ($n = 3$ CTL mice, $n = 3$ L1/2-KO mice). (**D**) CellChat circle plots showing communication networks from all cell groups (senders) to lymphocytes (receivers) in CTL and LATS1/2-KO mammary glands. Line widths represent the total number of interactions identified. (**E**) Heatmap of communication probabilities of signaling pathways from all cell groups to lymphocytes in CTL and LATS1/2-KO mammary glands. (**F**) GSEA enrichment plots of selected genesets enriched in sorted EYFP^{+} cells from the mammary glands of LATS1/2-KO (LATS1/2^{ff}; lsl-EYFP; Krt8CreERT2) mice relative to CTL (lsl-EYFP; Krt8CreERT2) mice. (**G**) Dot heatmap of TEAD transcription factors across all single-cell RNA-seq clusters. (**H**) TIMER Analysis of LATS1/2-KO epithelial signature correlated with presence of stromal cell populations in human TCGA samples. Gray boxes indicate that this cell type was not included in the indicated database. *fdr < 0.05, **fdr < 0.01, ****fdr <0.0001, ns = not significant.

 © The Author(s)

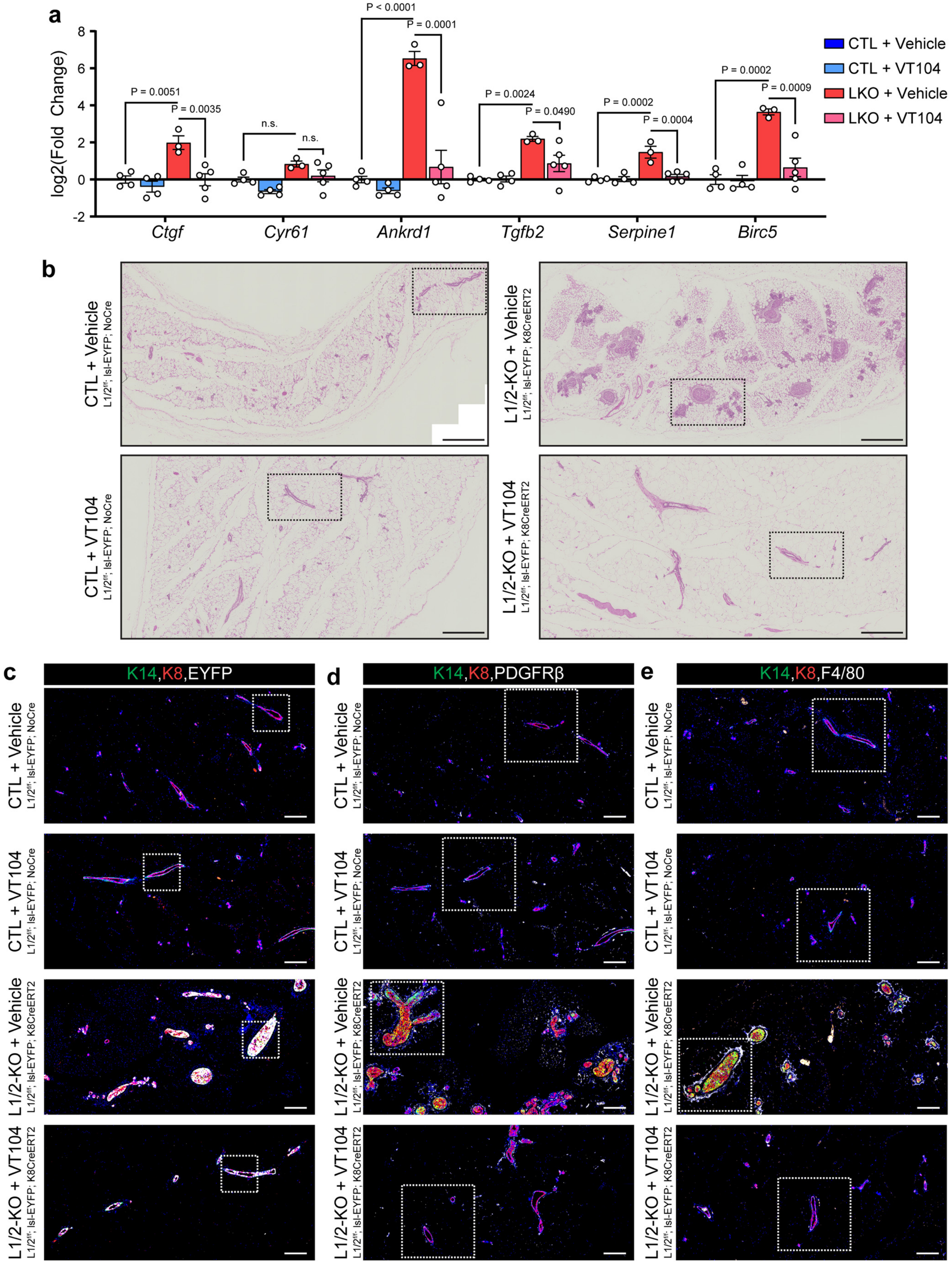
a
log2(Fold Change)
P = 0.0051
P = 0.0035
n.s.
n.s.
P < 0.0001
P = 0.0001
P = 0.0024
P = 0.0490
P = 0.0002
P = 0.0004
P = 0.0002
P = 0.0009
CTL + Vehicle
CTL + VT104
LKO + Vehicle
LKO + VT104
Ctgf
Cyr61
Ankrd1
Tgfb2
Serpine1
Birc5
b
CTL + Vehicle
CTL + VT104
L1/2-KO + Vehicle
L1/2-KO + VT104
c
K14,K8,EYFP
d
K14,K8,PDGFRβ
e
K14,K8,F4/80

Figure EV5. Pharmacological inhibition of YAP/TAZ reverses epithelial and stromal alterations in LATS1/2-null carcinomas.

(**A**) RT-qPCR analysis of YAP/TAZ-target gene expression in samples of epithelial and stromal cells collected from the 3rd and 5th mammary glands of CTL (Lats1/2$^{f/f}$; lsl-EYFP; NoCre) and L1/2-KO (Lats1/2$^{f/f}$; lsl-EYFP; K8CreERT2) mice treated with Vehicle or VT104 as outlined in Fig. 7A ($n = 4$ CTL + Vehicle mice, $n = 4$ CTL + VT104 mice, $n = 3$ L1/2-KO + Vehicle mice, $n = 5$ L1/2-KO + VT104 mice. Data are shown with mean ± SEM. Ordinary two-way ANOVAs with Tukey's multiple comparisons tests. Adjusted *P*-values are displayed in figure). (**B**) Hematoxylin and eosin stain of CTL (LATS1/2^{ff}; lsl-EYFP; noCre) and LATS1/2-KO (LATS1/2^{ff}; lsl-EYFP; Krt8CreERT2) mammary glands treated with Vehicle and VT104. Dashed boxes indicate subsets shown in Fig. 7A (Scale bar, 500 μm). (**C**) Immunofluorescence staining for KRT8, KRT14, and EYFP in mammary glands of CTL and LATS1/2-KO mice treated with Vehicle and VT104. Dashed boxes indicate subsets shown in Fig. 7B (Scale bar, 200 μm). (**D**) Immunofluorescence staining for KRT14, KRT8, and PDGFRβ in mammary glands of CTL and LATS1/2-KO mice treated with VT104. Dashed boxes indicate subsets shown in Fig. 7C (Scale bar, 200 μm). (**E**) Immunofluorescence staining for KRT14, KRT8, and F4/80 in mammary glands of CTL and LATS1/2-KO mice treated with VT104. Dashed boxes indicate subsets shown in Fig. 7D (Scale bar, 200 μm). For all panels: $n = 4$ CTL + Vehicle mice, $n = 4$ CTL + VT104 mice, $n = 3$ L1/2-KO + Vehicle mice, $n = 5$ L1/2-KO + VT104 mice.

 © The Author(s)
